# I_2_-catalyzed intramolecular oxidative amination of C(sp^3^)–H bond: efficient access to 3-acylimidazo[1,2-*a*]pyridines under neat condition†

**DOI:** 10.1039/c8ra10118c

**Published:** 2019-01-18

**Authors:** Lilan Huang, Wenqing Yin, Jian Wang, Chunfang Gan, Yanmin Huang, Chusheng Huang, Yimiao He

**Affiliations:** Guangxi Key Laboratory of Natural Polymer Chemistry and Physics, Nanning Normal University Nanning 530001 P. R. China heyimiao@gxtc.edu.cn huangcs@gxtc.edu.cn; Key Laboratory of Functional Molecular Solids, Ministry of Education, Anhui Laboratory of Molecule-Based Materials, College of Chemistry and Materials Science, Anhui Normal University Wuhu P. R. China

## Abstract

An efficient and “green” protocol for the synthesis of 3-acylimidazo[1,2-*a*]pyridines through intramolecular oxidative α-amination of carbonyl compounds has been developed. The reaction proceeds smoothly utilizing I_2_ as a catalyst and H_2_O_2_ as an oxidant under neat condition with broad substrate scope. Several complex nitrogen-containing fused rings are conveniently constructed, which are not easy to access by traditional methods.

## Abstract 2

An efficient and green protocol for the synthesis of 3-acylimidazo[1,2-*a*]pyridines utilizing I_2_ as a catalyst and H_2_O_2_ as an oxidant under neat condition or in water was developed.

## Graphical abstract

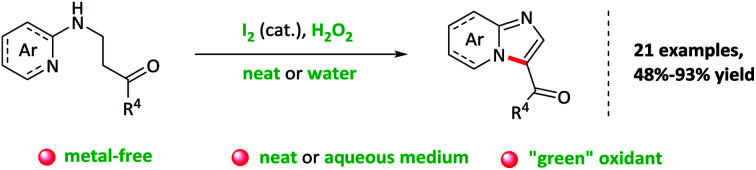

Intramolecular C–H bond activation to build C–X (N/O) bonds is of great significance in the construction of heterocyclic scaffolds, which could afford wide application for the direct synthesis of biologically active molecules and drug candidates.^1^ In contrast to unsaturated C–H bond activation reactions, transformations of Csp^3^–H bond to Csp^3^–X bonds are still limited.^2^ Over the past decades, several groups have developed transition-metal-catalysed intramolecular aliphatic C–H aminations and oxygenations, affording an atom-economic and efficient access to a series of N/O-containing heterocyclic compounds.^3^ Nevertheless, most of these methods face the limitations of transition metal residuals, expensive ligands, harsh conditions and narrow substrate scope. Recently, more environmentally-benign iodide-catalysed reactions for the preparation of heterocyclic compounds utilizing inexpensive and readily available TBHP or H_2_O_2_ as an oxidant have attracted enormous attention.^4^ For example, Ishihara and co-workers reported an intramolecular oxidative C–H bond α-oxygenation reaction of carbonyl compounds catalysed by *in situ* generated tetrabutylammonium (hypo)iodite with either hydrogen peroxide or *tert*-butyl hydroperoxide as a green oxidant (Scheme 1, eqn (1) and (2)).^5^ However, the application of this “green” catalytic cycle (iodide as the catalyst and H_2_O_2_ as the oxidant) for intramolecular α-amination of the carbonyl compounds, to the best of our knowledge, has been barely documented,^6^ although which allows a facile and “green” formation of a great bioactive *N*-heterocycles.

**Scheme 1:**
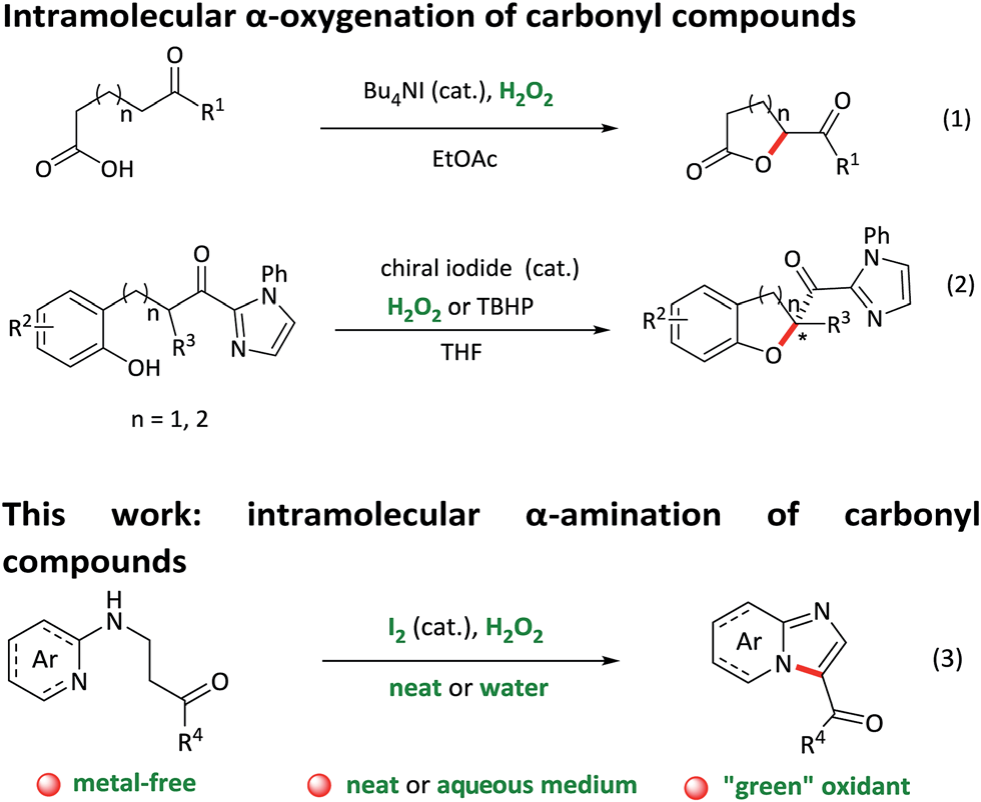
Intramolecular α-oxygenation and α-amination of carbonyl compounds.

Imidazo[1,2-*a*]pyridine represents an important heterocyclic scaffold with broad ranges of biological activities.^7^ Many commercially available drugs such as zolpidem, alpidem, zolimidine, olprinone, saripidem, and necopidem contain the core structure of imidazo[1,2-*a*]pyridine.^8^ Therefore, a variety of synthetic methods, including intramolecular aminooxygenation of alkenes or alkynes,^9^ tetrabutylammonium iodide catalyzed oxidative coupling reactions,^10^ copper-catalyzed aromatic aminations,^11^ three component couplings,^12^ and silver-catalyzed oxidative cross-coupling reactions,^13^ have been developed for the preparation of imidazo[1,2-*a*]pyridine derivatives. However, there are still some drawbacks which require to be solved in the traditional synthetic methods such as the use of complex starting materials, metal vestigial, narrow substrate scopes and harsh conditions. Therefore, the development of a metal-free, “green” and efficient method for the construction of imidazo[1,2-*a*]pyridine scaffold is still necessary.

Herein, we develop a convenient, efficient and “green” approach for the construction of 3-acylimidazo[1,2-*a*]pyridines through intramolecular oxidative α-amination of carbonyl compounds, which utilizing I_2_ as a catalyst and H_2_O_2_ as the only oxidant. To point out, the reaction proceeds under neat condition and the use of H_2_O_2_ as an oxidant generates H_2_O as the only by-product, which fully meet the requirement of green chemistry. Besides pyridine as a nitrogen source, the reaction undergoes equally efficiently with other *N*-heterocycles such as quinoline, pyrimidine, pyridazine and benzo[*d*]thiazole, thus providing a facile and environmentally sustainable pathway to prepare more complex nitrogen-containing fused rings.

Initially, the reaction was carried out by using 1-phenyl-3-(pyridin-2-ylamino)propan-1-one 1a as a model substrate in toluene with I_2_ as a catalyst and TBHP as an oxidant at 50 °C under air condition (Table 1, entry 1). Pleasingly, the reaction of 1a in the presence of 20 mol% catalyst with 2 equiv. of the oxidants gave the desired 2a in 71% yield. After screening various oxidants, H_2_O_2_ was found to be the most effective and provided 2a in 75% yield (entries 1–4). Replacing the catalyst I_2_ with other iodides such as TBAI, NaI did not improve the reaction (entry 5 and –6). Subsequently, various solvents were also evaluated and toluene revealed the best (entries 7–10). A further improvement was achieved upon elevating the temperature to 80 °C, allowing the reaction yield up to 87% (entry 11). The more important thing was that, the reaction could even undergo in water or under neat condition, giving a similar yield of 85% and 88% (entry 12–13). We also investigated the influence of the loadings of the catalyst on the reaction, and it revealed that increasing the amount of catalysts to 30 mol% did not improve the reaction, however, decreasing the amount of catalysts to 10 mol% resulted in a lower yield (entry 14 and 15). The blank experiments demonstrated that both catalyst and oxidant were essential for this reaction (entry 16 and 17). Consequently, the optimum reaction conditions were determined to be 1-phenyl-3-(pyridin-2-ylamino)propan-1-one 1a in the presence of I_2_ (20 mol%) as well as H_2_O_2_ (2 equiv.) under neat condition or in water at 80 °C.

**Table 1:** Optimization of reaction conditions^a^

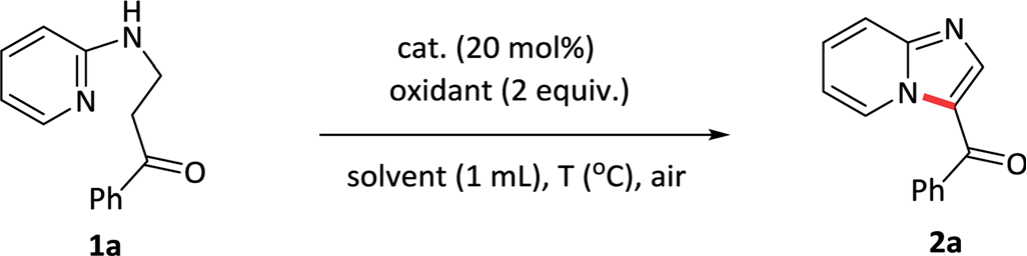					
Entry	Catalyst	Oxidant	Solvent	*T* [°C]	Yield^b^ [%]
1	I_2_	TBHP	Toluene	50	71
2	I_2_	H_2_O_2_	Toluene	50	75
3	I_2_	TBPB^c^	Toluene	50	15
4	I_2_	DTBP^d^	Toluene	50	11
5	TBAI	H_2_O_2_	Toluene	50	56
6	NaI	H_2_O_2_	Toluene	50	54
7	I_2_	H_2_O_2_	THF	50	61
8	I_2_	H_2_O_2_	DCM	50	63
9	I_2_	H_2_O_2_	CH_3_CN	50	52
10	I_2_	H_2_O_2_	DMF	50	Trace
11	I_2_	H_2_O_2_	Toluene	80	87
**12**	**I**_**2**_	**H**_**2**_ **O**_**2**_	**H**_**2**_ **O**	**80**	**85**
**13**	**I**_**2**_	**H**_**2**_ **O**_**2**_	**Neat**	**80**	**88**
14^e^	I_2_	H_2_O_2_	H_2_O	80	86
15^f^	I_2_	H_2_O_2_	H_2_O	80	67
16^g^	I_2_		H_2_O	80	trace
17^h^		H_2_O_2_	H_2_O	80	trace

a Reaction conditions: 1a (0.2 mmol), catalyst (20 mol%), oxidant (0.4 mmol) in solvent (1 mL), air. TBHP (70 wt% in H_2_O), H_2_O_2_ (30 wt% in H_2_O).

b Isolated yields.

c TBPB = *t*-butylperoxybenzoate.

d DTBP = di-*tert*-butyl peroxide.

e Catalyst (30 mol%).

f Catalyst (10 mol%).

g Without oxidant.

h Without catalyst.

With optimized conditions in hand, various substituents at pyridine component of 1-phenyl-3-(pyridin-2-ylamino)propan-1-ones 1b–1i were first tested under standard conditions to form the corresponding imidazo[1,2-*a*]pyridin-3-yl(phenyl)methanone 2b–2i. As showed in Table 2, pyridines bearing electron-rich groups (2b, 2h–2i) such as methyl, methoxy at the *meta*- or *para*-position underwent an oxidative cycloamination process smoothly to afford the desired products in good yields (81–93%). Slightly lower yields were achieved for weak electron-withdrawing groups at pyridine component such as fluoro, chloro, bromo substituents, maybe due to the electronic effect (2c–2e). Several strong electron-withdrawing groups, such as trifluoromethyl (2f) and ester (2g) substituents, the yield sharply decreased to 48% and 67%, respectively. These results indicated that electronic effect exerted by the substituents in pyridine component had an important influence on this reaction. In addition, further transformations could be made for halogen-substituent in pyridine component through the cross-coupling reactions.

**Table 2:** Substrate scope with substituents at the pyridine component^a^^,^^b^

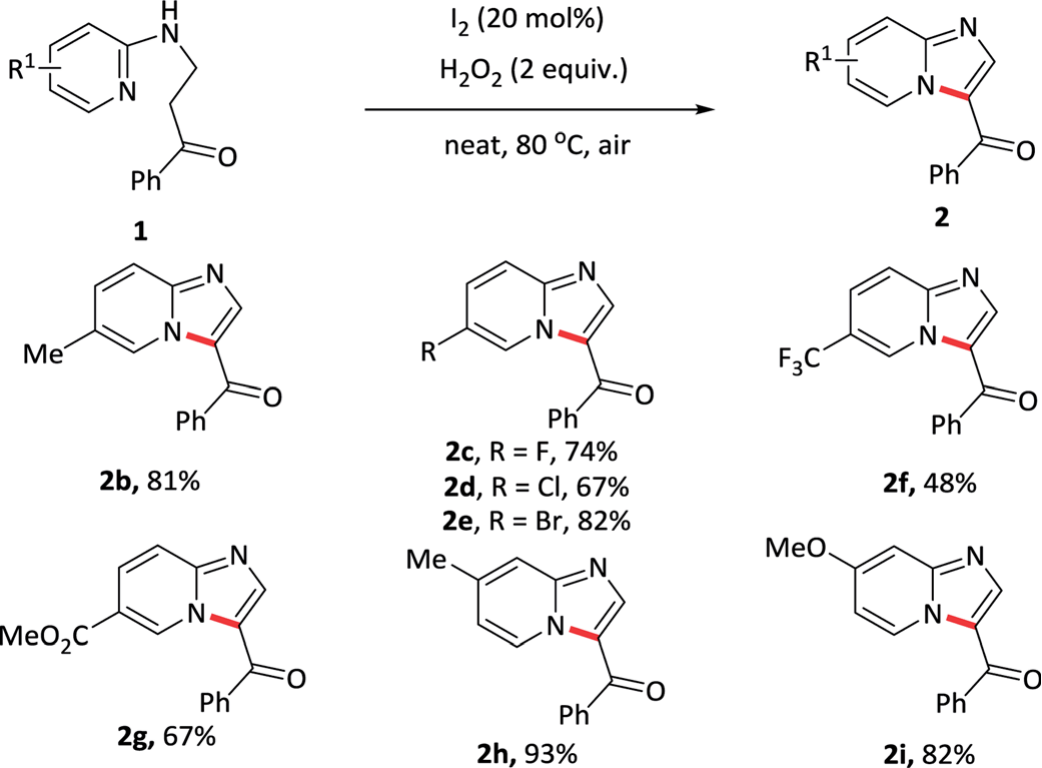

a Reaction conditions: 1 (0.2 mmol), I_2_ (0.04 mmol), H_2_O_2_ (0.4 mmol), at 80 °C for 0.5–6 h.

b Isolated yields.

The substrates bearing various substituents at the carbonyl terminal position were subsequently evaluated (Table 3). Groups such as electron-rich methyl, electron-withdrawing fluoro, chloro, bromo substituents on the arene ring were tolerant well and afforded the desired products in similar yields (2j–2n), suggesting that electronic effect exerted by the substituents at the terminal of the carbonyl component had almost no effect on this reaction. Exchanging the phenyl ring to other aromatic rings, such as thiophene, gave the product 2o in 83% yield. Furthermore, alkyl substituents were also compatible with this reaction and received the target products in moderate to excellent yields (2p–2q). However, the reaction did not proceed when the substrate carried with the ester substituent at the terminal (2r).

**Table 3:** Substrate scope with substituents at the carbonyl terminal position^a^^,^^b^

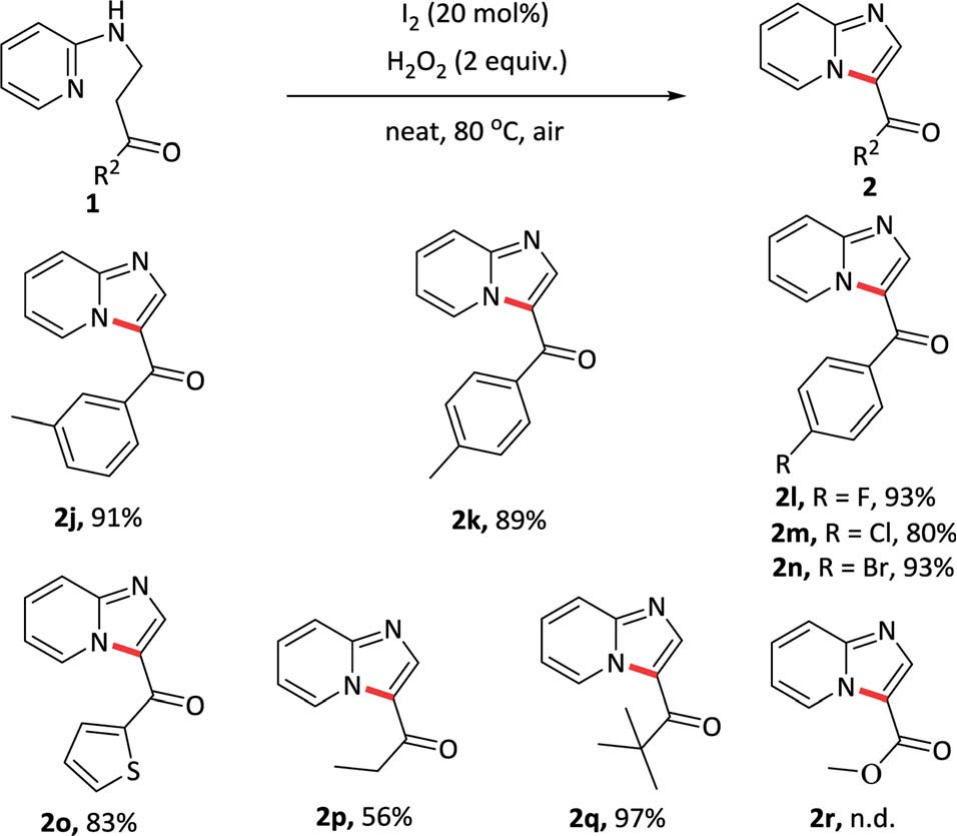

a Reaction conditions: 1 (0.2 mmol), I_2_ (0.04 mmol), H_2_O_2_ (0.4 mmol), at 80 °C for 0.5–6 h.

b Isolated yields.

To elaborate the substrate universalities of this strategy for the intramolecular oxidative amination reaction, substrates bearing other azaheterocyclic arenes other than the pyridine were also prepared and the results were displayed in Table 4. The substrate carrying with quinoline ring 2s gave the desired product in excellent yield under standard conditions. Other nitrogen-containing hexatomic rings, such as pyrimidine and pyridazine, delivered the target outcomes 2t and 2u in 74% and 83% yields, respectively. In addition, the five-membered azaheterocyclic arene such as benzo[*d*]thiazole, also was tolerant well and obtained the desired products 2v in 82% yields. The above-mentioned nitrogen-containing fused ring derivatives 2s–2v were not easy to prepare in conventional methods.

**Table 4:** Substrate scope with other azaheterocyclic arenes^a^^,^^b^

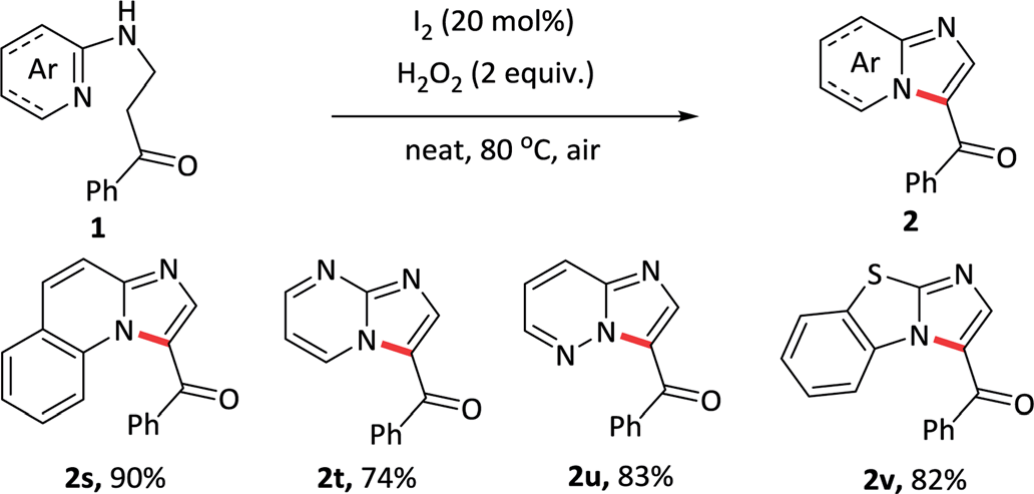

a Reaction conditions: 1 (0.2 mmol), I_2_ (0.04 mmol), H_2_O_2_ (0.4 mmol), at 80 °C for 0.5–6 h.

b Isolated yields.

Further elaborations of 3-acylimidazo[1,2-*a*]pyridines were subsequently conducted; several representative examples were provided in Table 5. The acyl group could be transformed to the methylene, as well as several functionalization were effectively received in α position of carbonyl groups, such as bromination, arylation, alkenylation, amination and iodization.

**Table 5:** Diversification of 3-acylimidazo[1,2-*a*]pyridines

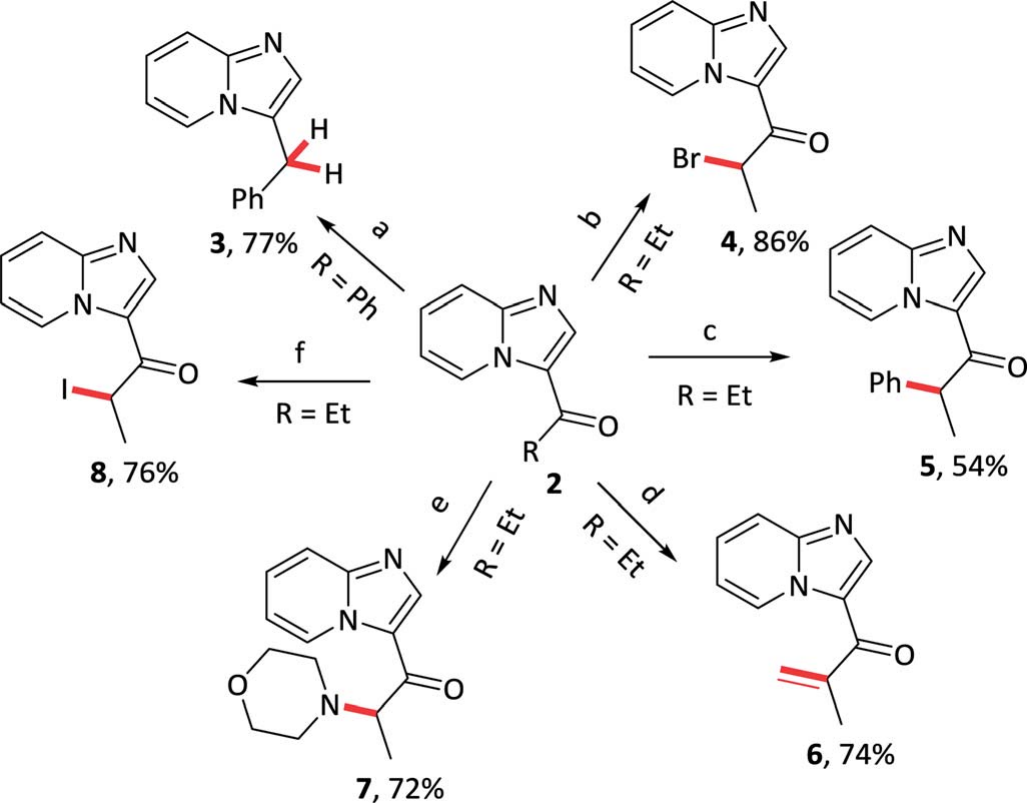

a Reaction conditions: 2a (0.2 mmol), N_2_H_4_ (2 equiv.), toluene (1 mL), μW; then KOH (7 equiv.), μW.

b 2p (0.2 mmol), NBS (1.2 equiv.), *p*-TsOH·H_2_O (0.2 equiv.), CH_3_CN (1 mL), 60 °C.

c 2p (2 equiv.), PhI (0.1 mmol), *t*-BuOK (5 equiv.), DMF (1 mL), 60 °C.

d 2p (0.1 mmol), DABCO (0.5 equiv.), K_2_S_2_O_8_ (2 equiv.), DMSO (1 mL), 120 °C.

e 2p (0.2 mmol), NBS (1.4 equiv.), PTSA (1 equiv.), CH_3_CN (1 mL), 60 °C; then morpholine (3 equiv.), K_2_CO_3_ (2.5 equiv.), CH_3_CN (1 mL), rt.

f 2p (0.2 mmol), NBS (1.4 equiv.), PTSA (1 equiv.), CH_3_CN (1 mL), 60 °C; then NaI (1.1 equiv.), acetone (1 mL), rt.

The scalability of this approach was verified by running the reaction of 1a on both 4 and 10 mmol scales (Scheme 2). 2a was isolated in acceptable yields in both of the cases.

**Scheme 2:**
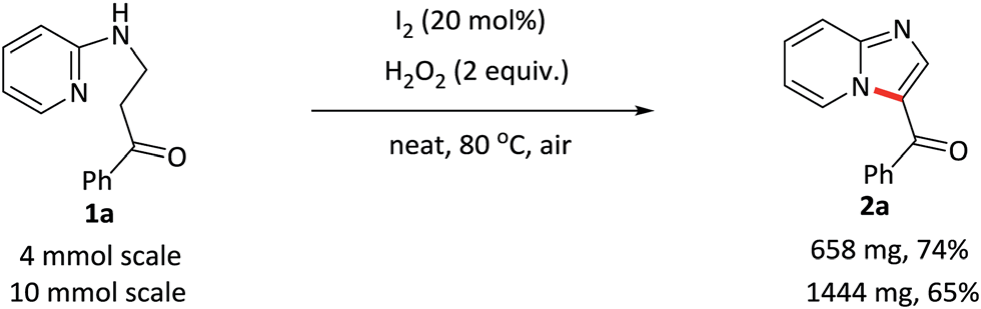
Large-scale synthesis of 2a.

The mechanism for this intramolecular oxidative amination reaction was subsequently studied. Firstly, model substrate 1a was subjected to the hypervalent iodide reagents PhI(OAc)_2_ or Bu_4_NIO_3_ or Bu_4_NIO_4_, and the targeted molecule 2a was not detected along with recovery of 1a in 91% yield (page S24,† eqn (1)). Subsequently, increasing the loadings of I_2_ from 0.2 equiv. to 2 equiv. in the absence of H_2_O_2_, the reaction turned out to be messy (page S24,† eqn (2)). Furthermore, when TEMPO, a radical scavenger, was added into the system, the reaction was not suppressed and the targeted product 2a was delivered in 73% yield (S5,† eqn (3)), which indicated that the reaction probably didn't involve a radical process.

Based on the above results and previous reports,^14^ a plausible mechanism pathway for I_2_/H_2_O_2_-catalyzed synthesis of 3-acylimidazo[1,2-*a*]pyridines 2a starting from 1-phenyl-3-(pyridin-2-ylamino)propan-1-one 1a is depicted in Scheme 3. In the first step, I_2_ or the released HI is oxidized by H_2_O_2_ to form HOI. In the second step, 1a reacts with HOI to produce a possible intermediate A or B, which then experiences a similar SN_2_ process to receive a cyclic intermediate C with release of HI. The intermediate C is oxidized by HOI and finally obtain the target product 2a.

**Scheme 3:**
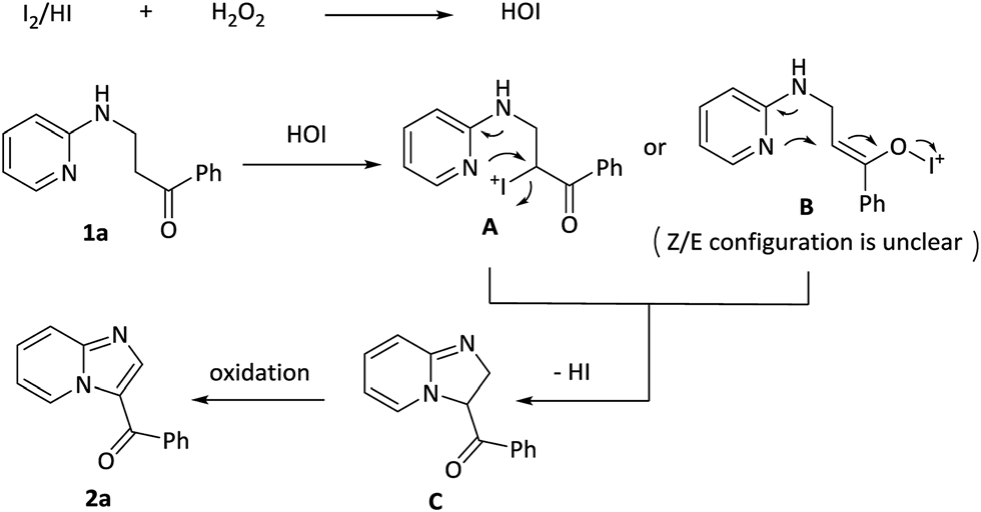
Proposed mechanism.

In summary, we have developed a general and efficient method for the construction of 3-acylimidazo[1,2-*a*]pyridines through intramolecular oxidative α-amination of carbonyl compounds under metal-free conditions. The reaction proceeded smoothly with I_2_ as a catalyst and H_2_O_2_ as an oxidant under neat condition. This protocol exhibits general substrate scope and several complex nitrogen-containing fused rings could be conveniently accessed *via* this approach.

## Conflicts of interest

There are no conflicts to declare.

## Supplementary Material

RA-009-C8RA10118C-s001
