# Supplementary material for: I2-catalyzed intramolecular oxidative amination of C(sp3)–H bond: efficient access to 3-acylimidazo[1,2-a]pyridines under neat condition
Source: RSC Adv. 2019 Jan 18;9(5):2381–5. doi: 10.1039/c8ra10118c (PMC9059843; doi:10.1039/c8ra10118c)
Supplement: RA-009-C8RA10118C-s001 [file RA-009-C8RA10118C-s001.pdf]

Electronic Supplementary Material (ESI) for RSC Advances.
This journal is © The Royal Society of Chemistry 2019

Electronic Supplementary Information (ESI)

I_2-Catalyzed Intramolecular Oxidative Amination of C(sp^3)-H Bond: Efficient Access to 3-Acylimidazo[1,2-*a*]pyridines Under Neat Condition

Lilan Huang,[+a] Wenqing Yin, [+a] Jian Wang,[b] Chunfang Gan,[a] Yanmin Huang,[a] Chusheng Huang,[*a] Yimiao He[*a]

a. Guangxi Key Laboratory of Natural Polymer Chemistry and Physics, Nanning Normal University, Nanning 530001, P. R. China. E-mail: heyimiao@gxtc.edu.cn; huangcs@gxtc.edu.cn.

b. Key Laboratory of Functional Molecular Solids, Ministry of Education, Anhui Laboratory of Molecule-Based Materials, College of Chemistry and Materials Science, Anhui Normal University, Wuhu, P. R. China

Table of contents

1. General information......2

2. Synthesis and characterization of starting materials 1a-1v 2

2.1 Synthesis of starting materials **1a-1v** 2

2.2 Product characterization......4

3. General procedure and product characterization 12

3.1 General procedure 12

3.2 Product Characterization......13

4. Diversification of 3-acylimidazo[1,2-a]pyridines......20

5. Contral Experiments......24

6. References 24

7. Copies of 1H NMR and 13C NMR Spectra 26

1. General information

Reactions were monitored by using thin-layer chromatography (TLC) on commercial silica gel plates (GF254). Visualization of the developed plates was performed under UV lights (254 nm). Flash column chromatography was performed on silica gel (200-300 mesh). ^{1}H and ^{13}C NMR spectra were recorded on Bruker AV300，400, 500 and 600 MHz spectrometers. Chemical shifts (δ) were reported in ppm referenced to the $CDCl_3$ residual peak (δ 7.26) or the DMSO-d_6 residual peak (δ 2.50) for ^{1}H NMR. Chemical shifts of ^{13}C NMR were reported relative to $CDCl_3$ (δ 77.0) or D_6-DMSO (δ 39.5). The following abbreviations were used to describe peak splitting patterns when appropriate: br s = broad singlet, s = singlet, d = doublet, t = triplet, q = quartet, m = multiplet. Coupling constant, *J*, was reported in Hertz unit (Hz). Melting points (mp) were taken on a MEL-TEMP® apparatus and were uncorrected. High resolution mass spectra (HRMS) were obtained on an ESI-LC-MS/MS spectrometer.

2. Synthesis and characterization of starting materials 1a-1v

2.1 Synthesis of starting materials 1a-1v

Method I: The material **1a** was prepared according to the following method.

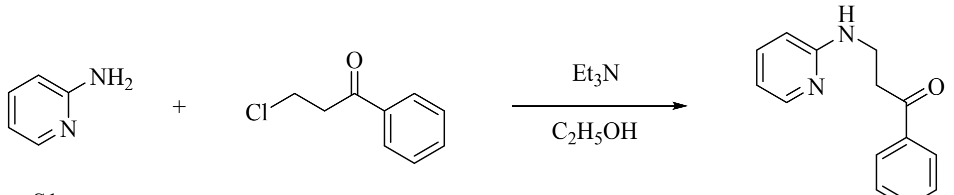

S1 **S2** **1a**

1-phenyl-3-(pyridin-2-ylamino)propan-1-one (1a). A sealed tube was equipped with a magnetic stir bar and was charged with pyridin-2-amine **S1** (339 mg，3.6 mmol), 3-chloro-1-phenylpropan-1-one **S2** (506 mg，3.0 mmol), triethylamine (0.6 mL, 4.2 mmol) in ethanol (2 mL). The reaction mixture was stirred under 150 W microwave irradiation at 100 ^{o}C for 5 minutes. After the reaction was complete (as judged by TLC analysis), the solution was cooled to room temperature and extracted with EtOAc (20 mL) and washed with brine for three times. Then the combined organic layers were dried over Na_2SO_4 and removed the volatiles in vacuo. The residues were purified by column chromatography on silica gel (petroleum ether/EtOAc = 5 : 1) to afford the desired product 1-phenyl-3-(pyridin-2-ylamino)propan-1-one **1a** (461 mg, 68% yield) as a white solid.

1b-1i, **1l-1n**, **1r-1v** were prepared according to the procedure described for **1a**.

Method II: The material **1j** was prepared according to the following method.

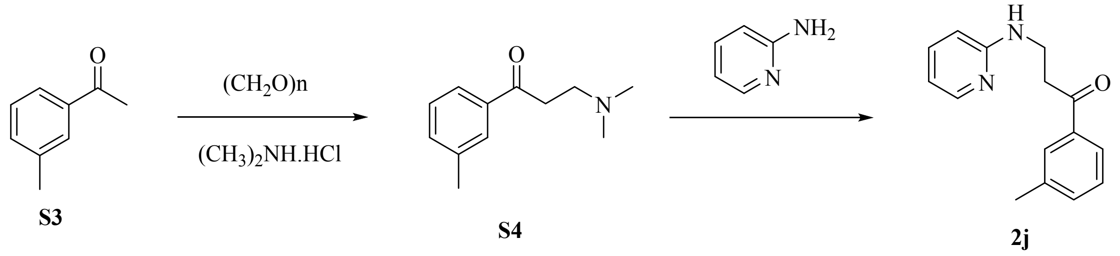

A sealed tube was equipped with a magnetic stir bar and was charged with 1-(*m*-tolyl)ethan-1-one **S3** (402 mg, 3 mmol), paraformaldehyde (900 mg), dimethylamine hydrochloride (489 mg, 6 mmol) in ethanol (2 mL). The reaction mixture was stirred under 150 W microwave irradiation at 100 °C until the color turned out to be yellow. The solution was cooled to room temperature, to which a sat. aqueous $NaHCO_3$ was added dropwise until there was no CO_2 release. Then the mixture was extracted with EtOAc (20 mL) and washed with brine for three times, and the resulted organic layers were dried over Na_2SO_4 and removed the volatiles under reduced pressure to give the crude product **S4**.

A sealed tube was equipped with a magnetic stir bar and charged with 1-(*m*-tolyl)ethan-1-one **S4**, pyridin-2-amine (338 mg, 3.6 mmol) in ethanol (2 mL). The reaction mixture was stirred under 150 W microwave irradiation at 100 °C until the reaction was complete (as judged by TLC analysis). Then the reaction mixture was cooled to room temperature and extracted with EtOAc (20 mL) and washed with brine for three times, and the resulted organic layers were dried over Na_2SO_4 and removed the volatiles under reduced pressure. The residues were purified by column chromatography on silica gel (petroleum ether/EtOAc = 5 : 1) to give the desired product **1j** (389 mg, 54% yield) as a white solid.

1k, 1o and **1q** were prepared according to procedures described for **1j**.

Method III: The material **1p** was prepared according to the following method.

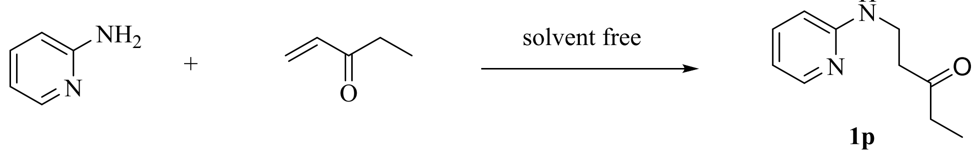

A sealed tube was equipped with a magnetic stir bar and charged with pyridin-2-amine (283 mg, 3 mmol), ethyl vinyl ketone (379mg，4.5mmol) and stirred overnight at room temperature. After the reaction was complete (as determined by TLC analysis), the reaction mixture was extracted with EtOAc (20 mL) and washed with brine for three

times, and the resulted organic layers were dried over Na_2SO_4 and evaporated under reduced pressure. The residues were purified by column chromatography on silica gel (petroleum ether/EtOAc = 5 : 1) to give the desired product **1p** (401 mg, 75% yield) as a white solid.

2.2 Product characterization

1-phenyl-3-(pyridin-2-ylamino)propan-1-one (1a)

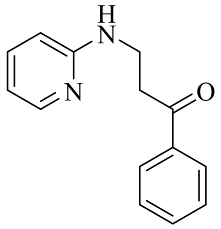

Yield: 57%. Mp 82-84 °C. White solid. ^{1}H NMR (300 MHz, $CDCl_3$): δ 8.10 (d, J = 5.1 Hz, 1H), 8.00-7.96 (m, 2H), 7.59 (t, J = 5.1 Hz, 1H), 7.56-7.47 (m, 2H), 7.45-7.36 (m, 1H), 6.58-6.54 (m, 1H), 6.41 (d, J = 8.4 Hz, 1H), 4.97 (br s, 1H), 3.84 (t, J = 6.0 Hz, 2H), 3.34 (t, J = 6.0 Hz, 2H); ^{13}C NMR (75 MHz, $CDCl_3$): δ 199.6, 158.3, 147.9, 137.3, 136.8, 133.3, 128.6, 128.1, 112.8, 108.0, 38.2, 36.6; HRMS (ESI): Exact mass calcd for $C_{14}H_{14}N_2O$ $[M+H]^+$, 227.1179; Found: 227.1176.

3-((5-methylpyridin-2-yl)amino)-1-phenylpropan-1-one (1b)

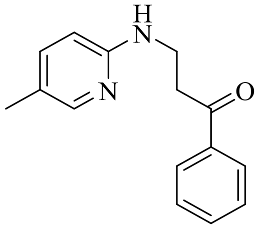

Yield: 61%. Mp 96-97 °C. White solid. ^{1}H NMR (300 MHz, $CDCl_3$): δ 8.00-7.93 (m, 3H), 7.60-7.54 (m, 1H), 7.49-7.44 (m, 2H), 7.23 (d, J = 8.7 Hz, 1H), 6.35 (d, J = 8.4 Hz, 1H), 4.80 (br s, 1H), 3.80 (t, J = 6.0 Hz, 2H), 3.32 (t, J = 6.0 Hz, 2H), 2.18 (s, 3H); ^{13}C NMR (150 MHz, $CDCl_3$): δ 199.7, 156.5, 147.4, 138.4, 136.8, 133.3, 128.6, 128.0, 121.6, 107.7, 38.2, 36.9, 17.4; HRMS (ESI): Exact mass calcd for $C_{15}H_{16}N_2O$ $[M+H]^+$, 241.1335; Found: 241.1332.

3-((5-fluoropyridin-2-yl)amino)-1-phenylpropan-1-one (1c)

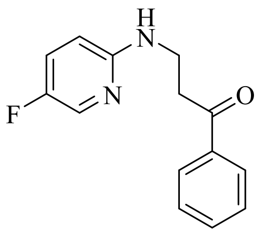

Yield: 71%. Mp 93-94 ºC. White solid. ^{1}H NMR (300 MHz, $CDCl_3$): δ 8.00-7.96 (m, 2H), 7.62-7.56 (m, 1H), 7.51-7.45 (m, 2H), 7.21-7.15 (m, 2H), 6.39-6.35 (m, 1H), 4.91 (br s, 1H), 3.80 (t, J = 12.3 Hz, 2H), 3.32 (t, J = 6.0 Hz, 2H); ^{13}C NMR (150 MHz, $CDCl_3$): δ 199.8, 155.0, 153.9, 152.6, 136.7, 134.3, 134.2, 133.4, 128.6, 128.0, 125.3, 125.2, 110.0, 108.4, 38.0, 37.1; HRMS (ESI): Exact mass calcd for $C_{14}H_{13}FN_2O$ $[M+H]^+$, 245.1085; Found: 245.1087.

3-((5-chloropyridin-2-yl)amino)-1-phenylpropan-1-one (1d)

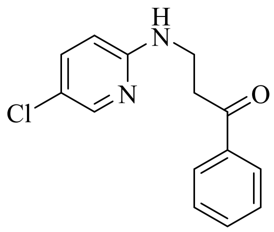

Yield: 72%. Mp 110-112 ºC. Pale yellow solid. ^{1}H NMR (600 MHz, $CDCl_3$): δ 8.01 (s, 1H), 7.94 (d, J = 8.4 Hz, 2H), 7.57-7.54 (m, 1H), 7.44 (t, J = 7.8 Hz, 2H), 7.29 (d, J = 8.4 Hz, 1H), 6.32 (d, J = 8.4 Hz, 1H), 5.00 (br s, 1H), 3.78 (t, J = 6.0 Hz, 2H), 3.29 (t, J = 5.4 Hz, 2H); ^{13}C NMR (150 MHz, $CDCl_3$): δ 199.5, 156.6, 146.2, 137.0, 136.6, 133.4, 128.7, 128.0, 119.6, 108.9, 37.9, 36.7; HRMS (ESI): Exact mass calcd for $C_{14}H_{13}ClN_2O$ $[M+H]^+$, 261.0789; Found: 261.0792.

3-((5-bromopyridin-2-yl)amino)-1-phenylpropan-1-one (1e)

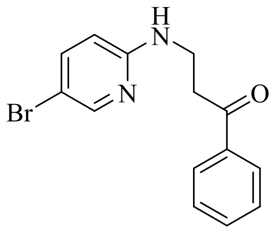

Yield: 72%. Mp 110-112 ºC. Pale yellow solid. ^{1}H NMR (300 MHz, $CDCl_3$): δ 8.13 (s, 1H), 8.00-7.96 (m, 2H), 7.62-7.56 (m, 1H), 7.50-7.42 (m, 3H), 6.32 (d, J = 9.0 Hz, 1H), 5.04 (br s, 1H), 3.81 (t, J = 6.0 Hz, 2H), 3.32 (t, J = 6.0 Hz, 2H); ^{13}C NMR (75 MHz,

$CDCl_3$): δ 199.5, 156.9, 148.5, 139.6, 136.7, 133.4, 128.7, 128.0, 109.6, 106.9, 38.0, 36.7; HRMS (ESI): Exact mass calcd for $C_{14}H_{13}BrN_2O$ $[M+H]^+$, 305.0290; Found: 305.0287.

phenyl-3-((5-(trifluoromethyl)pyridin-2-yl)amino)propan-1-one (1f)

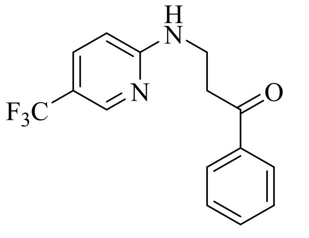

Yield: 69%. Mp 126-128 °C. White solid. ^1H NMR (300 MHz, $CDCl_3$): δ 8.35 (s, 1H), 8.00-7.96 (m, 2H), 7.63-7.46 (m, 4H), 6.42 (d, J = 8.7 Hz, 1H), 5.40 (br s, 1H), 3.90 (t, J = 6.0 Hz, 2H), 3.35 (t, J = 5.7 Hz, 2H); ^{13}C NMR (150 MHz, $CDCl_3$): δ 199.3, 159.8, 145.9, 134.1, 133.5, 128.7, 128.0, 107.4, 47.4, 37.9, 36.3; HRMS (ESI): Exact mass calcd for $C_{15}H_{13}F_3N_2O$ $[M+H]^+$, 295.1053; Found: 295.1049.

methyl 6-((3-oxo-3-phenylpropyl)amino)nicotinate (1g)

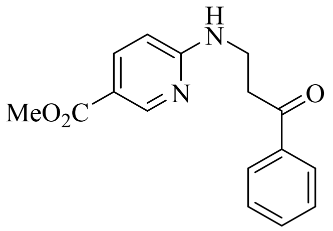

Yield: 48%. Mp 161-162 °C. White solid. ^1H NMR (300 MHz, $CDCl_3$): δ 8.77 (s, 1H), 7.99-7.93 (m, 3H), 7.59 (t, J = 7.5 Hz, 1H), 7.47 (t, J = 7.8 Hz, 2H), 6.37 (d, J = 8.7 Hz, 1H), 5.55 (br s, 1H), 3.94-3.87 (m, 5H), 3.35 (t, J = 5.7 Hz, 2H); ^{13}C NMR (150 MHz, $CDCl_3$): δ 205.3, 166.4, 160.3, 151.4, 138.1, 133.5, 128.7, 128.0, 115.0, 51.6, 38.0, 36.4; HRMS (ESI): Exact mass calcd for $C_{16}H_{16}N_2O_3$ $[M+H]^+$, 285.1234; Found: 285.1229.

3-((4-methylpyridin-2-yl)amino)-1-phenylpropan-1-one (1h)

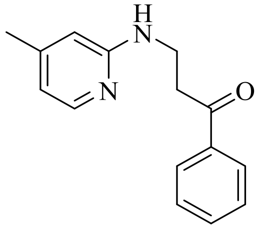

Yield: 65%. Mp 88-89 °C. White solid. ^{1}H NMR (300 MHz, $CDCl_3$): δ 8.00-7.96 (m, 3H), 7.61-7.55 (m, 1H), 7.50-7.44 (m, 2H), 6.42 (d, J = 5.1 Hz, 1H), 6.23 (s, 1H), 4.87 (br s, 1H), 3.85-3.78 (m, 2H), 3.53-3.31 (m, 2H), 2.22 (s, 3H); ^{13}C NMR (150 MHz, $CDCl_3$): δ 199.6, 158.5, 148.2, 147.6, 136.7, 133.3, 128.6, 128.0, 114.5, 108.1, 38.2, 36.6, 21.1; HRMS (ESI): Exact mass calcd for $C_{15}H_{16}N_2O$ $[M+H]^+$, 241.1335; Found: 241.1334.

3-((4-methoxypyridin-2-yl)amino)-1-phenylpropan-1-one (1i)

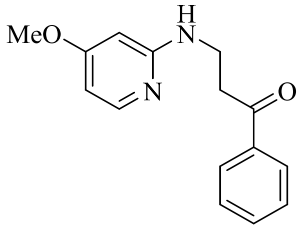

Yield: 63%. Mp 106-107 °C. White solid. ^{1}H NMR (300 MHz, $CDCl_3$): δ 7.97-7.92 (m, 3H), 7.59-7.54 (m, 1H), 7.45 (t, J = 7.2 Hz, 2H), 6.20 (d, J = 6.0 Hz, 1H), 5.87 (s, 1H), 5.00 (br s, 1H), 3.82-3.76 (m, 5H), 3.31 (t, J = 6.0 Hz, 2H); ^{13}C NMR (75 MHz, $CDCl_3$): δ 199.6, 167.0, 160.2, 149.1, 136.7, 133.3, 128.6, 128.0, 101.7, 91.2, 54.9, 38.2, 36.8; HRMS (ESI): Exact mass calcd for $C_{15}H_{16}N_2O_2$ $[M+H]^+$, 257.1285; Found: 257.1283.

3-(pyridin-2-ylamino)-1-(m-tolyl)propan-1-one (1j)

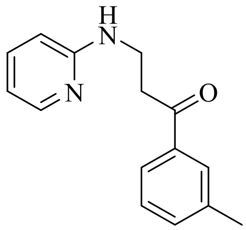

Yield: 30%. Mp 87-88 °C. White solid. ^{1}H NMR (300 MHz, $CDCl_3$): δ 8.11 (d, J = 5.4 Hz, 1H), 7.79-7.76 (m, 2H), 7.42-7.35 (m, 3H), 6.59-6.56 (m, 1H), 6.40 (d, J = 8.4 Hz, 1H), 4.93 (br s, 1H), 3.82 (t, J = 6.0 Hz, 2H), 3.32 (t, J = 6.0 Hz, 2H), 2.42 (s, 3H); ^{13}C NMR (150 MHz, $CDCl_3$): δ 199.8, 147.9, 137.2, 136.7, 134.1, 128.6, 128.5, 125.2, 112.7, 108.0, 38.2, 36.6, 21.3; HRMS (ESI): Exact mass calcd for $C_{15}H_{16}N_2O$ $[M+H]^+$, 241.1335; Found: 241.1340.

3-(pyridin-2-ylamino)-1-(p-tolyl)propan-1-one (1k)

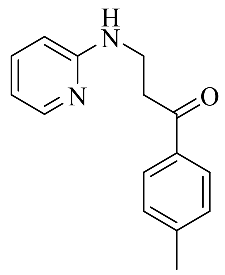

Yield: 33%. Yellow oil. ^{1}H NMR (600 MHz, $CDCl_3$): δ 8.07 (d, *J* = 5.4 Hz, 1H), 7.75 (t, *J* = 7.2 Hz, 2H), 7.37-7.31 (m, 3H), 6.53 (t, *J* = 6.0 Hz, 1H), 6.37 (d, *J* = 8.4 Hz, 1H), 4.92 (br s, 1H), 3.78 (t, *J* = 6.0 Hz, 2H), 3.29 (t, *J* = 6.0 Hz, 2H), 2.39 (s, 3H); ^{13}C NMR (150 MHz, $CDCl_3$): δ 199.8, 158.3, 147.9, 138.4, 137.3, 136.7, 134.1, 128.6, 128.5, 125.3, 112.8, 108.0, 38.2, 36.6, 21.3; HRMS (ESI): Exact mass calcd for $C_{15}H_{16}N_2O$ $[M+H]^+$, 241.1335; Found: 241.1340.

1-(4-fluorophenyl)-3-(pyridin-2-ylamino)propan-1-one (1l)

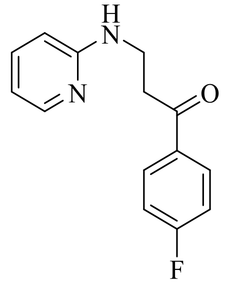

Yield: 80%. Mp 85-86 °C. Pale yellow solid. ^{1}H NMR (600 MHz, $CDCl_3$): δ 8.04 (s, 1H), 7.94-7.91 (m, 2H), 7.32-7.29 (m, 1H), 7.07-7.03 (m, 2H), 6.49 (t, *J* = 7.2 Hz, 1H), 6.34 (d, *J* = 8.4 Hz, 1H), 5.08 (br s, 1H), 3.75 (t, *J* = 6.0 Hz, 2H), 3.23 (t, *J* = 6.0 Hz, 2H); ^{13}C NMR (150 MHz, $CDCl_3$): δ 198.0, 166.6, 164.9, 158.2, 147.9, 137.2, 133.2, 130.7, 130.6, 115.8, 115.6, 112.8, 108.0, 38.1, 36.6; HRMS (ESI): Exact mass calcd for $C_{14}H_{13}FN_2O$ $[M+H]^+$, 245.1085; Found: 245.1087.

1-(4-chlorophenyl)-3-(pyridin-2-ylamino)propan-1-one (1m)

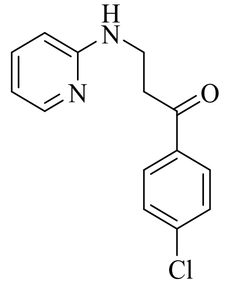

Yield: 62%. Mp 100-101 °C. White solid. ^{1}H NMR (300 MHz, CDCl$_3$): δ 8.10 (d, *J* = 6.9 Hz, 1H), 7.95-7.90 (m, 2H), 7.47-7.36 (m, 3H), 6.59-6.55 (m, 1H), 6.40 (d, *J* = 8.4 Hz, 1H), 4.90 (br s, 1H), 3.83 (t, *J* = 6.0 Hz, 2H), 3.31 (t, *J* = 6.0 Hz, 2H); ^{13}C NMR (150 MHz, CDCl$_3$): δ 198.4, 147.9, 139.7, 137.2, 135.0, 129.5, 128.9, 112.8, 108.1, 38.2, 36.5; HRMS (ESI): Exact mass calcd for $C_{14}H_{13}ClN_2O$ [M+H]$^+$, 261.0789; Found: 261.0794.

1-(4-bromophenyl)-3-(pyridin-2-ylamino)propan-1-one (1n)

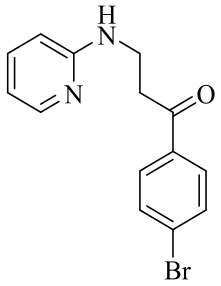

Yield: 74%. Mp 96-97 °C. White solid. ^{1}H NMR (300 MHz, CDCl$_3$): δ 8.10 (d, *J* = 5.1 Hz, 1H), 7.83 (d, *J* = 9.3 Hz, 2H), 7.61 (d, *J* = 9.0 Hz, 2H), 7.39 (t, *J* = 7.2 Hz, 1H), 6.59-6.55 (m, 1H), 6.40 (d, *J* = 7.5 Hz, 1H), 4.91 (br s, 1H), 3.82 (t, *J* = 6.3 Hz, 2H), 3.30 (t, *J* = 6.0 Hz, 2H); ^{13}C NMR (150 MHz, CDCl$_3$): δ 198.6, 158.2, 147.9, 137.2, 135.4, 131.9, 129.6, 128.5, 112.9, 108.1, 38.2, 36.5; HRMS (ESI): Exact mass calcd for $C_{14}H_{13}BrN_2O$ [M+H]$^+$, 305.0284; Found: 305.0289.

3-(pyridin-2-ylamino)-1-(thiophen-2-yl)propan-1-one (1o)

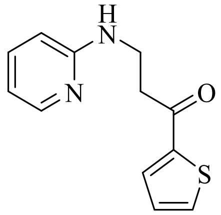

Yield: 62%. Mp 86-88°C. White solid. ^{1}H NMR (400 MHz, CDCl$_3$): δ 8.08 (d, *J* = 4.4 Hz, 1H), 7.71 (d, *J* = 3.6 Hz, 1H), 7.63 (d, *J* = 4.8 Hz, 1H), 7.36 (t, *J* = 7.2 Hz, 1H), 7.11 (t, *J* = 4.4 Hz, 1H), 6.55 (t, *J* = 6.0 Hz, 1H), 6.38 (d, *J* = 8.4 Hz, 1H), 4.91 (br s, 1H), 3.80 (t, *J* = 6.0 Hz, 2H), 3.26 (t, *J* = 6.0 Hz, 2H); ^{13}C NMR (75 MHz, CDCl$_3$): δ 192.2, 158.0, 147.7, 144.0, 137.0, 133.6, 132.0, 127.9, 112.6, 107.9, 53.2, 38.7, 36.7; HRMS (ESI): Exact mass calcd for $C_{12}H_{12}N_2OS$ [M+H]$^+$, 233.0743; Found: 233.0742.

1-(pyridin-2-ylamino)pentan-3-one (1p)

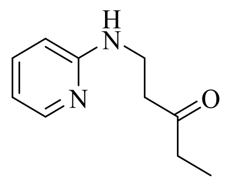

Yield: 75%. Mp 78-79 °C. White solid. ^1H NMR (300 MHz, $CDCl_3$): δ 8.09-8.07 (m, 1H), 7.38-7.35 (m, 1H), 6.57-6.53 (m, 1H), 6.36 (d, J = 7.5 Hz, 1H), 4.91 (br s, 1H), 3.66-3.59 (m, 2H), 2.77-2.73 (m, 2H), 2.48-2.41 (m, 2H), 1.09-1.03 (m, 3H); ^{13}C NMR (75 MHz, $CDCl_3$): δ 211.0, 158.4, 147.9, 137.2, 112.7, 107.7, 41.7, 36.4, 36.2, 7.6; HRMS (ESI): Exact mass calcd for $C_{10}H_{14}N_2O$ $[M+H]^+$, 179.1106; Found: 179.1110.

4,4-dimethyl-1-(pyridin-2-ylamino)pentan-3-one (1q)

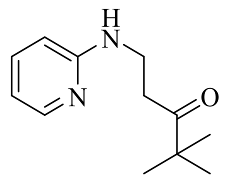

Yield: 43%. Colourless oil. ^1H NMR (300 MHz, $CDCl_3$): δ 8.08-8.05 (m, 1H), 7.39-7.33 (m, 1H), 6.55-6.51 (m, 1H), 6.35 (d, J = 8.4 Hz, 1H), 4.89 (br s, 1H), 3.60 (t, J = 6.0 Hz, 2H), 2.81 (t, J = 6.0 Hz, 2H), 1.11 (s, 9H); ^{13}C NMR (75 MHz, $CDCl_3$): δ 215.9, 158.3, 147.9, 137.2, 112.7, 107.7, 44.2, 36.6, 36.1, 26.2; HRMS (ESI): Exact mass calcd for $C_{12}H_{18}N_2O$ $[M+H]^+$, 207.1492; Found: 207.1494.

methyl 3-(pyridin-2-ylamino)propanoate (1r)

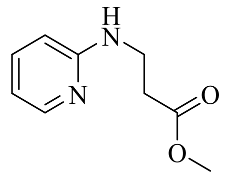

Yield: 56%. Mp 53-55 °C. White solid. ^1H NMR (300 MHz, $CDCl_3$): δ 8.08 (d, J = 3.9 Hz, 1H), 7.42-7.36 (m, 1H), 6.59-6.55 (m, 1H), 6.39 (d, J = 8.4 Hz, 1H), 4.94 (br s, 1H), 3.69-3.66 (m, 5H), 2.65 (t, J = 6.0 Hz, 2H); ^{13}C NMR (75 MHz, $CDCl_3$): δ 173.0, 158.2, 148.0, 137.3, 113.0, 107.7, 51.7, 37.3, 34.0; HRMS (ESI): Exact mass calcd for $C_9H_{12}N_2O_2$ $[M+H]^+$, 181.0977; Found: 181.0978.

phenyl-3-(quinolin-2-ylamino)propan-1-one (1s)

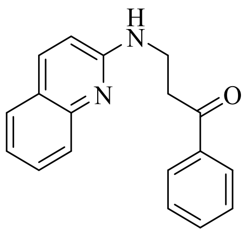

Yield: 77%. Mp 127-128 ºC. White solid. ^{1}H NMR (300 MHz, $CDCl_3$): δ 8.03 (d, *J* = 6.9 Hz, 2H), 7.80-7.72 (m, 2H), 7.59-7.48 (m, 5H), 7.29-7.23 (m, 1H), 6.61 (d, *J* = 8.7 Hz, 1H), 5.27 (br s, 1H), 4.04 (d, *J* = 5.7 Hz, 2H), 3.44 (t, *J* = 5.7 Hz, 2H); ^{13}C NMR (150 MHz, $CDCl_3$): δ 200.0, 156.4, 148.0, 137.1, 136.7, 133.3, 129.4, 128.6, 128.1, 127.4, 126.2, 123.4, 122.0, 112.4, 38.3, 36.3; HRMS (ESI): Exact mass calcd for $C_{18}H_{16}N_2O$ $[M+H]^+$, 277.1335; Found: 277.1339.

1-phenyl-3-(pyrimidin-2-ylamino)propan-1-one (1t)

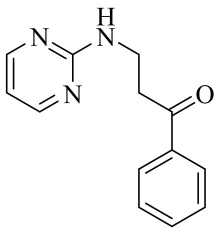

Yield: 68%. Mp 94-96 ºC. Pale yellow solid. ^{1}H NMR (600 MHz, $CDCl_3$): δ 8.25 (d, *J* = 4.8 Hz, 2H), 7.95 (d, *J* = 8.4 Hz, 2H), 7.56-7.53 (m, 1H), 7.45-7.43 (m, 2H), 6.51-6.50 (m, 1H), 5.66 (br s, 1H), 3.87 (t, *J* = 6.0 Hz, 2H), 3.31 (t, *J* = 6.0 Hz, 2H); ^{13}C NMR (150 MHz, $CDCl_3$): δ 199.2, 162.1, 158.1, 136.7, 133.2, 128.6, 128.0, 110.5, 38.2, 36.3; HRMS (ESI): Exact mass calcd for $C_{13}H_{13}N_3O$ $[M+H]^+$, 228.1132; Found: 228.1130.

phenyl-3-(pyridazin-3-ylamino)propan-1-one (1u)

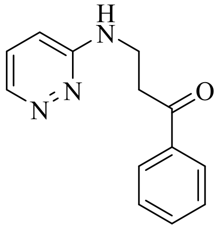

Yield: 45%. Mp 126-127 ºC. Pale yellow solid. ^{1}H NMR (300 MHz, $CDCl_3$): δ 8.54 (d, *J* = 4.5 Hz, 1H), 8.00-7.97 (m, 2H), 7.62-7.56 (m, 1H), 7.51-7.45 (m, 2H), 7.12 (t, *J* = 4.5 Hz, 1H), 6.63 (d, *J* = 9.0 Hz, 1H), 5.16 (br s, 1H), 4.04-3.98 (m, 2H), 3.42 (t, *J* =

5.7 Hz, 2H); ^{13}C NMR (75 MHz, $CDCl_3$): δ 199.8, 158.6, 143.6, 136.6, 133.5, 128.7, 128.1, 114.4, 37.7, 36.4; HRMS (ESI): Exact mass calcd for $C_{13}H_{13}N_3O$ $[M+H]^+$, 228.1131; Found: 228.1130.

3-(benzo[d]thiazol-2-ylamino)-1-phenylpropan-1-one (1v)

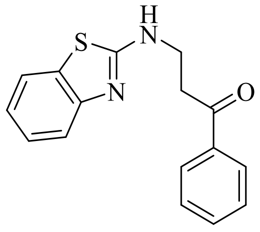

Yield: 51%. Mp 137-138 °C. White solid. ^1H NMR (300 MHz, $CDCl_3$): δ 8.01-7.97 (m, 2H), 7.63-7.56 (m, 3H), 7.51-7.45 (m, 2H), 7.33-7.28 (m, 1H), 7.09 (t, J = 7.5 Hz, 1H), 5.98 (br s, 1H), 3.96 (t, J = 5.7 Hz, 2H), 3.44 (t, J = 5.7 Hz, 2H); ^{13}C NMR (75 MHz, $CDCl_3$): δ 199.2, 166.7, 152.5, 136.4, 133.6, 130.5, 128.7, 128.1, 125.9, 121.7, 120.8, 118.9, 39.8, 37.9; HRMS (ESI): Exact mass calcd for $C_{16}H_{14}N_2OS$ $[M+H]^+$, 283.0900; Found:283.0899.

3. General procedure and product characterization

3.1 General procedure

Typical procedure for I_2-catalyzed intramolecular α-amination of carbonyl compounds to 3-acylimidazo[1,2-*a*]pyridines **2a**-**2v**.

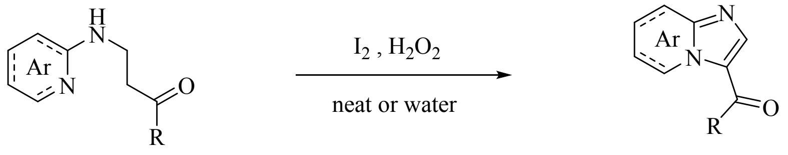

1a-1v **2a-2v**

Procedure: A reaction tube was equipped with a magnetic stir bar and successively charged with a mixture of **1a-1v** (0.2 mmol), I_2 (20 mol%) and H_2O_2 (0.4 mmol, 30 wt.% in H_2O) and was stirred at 80 °C in air for 0.5-6 h. [***Caution***: H_2O_2 is slightly unstable, so it should be kept at 2-8 °C; H_2O_2 also has certain dangerousness to the human, so, if it touches your skin or eyes, please splash it with warm water as soon as possible.] After the reaction was complete (as determined by TLC analysis), the reaction was cooled to room temperature and excess I_2 was quenched with a saturated aqueous solution of $Na_2S_2O_3$. Then EtOAc (20 mL) was added to the solution and

washed with brine, dried over Na_2SO_4, concentrated under reduced pressure. The crude product was purified by column chromatography on silica gel (petroleum ether/EtOAc = 2:1) to afford the targeted product **2a-2v**.

3.2 Product Characterization

imidazo[1,2-*a*]pyridin-3-yl(phenyl)methanone (2a)[1]

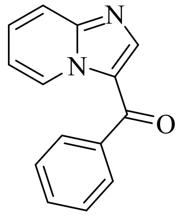

Yield: 88%. Mp 104-105 °C. White solid. ^{1}H NMR (400 MHz, $CDCl_3$): δ 9.75 (d, *J* = 6.8 Hz, 1H), 8.21 (s, 1H), 7.89-7.80 (m, 3H), 7.63-7.52 (m, 4H), 7.16 (t, *J* = 6.8 Hz, 1H); ^{13}C NMR (125 MHz, $CDCl_3$): δ 184.7, 149.0, 145.5, 139.2, 131.9, 129.3, 128.8, 128.7, 128.5, 123.5, 117.6, 115.0.

(6-methylimidazo[1,2-*a*]pyridin-3-yl)(phenyl)methanone (2b)

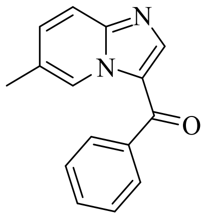

Yield: 81%. Mp 101-103 °C. White solid. ^{1}H NMR (300 MHz, $CDCl_3$): δ 9.58 (s, 1H), 8.17 (s, 1H), 7.88 (d, *J* = 8.1 Hz, 2H), 7.71 (d, *J* = 9.0 Hz, 1H), 7.64-7.51 (m, 3H), 7.42 (d, *J* = 9.0 Hz, 1H), 2.48 (s, 3H); ^{13}C NMR (75 MHz, $CDCl_3$): δ 184.8, 148.1, 145.6, 139.4, 132.4, 131.9, 128.8, 128.6, 126.9, 125.3, 123.3, 116.9, 18.4; HRMS (ESI): Exact mass calcd for $C_{15}H_{12}N_2O$ $[M+H]^+$, 237.1022; Found: 237.1030.

(6-fluoroimidazo[1,2-*a*]pyridin-3-yl)(phenyl)methanone (2c)

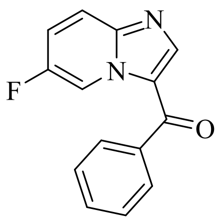

Yield: 74%. Mp 122-124 °C. White solid. ^{1}H NMR (300 MHz, $CDCl_3$): δ 9.79-9.77 (m, 1H), 8.26 (s, 1H), 7.92-7.88 (m, 2H), 7.84-7.78 (m, 1H), 7.67-7.46 (m, 4H); ^{13}C NMR (150 MHz, $CDCl_3$): δ 184.9, 155.4, 153.8, 145.9, 138.9, 132.3, 128.8, 128.7, 121.0, 120.8, 118.0, 117.9, 116.3, 116.0; HRMS (ESI): Exact mass calcd for $C_{14}H_9FN_2O$ $[M+H]^+$, 241.0772; Found: 241.0770.

(6-chloroimidazo[1,2-*a*]pyridin-3-yl)(phenyl)methanone (2d)

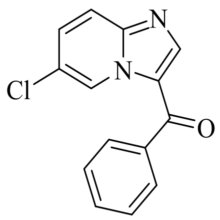

Yield: 67%. Mp 132-133 °C. White solid. ^{1}H NMR (300 MHz, $CDCl_3$): δ 9.86 (s, 1H), 8.23 (s, 1H), 7.88 (d, J = 7.8 Hz, 2H), 7.77 (d, J = 9.3 Hz, 1H), 7.67-7.52 (m, 4H); ^{13}C NMR (75 MHz, $CDCl_3$): δ 184.8, 145.6, 138.9, 132.3, 130.6, 128.8, 128.7, 126.9, 123.7, 123.6, 118.0; HRMS (ESI): Exact mass calcd for $C_{14}H_9ClN_2O$ $[M+H]^+$, 257.0476; Found: 257.0473.

(6-bromoimidazo[1,2-*a*]pyridin-3-yl)(phenyl)methanone (2e)[1]

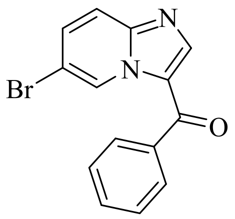

Yield: 82%. Mp 144-145 °C. White solid. ^{1}H NMR (300 MHz, $CDCl_3$): δ 9.96 (s, 1H), 8.21 (s, 1H), 7.89 (d, J = 6.0 Hz, 2H), 7.74-7.54 (m, 5H); ^{13}C NMR (150 MHz, $CDCl_3$): δ 184.8, 145.5, 138.8, 132.8, 132.3, 129.0, 128.8, 128.7, 118.2, 110.1.

phenyl(6-(trifluoromethyl)imidazo[1,2-*a*]pyridin-3-yl)methanone (2f)

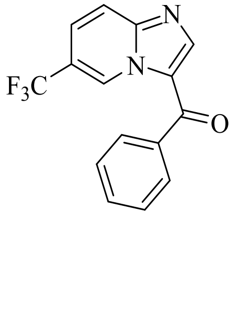

Yield: 48%. Mp 148-149 °C. White solid. ^{1}H NMR (300 MHz, $CDCl_3$): δ 10.16 (s, 1H), 8.33 (s, 1H), 7.96-7.90 (m, 3H), 7.73-7.64 (m, 2H), 7.61-7.55 (m, 2H); ^{13}C NMR (75 MHz, $CDCl_3$): δ 185.0, 148.8, 146.3, 138.6, 132.6, 128.9, 128.8, 127.9, 127.8, 125.2, 125.0, 124.2, 121.4, 119.7, 119.2, 118.5, 53.4; HRMS (ESI): Exact mass calcd for $C_{15}H_9F_3N_2O$ $[M+H]^+$, 291.0740; Found: 291.0740.

methyl 3-benzoylimidazo[1,2-*a*]pyridine-6-carboxylate (2g)

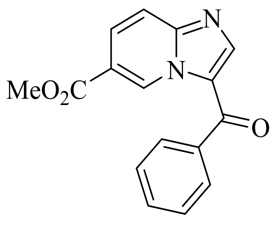

Yield: 67%. Mp 126-128 °C. White solid. ^{1}H NMR (300 MHz, $CDCl_3$): δ 10.43 (s, 1H), 8.30 (s, 1H), 8.13 (d, *J* = 9.3 Hz, 1H), 7.94-7.91 (m, 2H), 7.85 (d, *J* = 9.3 Hz, 1H), 7.67-7.55 (m, 3H), 4.03 (s, 3H); ^{13}C NMR (150 MHz, $CDCl_3$): δ 184.8, 164.9, 146.6, 138.7, 132.5, 132.4, 129.1, 128.9, 128.7, 119.1, 117.2, 52.7, 29.7; HRMS (ESI): Exact mass calcd for $C_{16}H_{12}N_2O_3$ $[M+H]^+$, 281.0921; Found: 281.0919.

(7-methylimidazo[1,2-*a*]pyridin-3-yl)(phenyl)methanone (2h)

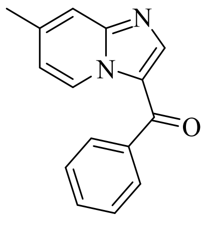

Yield: 93%. Mp 135-136 °C. Pale yellow solid. ^{1}H NMR (300 MHz, $CDCl_3$): δ 9.62 (d, *J* = 7.2 Hz, 1H), 8.16 (s, 1H), 7.89-7.86 (m, 2H), 7.64-7.51 (m, 4H), 7.00 (d, *J* = 5.4 Hz, 1H), 2.53 (s, 3H); ^{13}C NMR (75 MHz, $CDCl_3$): δ 184.5, 149.6, 146.0, 141.1, 139.4, 131.9, 128.8, 128.5, 128.0, 123.3, 117.6, 116.4, 21.6; HRMS (ESI): Exact mass calcd for $C_{15}H_{12}N_2O$ $[M+H]^+$, 237.1022; Found: 237.1033.

(7-methoxyimidazo[1,2-*a*]pyridin-3-yl)(phenyl)methanone (2i)

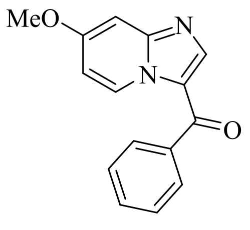

Yield: 82%. Mp 84-86 °C. White solid. ^{1}H NMR (300 MHz, $CDCl_3$): δ 9.56 (d, *J* = 7.5 Hz, 1H), 8.10 (s, 1H), 7.86 (d, *J* = 6.6 Hz, 2H), 7.63-7.50 (m, 3H), 7.07 (s, 1H), 6.82 (d, *J* = 7.5 Hz, 1H), 3.95 (s, 3H); ^{13}C NMR (75 MHz, $CDCl_3$): δ 184.2, 161.1, 151.4,

146.4, 139.4, 131.8, 129.4, 128.7, 128.5, 123.2, 109.1, 95.8, 55.8; HRMS (ESI): Exact mass calcd for $C_{15}H_{12}N_2O_2$ $[M+H]^+$, 253.0576; Found: 253.0580.

imidazo[1,2-*a*]pyridin-3-yl(m-tolyl)methanone (2j)

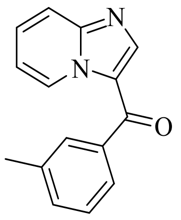

Yield: 91%. Mp 123-125 °C. White solid. ^{1}H NMR (400 MHz, $CDCl_3$): δ 9.75 (d, J = 6.8 Hz, 1H), 8.21 (s, 1H), 7.81 (d, J = 9.2 Hz, 1H), 7.68-7.66 (m, 2H), 7.57-7.53 (m, 1H), 7.42 (d, J = 4.8 Hz, 2H), 7.15 (t, J = 6.4 Hz, 1H), 2.46 (s, 3H); ^{13}C NMR (125 MHz, $CDCl_3$): δ 185.1, 149.1, 145.6, 139.3, 138.5, 132.8, 129.3, 128.9, 128.4, 126.0, 117.7, 115.0, 21.4; HRMS (ESI): Exact mass calcd for $C_{15}H_{12}N_2O$ $[M+H]^+$, 237.1022; Found: 237.1020.

imidazo[1,2-*a*]pyridin-3-yl(p-tolyl)methanone (2k)

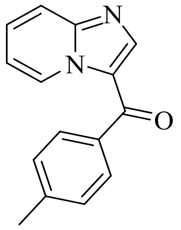

Yield: 89%. Mp 120-122 °C. White solid. ^{1}H NMR (400 MHz, $CDCl_3$): δ 9.73 (d, J = 6.8 Hz, 1H), 8.21 (s, 1H), 7.81 (t, J = 4.0 Hz, 3H), 7.56-7.52 (m, 1H), 7.34 (d, J = 8.0 Hz, 2H), 7.14 (t, J = 7.2 Hz, 1H), 2.47 (s, 3H); ^{13}C NMR (125 MHz, $CDCl_3$): δ 184.6, 148.9, 145.2, 142.6, 136.5, 129.1, 128.9, 128.8, 123.5, 117.6, 114.8, 53.3; HRMS (ESI): Exact mass calcd for $C_{15}H_{12}N_2O$ $[M+H]^+$, 237.1022; Found: 237.1021.

(4-fluorophenyl)(imidazo[1,2-*a*]pyridin-3-yl)methanone (2l)

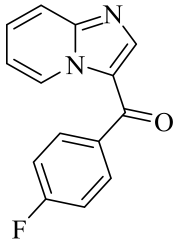

Yield: 93%. Mp 174-176 °C. White solid. ^{1}H NMR (300 MHz, $CDCl_3$): δ 9.74 (d, J = 6.9 Hz, 1H), 8.21 (s, 1H), 7.96-7.91 (m, 2H), 7.84 (d, J = 9.0 Hz, 1H), 7.61-7.56 (m, 1H), 7.28-7.16 (m, 3H); ^{13}C NMR (150 MHz, $CDCl_3$): δ 183.3, 181.0, 166.8, 163.5, 157.4, 149.2, 145.4, 135.5, 131.3, 131.2, 129.5, 128.9, 123.4, 117.8, 115.9, 115.6, 115.2, 94.0; HRMS (ESI): Exact mass calcd for $C_{14}H_9FN_2O$ $[M+H]^+$, 241.0771; Found:

241.0776.

(4-chlorophenyl)(imidazo[1,2-*a*]pyridin-3-yl)methanone (2m)

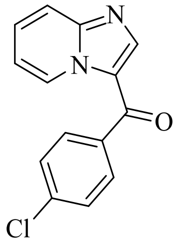

Yield: 80%. Mp 129-130 °C. White solid. ^{1}H NMR (300 MHz, $CDCl_3$): δ 9.74 (d, *J* = 6.9 Hz, 1H), 8.21 (s, 1H), 7.87-7.82 (m, 3H), 7.62-7.51 (m, 3H), 7.18 (t, *J* = 6.9 Hz, 1H); ^{13}C NMR (150 MHz, $CDCl_3$): δ 183.4, 149.2, 145.6, 138.4, 137.5, 130.2, 129.6, 128.9, 123.3, 117.8, 115.3, 110.0; HRMS (ESI): Exact mass calcd for $C_{14}H_9ClN_2O$ $[M+H]^+$, 257.0476; Found: 257.0471.

(4-bromophenyl)(imidazo[1,2-*a*]pyridin-3-yl)methanone (2n)

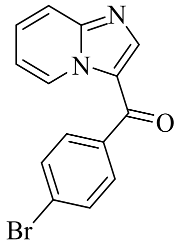

Yield: 93%. Mp 175-177 °C. White solid. ^{1}H NMR (300 MHz, $CDCl_3$): δ 9.73 (d, *J* = 7.2 Hz, 1H), 8.20 (s, 1H), 7.83 (d, *J* = 9.0 Hz, 1H), 7.78-7.67 (m, 4H), 7.62-7.56 (m, 1H), 7.19 (t, *J* = 6.9 Hz, 1H); ^{13}C NMR (150 MHz, $CDCl_3$): δ 183.5, 149.2, 148.6, 145.6, 138.0, 137.0, 131.9, 130.3, 129.7, 128.9, 126.9, 123.3, 122.3, 117.8, 115.3; HRMS (ESI): Exact mass calcd for $C_{14}H_9BrN_2O$ $[M+H]^+$, 300.9971; Found: 300.9970.

imidazo[1,2-*a*]pyridin-3-yl(thiophen-2-yl)methanone (2o)

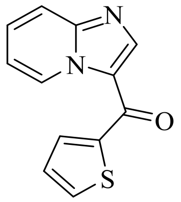

Yield: 83%. Mp 126-127 °C. White solid. ^{1}H NMR (600 MHz, $CDCl_3$): δ 9.63 (d, *J* = 7.2 Hz, 1H), 8.49 (s, 1H), 7.87 (t, *J* = 4.8 Hz, 1H), 7.79 (d, *J* = 9.0 Hz, 1H), 7.69 (t, *J* = 4.8 Hz, 1H), 7.54-7.51 (m, 1H), 7.25-7.20 (m, 1H), 7.11 (t, *J* = 7.2 Hz, 1H); ^{13}C NMR (150 MHz, $CDCl_3$): δ 175.7, 149.0, 143.9, 143.7, 132.6, 131.9, 131.8, 129.4, 129.3, 128.9, 128.7, 128.1, 127.9, 123.2, 117.8, 115.2, 114.9; HRMS (ESI): Exact mass calcd for $C_{12}H_8N_2OS$ $[M+H]^+$, 229.0430; Found: 229.0433.

1-(imidazo[1,2-*a*]pyridin-3-yl)propan-1-one (2p)

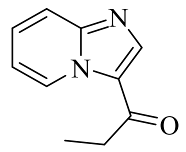

Yield: 56%. Mp 127-129 °C. White solid. ^{1}H NMR (300 MHz, $CDCl_3$): δ 9.69 (d, *J* = 6.9 Hz, 1H), 8.38 (s, 1H), 7.78 (d, *J* = 9.0 Hz, 1H), 7.53-7.48 (m, 1H), 7.10 (t, *J* = 6.9 Hz, 1H), 2.99 (t, *J* = 7.5 Hz, 2H), 1.31 (t, *J* = 7.5 Hz, 3H); ^{13}C NMR (150 MHz, $CDCl_3$): δ 191.1, 142.5, 128.9, 128.7, 117.6, 115.0, 32.6, 9.0; HRMS (ESI): Exact mass calcd for $C_{10}H_{10}N_2O$ [M+H], 175.0866; Found: 175.0869.

1-(imidazo[1,2-*a*]pyridin-3-yl)-2,2-dimethylpropan-1-one (2q)

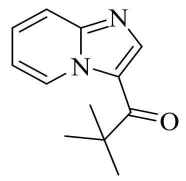

Yield: 97%. Mp 137-139 °C. White solid. ^{1}H NMR (300 MHz, $CDCl_3$): δ 9.80 (d, *J* = 6.9 Hz, 1H), 8.51 (s, 1H), 7.78 (d, *J* = 8.7 Hz, 1H), 7.50 (t, *J* = 7.5 Hz, 1H), 7.08 (t, *J* = 6.9 Hz, 1H), 1.48 (s, 9H); ^{13}C NMR (150 MHz, $CDCl_3$): δ 197.1, 142.5, 129.3, 128.7, 117.5, 114.8, 44.1, 28.7; HRMS (ESI): Exact mass calcd for $C_{12}H_{14}N_2O$ $[M+H]^+$, 203.1179; Found: 203.1178.

imidazo[1,2-*a*]quinolin-1-yl(phenyl)methanone (2s)

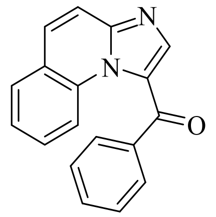

Yield: 90%. Mp 157-158 °C. White solid. ^{1}H NMR (300 MHz, $CDCl_3$): δ 8.46 (d, *J* = 8.7 Hz, 1H), 8.10 (d, *J* = 7.2 Hz, 2H), 8.01 (s, 1H), 7.91-7.84 (m, 2H), 7.73-7.65 (m, 3H), 7.62-7.54 (m, 3H); ^{13}C NMR (75 MHz, $CDCl_3$): δ 184.3, 149.3, 146.5, 138.6, 133.1, 131.5, 130.1, 129.1, 129.0, 128.6, 125.8, 124.7, 119.9, 116.8, 100.0; HRMS (ESI): Exact mass calcd for $C_{18}H_{12}N_2O$ $[M+H]^+$, 273.1022; Found: 273.1019.

imidazo[1,2-*a*]pyrimidin-3-yl(phenyl)methanone (2t)

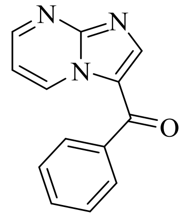

Yield: 74%. Mp 215-216 °C. White solid. ^{1}H NMR (300 MHz, $CDCl_3$): δ 10.01 (d, *J* =

6.9 Hz, 1H), 8.85 (d, J = 4.2 Hz, 1H), 8.43 (s, 1H), 7.94-7.90 (m, 2H), 7.70-7.55 (m, 3H), 7.28-7.23 (m, 1H); ^{13}C NMR (75 MHz, $CDCl_3$): δ 185.2, 153.7, 151.5, 146.4, 138.3, 136.7, 132.6, 128.9, 128.8, 121.8, 111.3; HRMS (ESI): Exact mass calcd for $C_{13}H_9N_3O$ $[M+H]^+$, 224.0818; Found: 224.0817.

imidazo[1,2-*b*]pyridazin-3-yl(phenyl)methanone (2u)

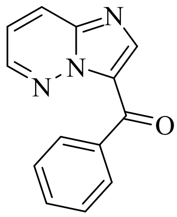

Yield: 83%. Mp 140-142 °C. White solid. ^{1}H NMR (300 MHz, $CDCl_3$): δ 8.69 (d, J = 4.5 Hz, 1H), 8.23 (s, 1H), 8.15 (d, J = 9.3 Hz, 1H), 7.96 (d, J = 6.9 Hz, 2H), 7.69-7.53 (m, 3H), 7.35 (t, J = 4.8 Hz, 1H); ^{13}C NMR (75 MHz, $CDCl_3$): δ 183.5, 144.6, 143.2, 142.7, 138.7, 132.7, 129.4, 128.6, 126.7, 126.2, 120.3; HRMS (ESI): Exact mass calcd for $C_{13}H_9N_3O$ $[M+H]^+$, 224.0818; Found: 224.0816.

benzo[d]imidazo[2,1-*b*]thiazol-3-yl(phenyl)methanone (2v)

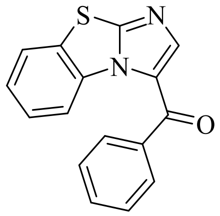

Yield: 82%. Mp 161-163 °C. White solid. ^{1}H NMR (300 MHz, $CDCl_3$): δ 8.97 (d, J = 8.7 Hz, 1H), 7.99-7.95 (m, 2H), 7.86 (s, 1H), 7.77 (d, J = 8.1 Hz, 1H), 7.69-7.63 (m, 1H), 7.59-7.52 (m, 3H), 7.45 (t, J = 7.8 Hz, 1H); ^{13}C NMR (150 MHz, $CDCl_3$): δ 183.5, 147.1, 138.5, 132.6, 129.3, 128.6, 126.6, 125.7, 123.7, 118.2; HRMS (ESI): Exact mass calcd for $C_{16}H_{10}N_2OS$ $[M+H]^+$, 279.0587; Found: 279.0591.

4. Diversification of 3-acylimidazo[1,2-a]pyridines

3-benzylimidazo[1,2-a]pyridine (3)[2]

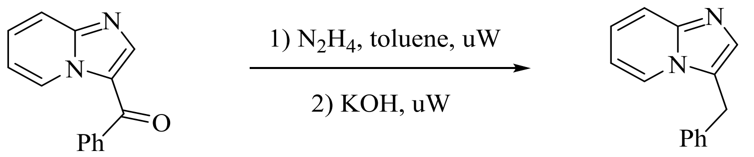

2a **3**

A mixture of **2a** (0.2 mmol) and 80% hydrazine hydrate (2 equiv.) in toluene (1 mL)

was taken in a flame-dried Schlenk tube and placed in a commercial microwave oven operating at 2450 MHz frequency. After irradiation of the mixture for 20 mins., (monitored by TLC) it was cooled to room temperature, extracted with chloroform and dried over anhydrous Na_2SO_4. Removal of solvent gave the hydrazone. Then a mixture of the obtained hydrazone and KOH (1.4 mmol) were taken in a flame-dried Schlenk tube and placed in a microwave oven. After the reaction was complete (as determined by TLC analysis), the reaction was cooled to room temperature and EtOAc (20 mL) was added to the solution and washed with brine, dried over Na_2SO_4, concentrated under reduced pressure. The crude product was purified by column chromatography on silica gel (petroleum ether/EtOAc = 2:1) to afford the targeted product **3**. Yield: 77%. White semisolid. ^{1}H NMR (300 MHz, $CDCl_3$): δ 7.78 (d, J = 6.9 Hz, 1H), 7.64 (d, J = 9.0 Hz, 1H), 7.48 (s, 1H), 7.32-7.26 (m, 3H), 7.22-7.16 (m, 3H), 6.74 (t, J = 6.9 Hz, 1H), 4.27 (s, 2H); ^{13}C NMR (75 MHz, $CDCl_3$): δ 145.8, 136.6, 132.6, 128.8, 128.3, 126.9, 123.6, 123.2, 122.5, 117.9, 112.1, 30.3; HRMS (ESI): Exact mass calcd for $C_{14}H_{12}N_2$ $[M+H]^+$, 209.1079; Found: 209.1082.

2-bromo-1-(imidazo[1,2-a]pyridin-3-yl)propan-1-one (4)[3]

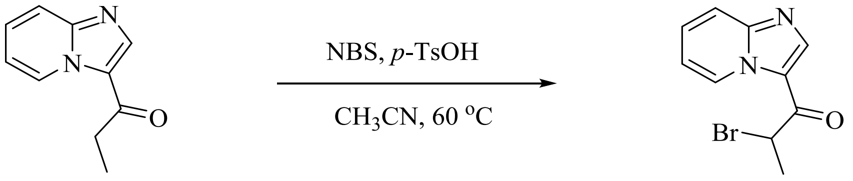

2p **4**

N-bromosuccinimide (NBS, 1.2 equiv.) and and *p*-toluenesulfonic acid ($T_SOH.H_2O$, 0.2 equiv.) was added to a solution of **2p** (0.2 mmol) in anhydrous CH_3CN (1 mL) at room temperature. After the addition, the reaction mixture was warmed to 60 ^{o}C and stirred for 4 h. After the reaction was complete (as determined by TLC analysis), the reaction was cooled to room temperature and EtOAc (20 mL) was added to the solution and washed succesively with H_2O, saturated $NaHCO_3$ solution, brine, dried over Na_2SO_4, concentrated under reduced pressure. The crude product was purified by column chromatography on silica gel (petroleum ether/EtOAc = 2:1) to afford the targeted product **4**. Yield: 86%. Mp 185-187 ^{o}C. White solid. 1H NMR (300 MHz, $CDCl_3$): δ 9.65 (d, J = 6.9 Hz, 1H), 8.50 (s, 1H), 7.82 (d, J = 9.0 Hz, 1H), 7.57 (t, J = 6.9 Hz, 1H), 7.17 (t, J = 6.9 Hz, 1H), 5.24 (t, J = 6.9 Hz, 1H), 1.96 (d, J = 6.6 Hz, 3H); 13C NMR (75 MHz, $CDCl_3$): δ 183.7, 149.5, 143.6, 129.8, 129.0, 121.1, 117.9, 115.6, 42.9, 20.5; HRMS (ESI): Exact mass calcd for $C_{10}H_9BrN_2O$ $[M+H]^+$, 252.9977; Found: 252.9974.

1-(imidazo[1,2-a]pyridin-3-yl)-2-phenylpropan-1-one (5)[4]

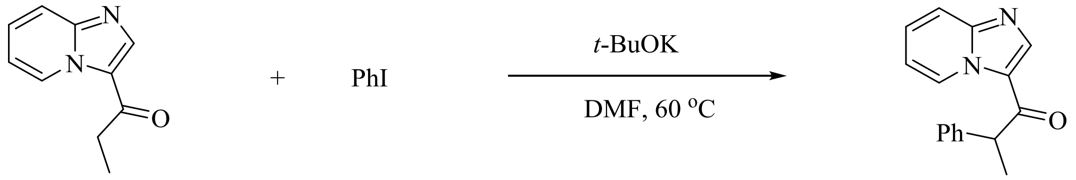

2p **5**

To a flame-dried Schlenk flask were added KOtBu (5.0 equiv.) and **2p** (2.0 equiv.). The flask was evacuated and backfilled with argon 3 times, then iodobenzene (0.1 mmol) was dissolved in dry DMF (1 mL) then was added by syringe, and the mixture was allowed to stir for 10 minutes at room temperature. The reaction mixture was stirred and heated at 60 ^{o}C for 13 h. After allowing the reaction to cool to room temperature, 1N HCl (2 mL) was added and the mixture was allowed to stir for 10 minutes at room temperature. After the reaction was complete (as determined by TLC analysis), the reaction was cooled to room temperature and EtOAc (20 mL) was added to the solution and washed with brine, dried over Na_2SO_4, concentrated under reduced pressure. The crude product was purified by column chromatography on silica gel (petroleum ether/EtOAc = 2:1) to afford the targeted product **5**. Yield: 54%. Mp 169-171 ^{o}C. White solid. 1H NMR (300 MHz, $CDCl_3$): δ 9.70 (d, *J* = 6.9 Hz, 1H), 8.40 (s, 1H), 7.74 (d, *J* = 9.0 Hz, 1H), 7.49 (t, *J* = 7.8 Hz, 1H), 7.41 (d, *J* = 7.5 Hz, 2H), 7.36-7.21 (m, 3H), 7.08 (t, *J* = 6.6 Hz, 1H), 4.58 (t, *J* = 6.9 Hz, 1H), 1.63 (d, *J* = 6.9 Hz, 3H); 13C NMR (75 MHz, $CDCl_3$): δ 190.6, 148.8, 143.3, 141.6, 138.6, 129.1, 128.9, 127.6, 127.1, 123.2, 117.7, 115.0, 49.1, 18.7; HRMS (ESI): Exact mass calcd for $C_{16}H_{14}N_2O$ $[M+H]^+$, 251.1184; Found: 251.1187.

1-(imidazo[1,2-a]pyridin-3-yl)-2-methylprop-2-en-1-one (6)[5]

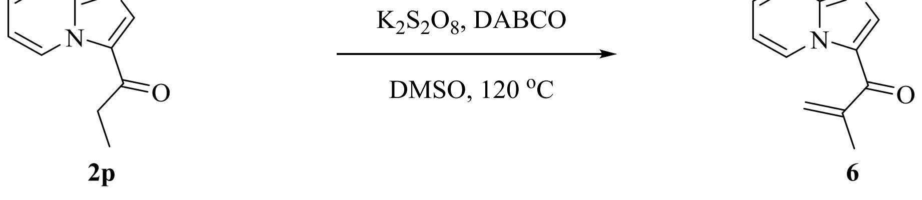

2p **6**

In a Schlenk tube of 25 mL, DABCO (0.5 equiv.) and **2p** (0.2 mmol) were dissolved in DMSO (1.0 mL) and stirred at room temperature for 1 minutes. Then $K_2S_2O_8$ (2 equiv.) were added. The mixture was stirred at 120 ºC for 20 h under Ar atmosphere. After the reaction was complete (as determined by TLC analysis), the reaction was cooled to

room temperature and EtOAc (20 mL) was added to the solution and washed with brine, dried over Na_2SO_4, concentrated under reduced pressure. The crude product was purified by column chromatography on silica gel (petroleum ether/EtOAc = 2:1) to afford the targeted product **6**. Yield: 74%. Mp 165-167 °C. Pale yellow solid. 1H NMR (300 MHz, $CDCl_3$): δ 9.63 (d, *J* = 6.9 Hz, 1H), 8.28 (s, 1H), 7.79 (d, *J* = 9.0 Hz, 1H), 7.53 (t, *J* = 7.8 Hz, 1H), 7.12 (t, *J* = 6.9 Hz, 1H), 5.77 (s, 2H), 2.13 (s, 3H); 13C NMR (75 MHz, $CDCl_3$): δ 186.7, 149.3, 144.9, 129.2, 128.8, 123.1, 122.7, 117.7, 115.0, 18.8; HRMS (ESI): Exact mass calcd for $C_{11}H_{10}N_2O$ $[M+H]^+$, 187.0871; Found: 187.0868.

1-(imidazo[1,2-a]pyridin-3-yl)-2-morpholinopropan-1-one (7)[3]

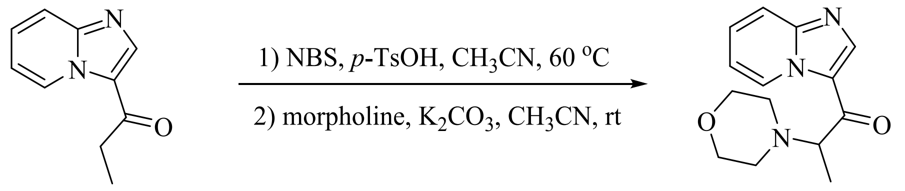

2p **7**

N-bromosuccinimide (NBS, 1.2 equiv.) and and *p*-toluenesulfonic acid ($T_SOH.H_2O$, 0.2 equiv.) was added to a solution of **2p** (0.2 mmol) in anhydrous CH_3CN (1 mL) at room temperature. After the addition, the reaction mixture was warmed to 60 °C and stirred for 4 h. After the reaction was complete (as determined by TLC analysis), the reaction was cooled to room temperature and EtOAc (20 mL) was added to the solution and washed succesively with H_2O, saturated $NaHCO_3$ solution, brine, dried over Na_2SO_4, concentrated under reduced pressure to give **4**, which was used for the next step without purifucation. Then to the mixture of K_2CO_3 (2.5 equiv.) and morpholine (3.0 equiv.) in CH_3CN (0.5 mL), **4** in CH_3CN (0.3 mL) was added slowly. After the addition, the reaction mixture was stirred until the reaction was completed at room temperature. EtOAc (20 mL) was added to the solution and washed with brine, dried over Na_2SO_4, concentrated under reduced pressure. The crude product was purified by column chromatography on silica gel (petroleum ether/EtOAc = 2:1) to afford the targeted product **7**. Yield: 72%. Mp 178-180 °C. White solid. 1H NMR (300 MHz, $CDCl_3$): δ 9.70 (d, *J* = 6.9 Hz, 1H), 8.76 (s, 1H), 7.78 (d, *J* = 9.0 Hz, 1H), 7.51 (t, *J* = 6.9 Hz, 1H), 7.10 (t, *J* = 6.9 Hz, 1H), 3.78 (t, *J* = 6.6 Hz, 1H), 3.74-3.70 (m, 4H), 2.72-2.65 (m, 2H), 2.61-2.54 (m, 2H), 1.38 (d, *J* = 6.9 Hz, 3H); 13C NMR (75 MHz, $CDCl_3$): δ 191.2, 148.7, 143.9, 129.3, 128.9, 122.8, 117.7, 115.2, 67.4, 67.1, 50.7, 13.7; HRMS (ESI): Exact mass calcd for $C_{14}H_{17}N_3O_2$ $[M+H]^+$, 260.1399; Found: 260.1393.

1-(imidazo[1,2-a]pyridin-3-yl)-2-iodopropan-1-one (8)[6]

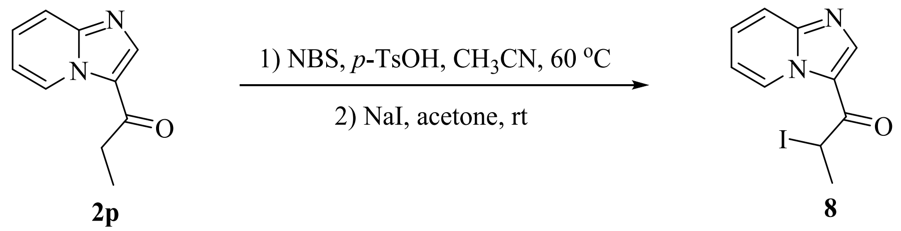

N-bromosuccinimide (NBS, 1.2 equiv.) and and *p*-toluenesulfonic acid ($T_SOH.H_2O$, 0.2 equiv.) was added to a solution of **2p** (0.2 mmol) in anhydrous CH_3CN (1 mL) at room temperature. After the addition, the reaction mixture was warmed to 60 °C and stirred for 4 h. After the reaction was complete (as determined by TLC analysis), the reaction was cooled to room temperature and EtOAc (20 mL) was added to the solution and washed succesively with H_2O, saturated $NaHCO_3$ solution, brine, dried over Na_2SO_4, concentrated under reduced pressure to give **4**, which was used for the next step without purifucation. A solution of sodium iodide (1.1 equiv.) in anhydrous acetone (0.5 mL) was added to a solution of **4** in the same solvent (0.5 mL). The formation of sodium bromide precipitate is observed instantly. The reaction was stirred at rt for 10 min and, then, filtered. Removal of the solvent under reduced pressure afforded the expected product **8**. No further purification was needed. Yield: 76%. Mp 195-197 °C. White solid. 1H NMR (300 MHz, $CDCl_3$): δ 9.63 (d, *J* = 6.9 Hz, 1H), 8.47 (s, 1H), 7.80 (d, *J* = 7.5 Hz, 1H), 7.59-7.50 (m, 1H), 7.18-7.12 (m, 1H), 5.45 (t, *J* = 6.9 Hz, 1H), 2.10 (d, *J* = 6.9 Hz, 3H); 13C NMR (75 MHz, $CDCl_3$): δ 185.3, 149.4, 143.6, 143.1, 129.8, 129.6, 129.0, 120.1, 117.9, 115.6, 115.5, 43.0, 18.7; HRMS (ESI): Exact mass calcd for $C_{10}H_9IN_2O$ $[M+H]^+$, 300.9769; Found: 300.9765.

5. Contral Experiments

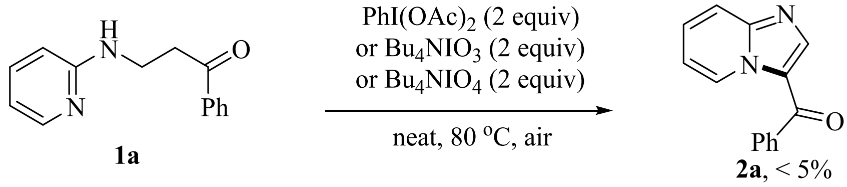

(1)

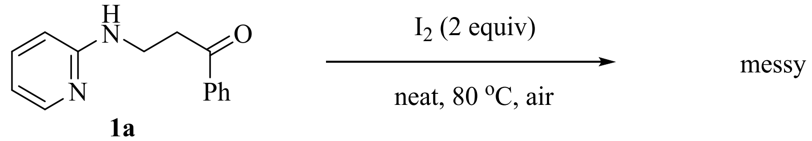

(2)

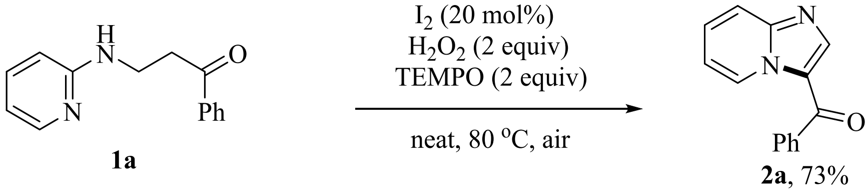

(3)

6. References

1. (a) N. Chernyak, V. Gevorgyan, *Angew. Chem., Int. Ed.,* 2010, **49**, 2743; (b) H. G. Wang, Y. Wang, D. D. Liang, L. Y. Liu, J. C. Zhang, Q. Zhu, *Angew. Chem., Int. Ed.,* 2011, **50**, 5677; (c) L. J. Ma, X. P. Wang, W. Yu, B. Han, *Chem. Commun.,* 2011, **47**, 11333; (d) C. He, J. Hao, H. Xu, Y. Mo, H. Liu, J. Han, A. Lei, *Chem. Commun.,* 2012, **48**, 11073; (e) D. Chandra Mohan, S. N. Rao, S. Adimurthy, *J. Org. Chem.,* 2013, **78**, 1266; (f) H. Cao, X. Liu, J. Liao, J. Huang, H. Qiu, Q. Chen, Y. Chen, *J. Org. Chem.,* 2014, **79**, 11209; (g) H. Zhan, L. Zhao, J. Liao, N. Li, Q. Chen, S. Qiu, H. Cao, *Adv. Synth. Catal.,* 2015, **357**, 46; (h) K. R. Reddy, A. P. Gupta, P. Das, *Asian J. Org. Chem.,* 2016, **5**, 900.
2. S. Gadhwal, M. Baruah, J. S. Sandhu, *Synlett*, 1999, **10**, 1573.
3. W. Wu, C. You, Y. Liu, X.-Q. Dong, X. Zhang, *Org. Lett.*, 2017, **19**, 2548.
4. M. Pichette Drapeau, I. Fabre, L. Grimaud, I. Ciofini, T. Ollevier, M. Taillefer, *Angew. Chem., Int. Ed.*, 2015, **54**, 10587.
5. Y. Liu, X. Zhan, P. Ji, J. Xu, Q. Liu, W. Luo, T. Chen, C. Guo, *Chem. Commun.*, 2017, **53**, 5346.
6. V. Estévez, V. Sridharan, S. Sabaté, M. Villacampa, J. C. Menéndez, *Asian J. Org. Chem.*, 2016, **5**, 652.

7. Copies of 1H NMR and 13C NMR Spectra

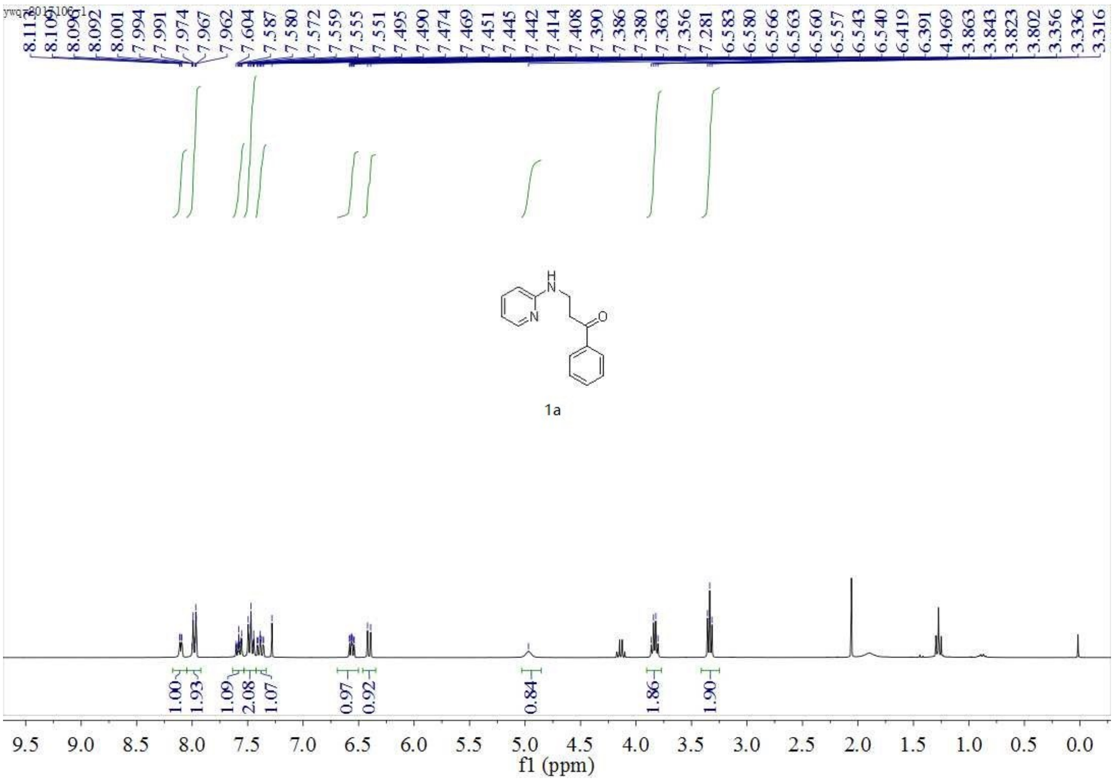

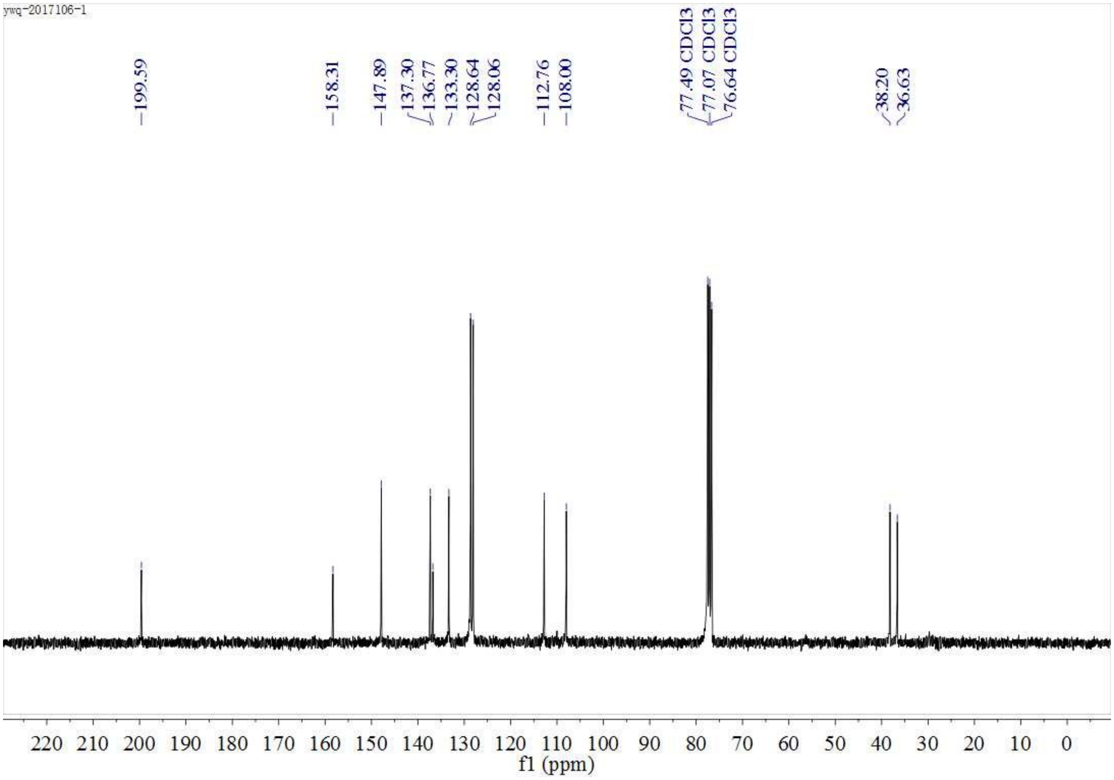

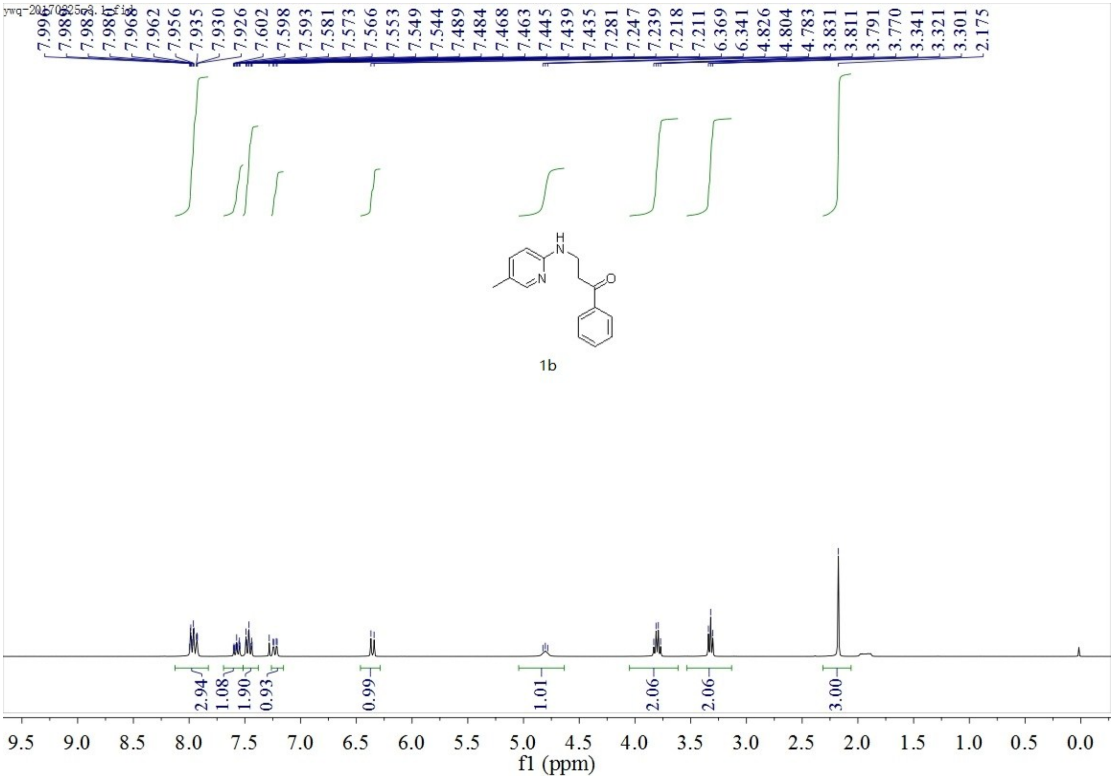

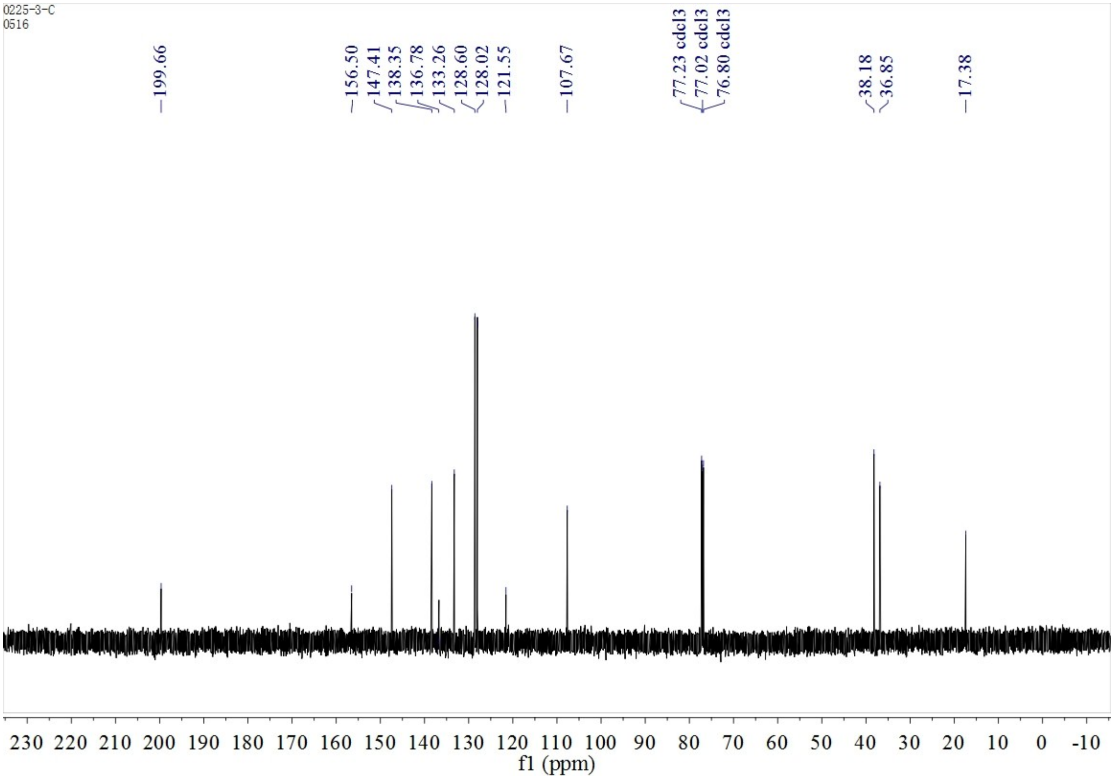

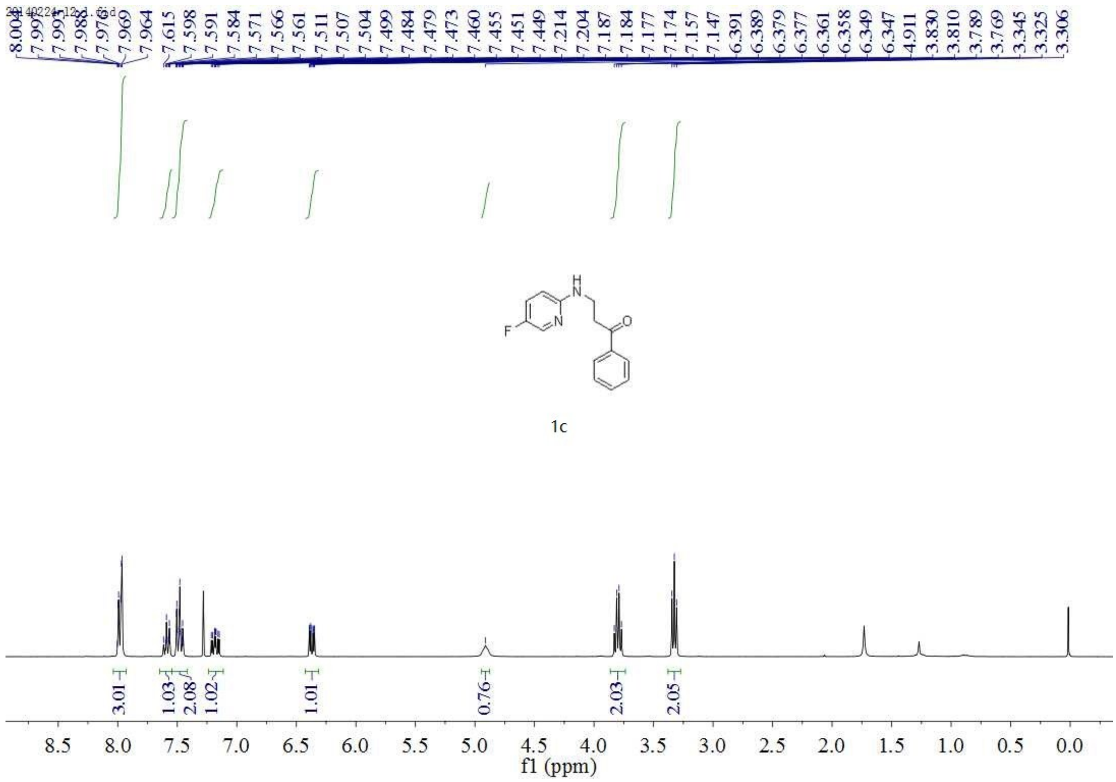
8.004
7.969
7.964
7.615
7.598
7.591
7.584
7.571
7.566
7.561
7.511
7.507
7.504
7.499
7.484
7.479
7.473
7.460
7.455
7.451
7.449
7.214
7.204
7.187
7.184
7.177
7.174
7.157
7.147
6.391
6.389
6.379
6.377
6.361
6.358
6.349
6.347
4.911
3.830
3.810
3.789
3.769
3.345
3.325
3.306
3.01
1.03
2.08
1.02
1.01
0.76
2.03
2.05
8.5 8.0 7.5 7.0 6.5 6.0 5.5 5.0 4.5 4.0 3.5 3.0 2.5 2.0 1.5 1.0 0.5 0.0
f1 (ppm)

1c

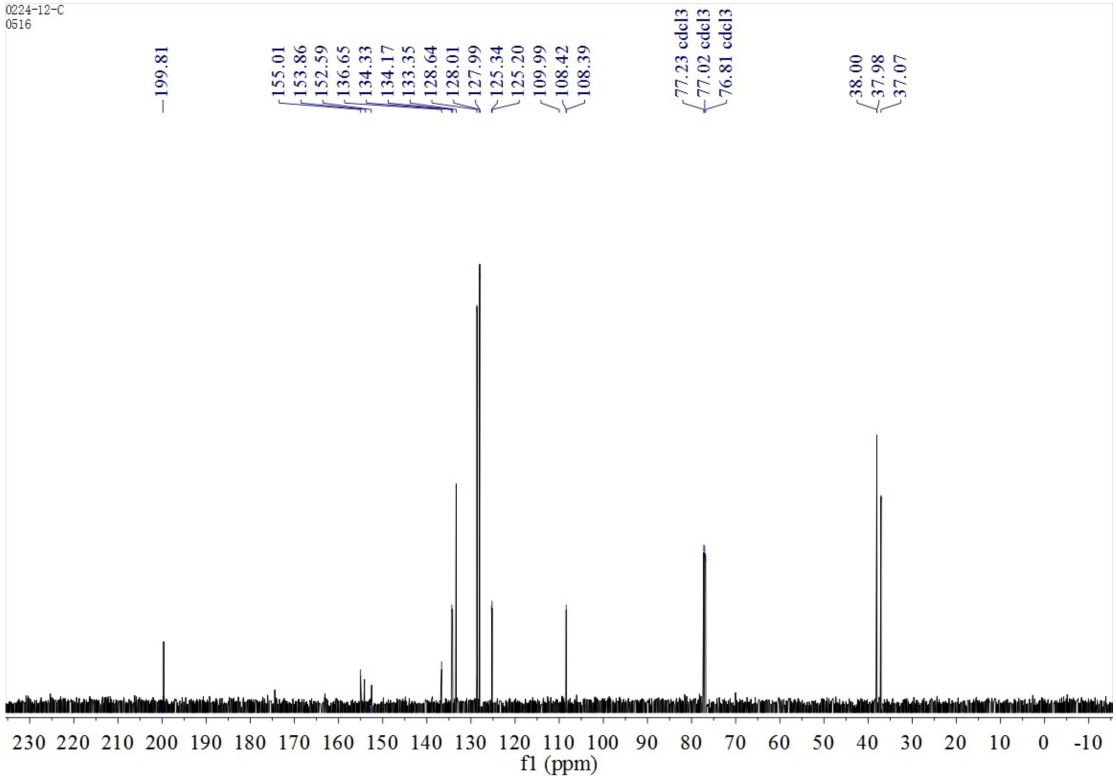
0224-12-C
0516
199.81
155.01
153.86
152.59
136.65
134.33
134.17
133.35
128.64
128.01
127.99
125.34
125.20
109.99
108.42
108.39
77.23 cdcl3
77.02 cdcl3
76.81 cdcl3
38.00
37.98
37.07
230 220 210 200 190 180 170 160 150 140 130 120 110 100 90 80 70 60 50 40 30 20 10 0 -10
f1 (ppm)

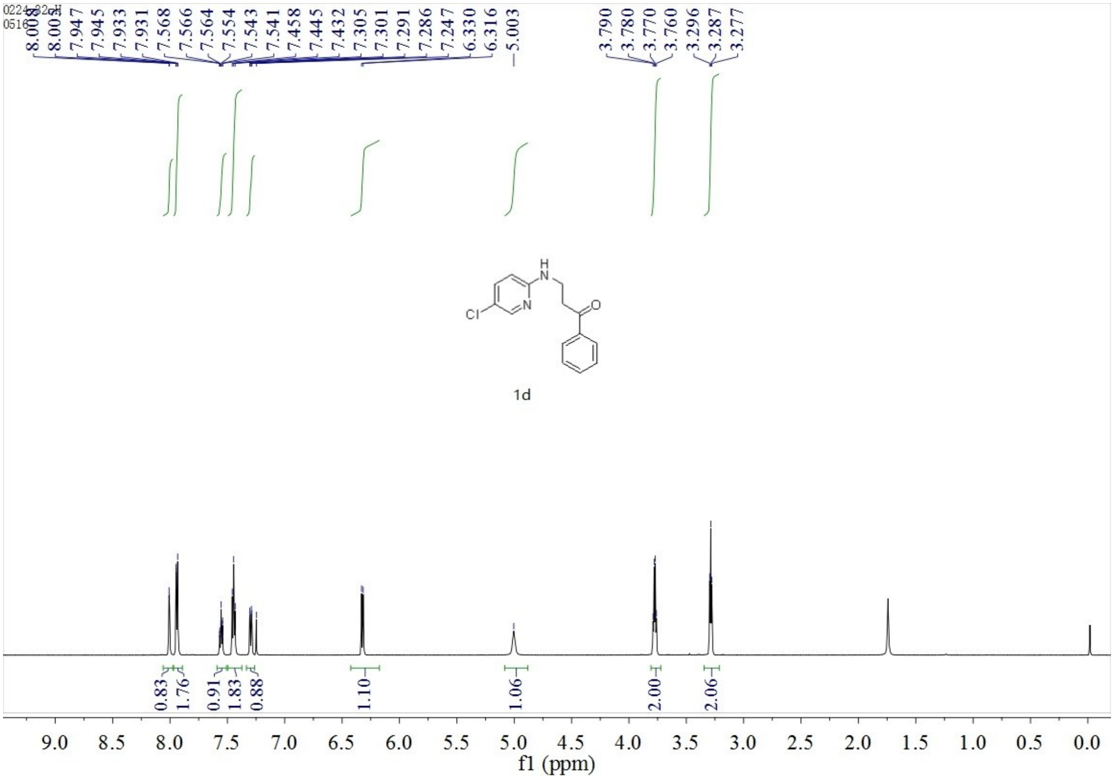
0224-32-H
0516
1d
f1 (ppm)

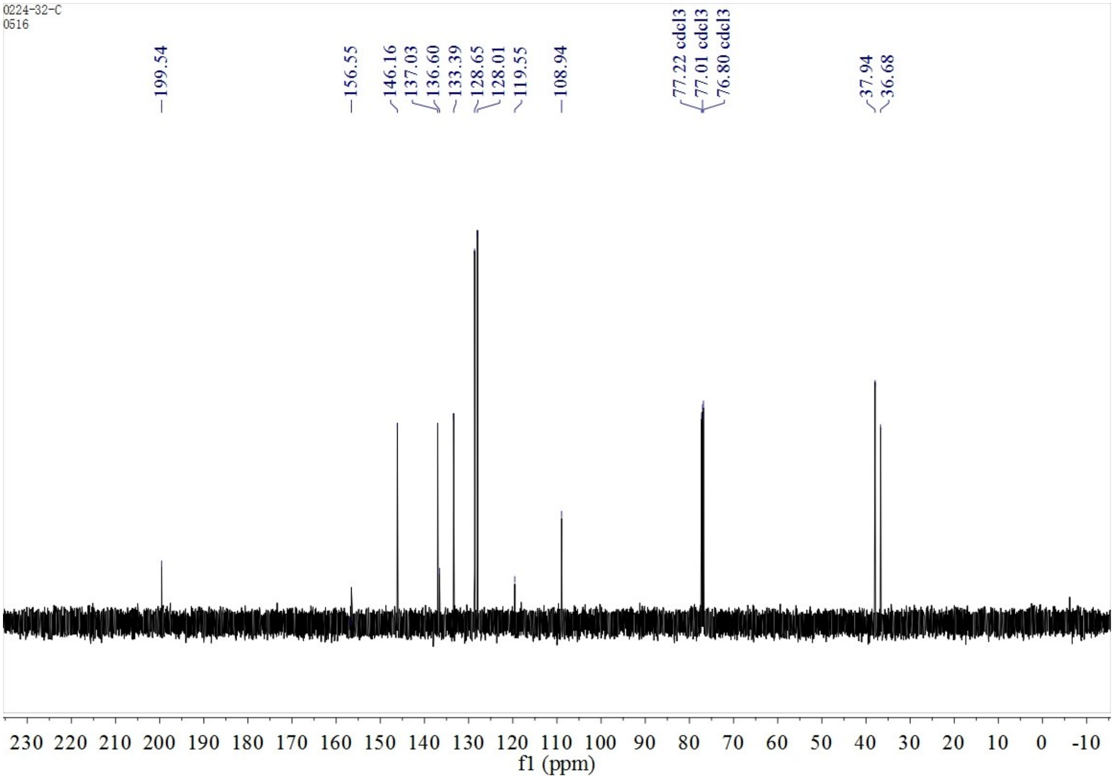
0224-32-C
0516
199.54
156.55
146.16
137.03
136.60
133.39
128.65
128.01
119.55
108.94
77.22 cdcl3
77.01 cdcl3
76.80 cdcl3
37.94
36.68
f1 (ppm)

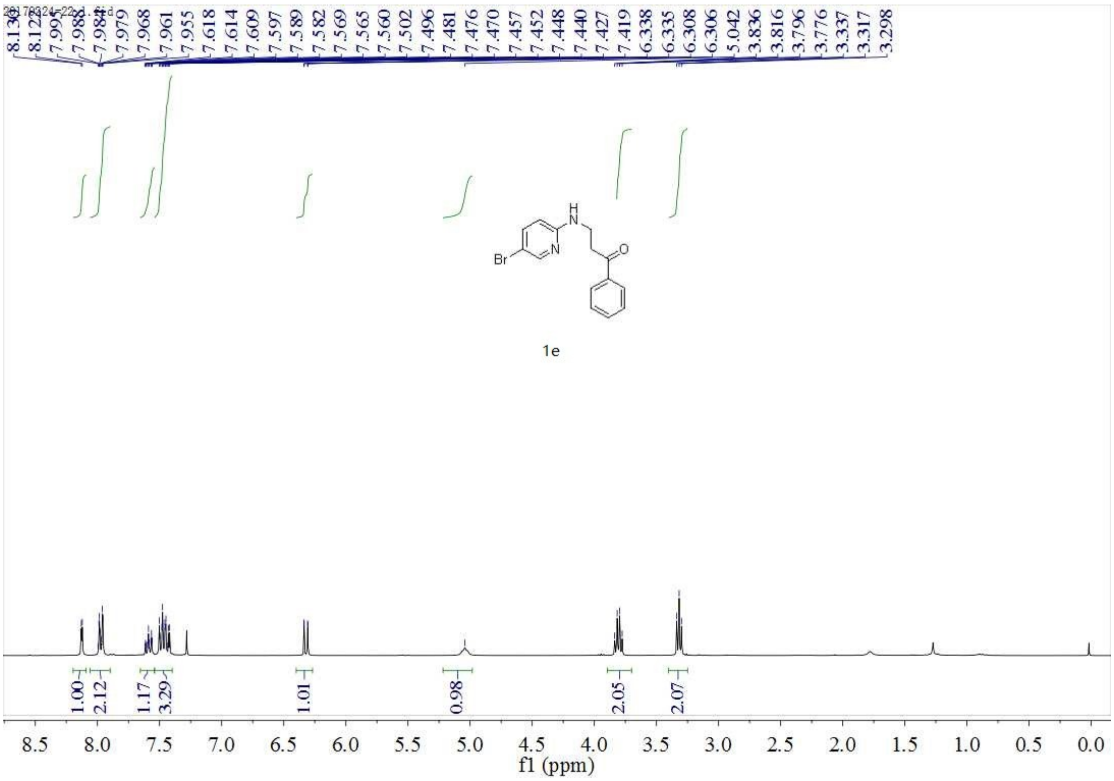
8.130
8.125
7.995
7.988
7.984
7.979
7.968
7.961
7.955
7.618
7.614
7.609
7.597
7.589
7.582
7.569
7.565
7.560
7.502
7.496
7.481
7.476
7.470
7.457
7.452
7.448
7.440
7.427
7.419
6.338
6.335
6.308
6.306
5.042
3.836
3.816
3.796
3.776
3.337
3.317
3.298
Br
N
H
O
1e
1.00
2.12
1.17
3.29
1.01
0.98
2.05
2.07
8.5 8.0 7.5 7.0 6.5 6.0 5.5 5.0 4.5 4.0 3.5 3.0 2.5 2.0 1.5 1.0 0.5 0.0
f1 (ppm)

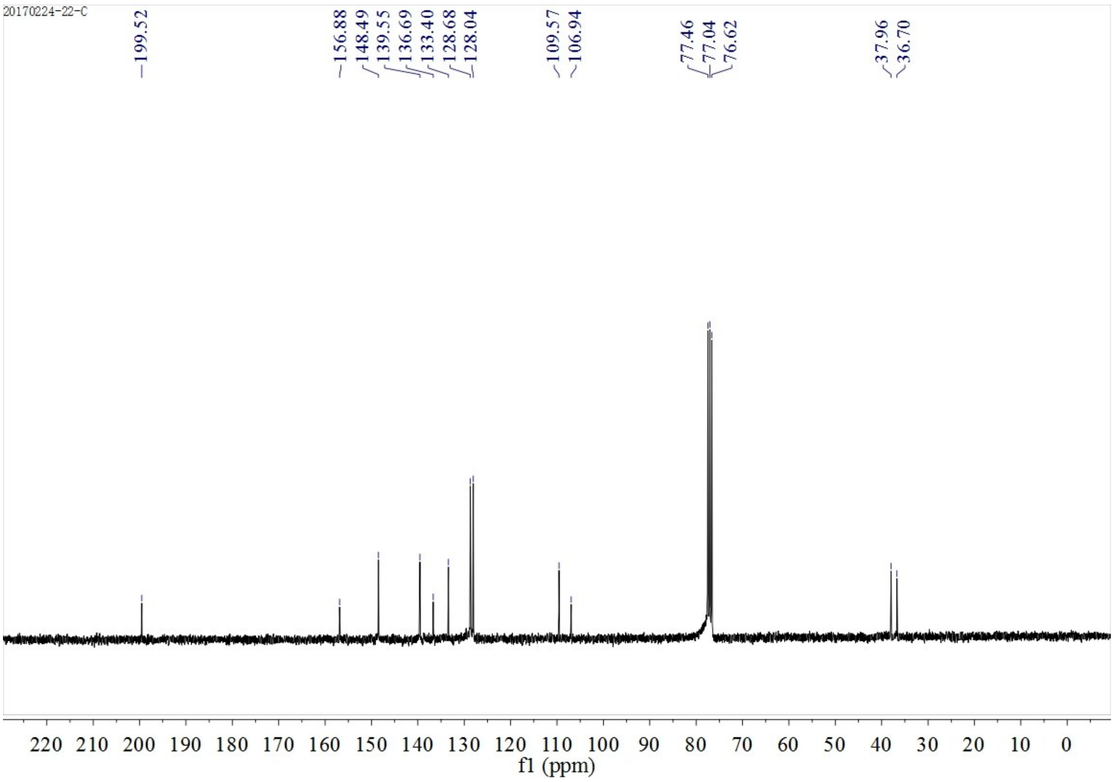
20170224-22-C
199.52
156.88
148.49
139.55
136.69
133.40
128.68
128.04
109.57
106.94
77.46
77.04
76.62
37.96
36.70
220 210 200 190 180 170 160 150 140 130 120 110 100 90 80 70 60 50 40 30 20 10 0
f1 (ppm)

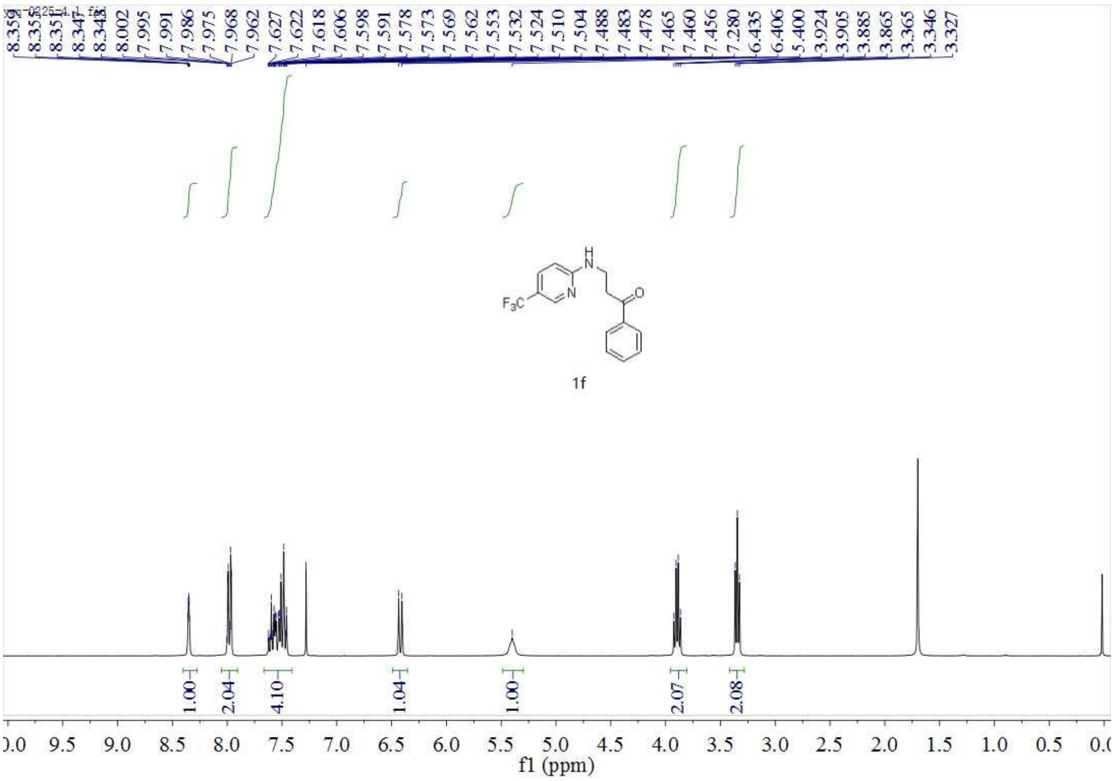

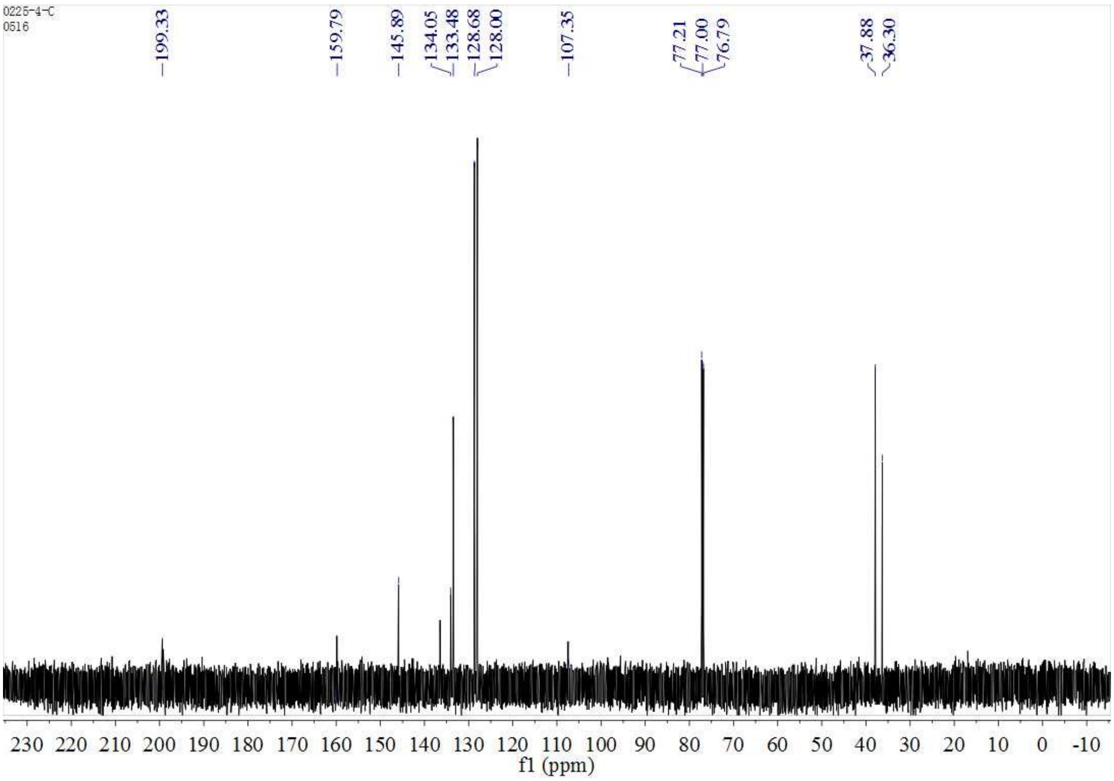

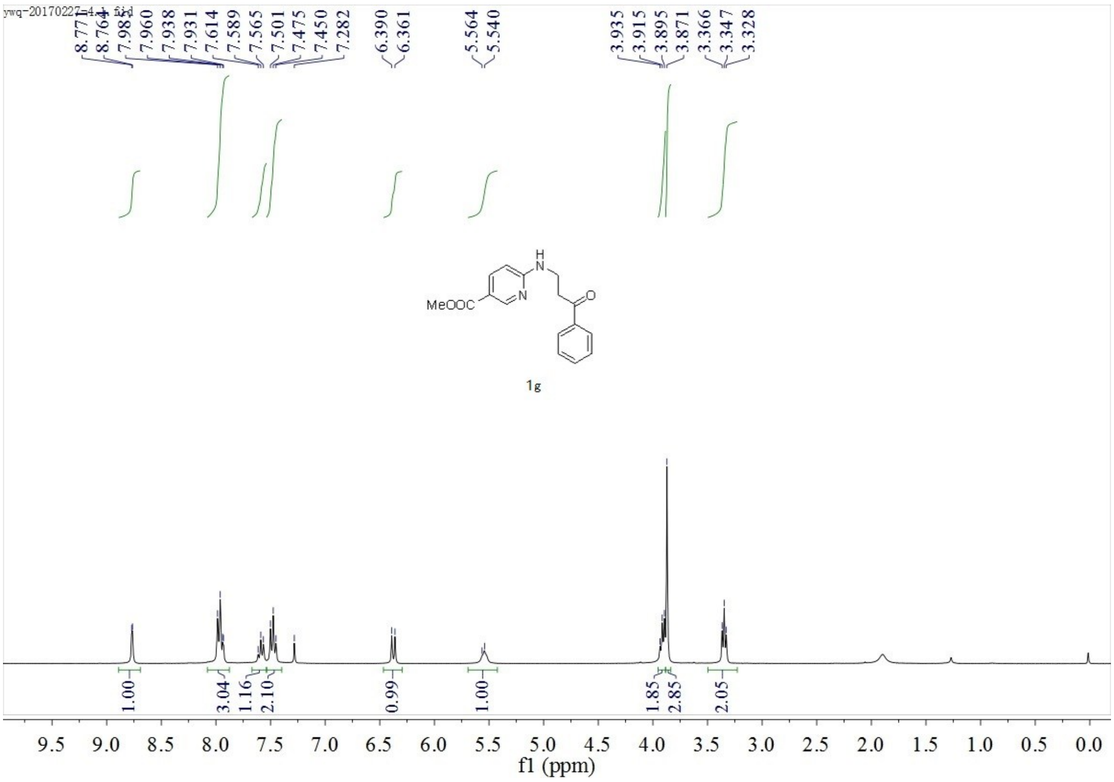
8.764
7.960
7.938
7.931
7.614
7.589
7.565
7.501
7.475
7.450
7.282
6.390
6.361
5.564
5.540
3.935
3.915
3.895
3.871
3.366
3.347
3.328
MeOOC
N
H
O
1g
1.00
3.04
1.16
2.10
0.99
1.00
1.85
2.85
2.05
f1 (ppm)

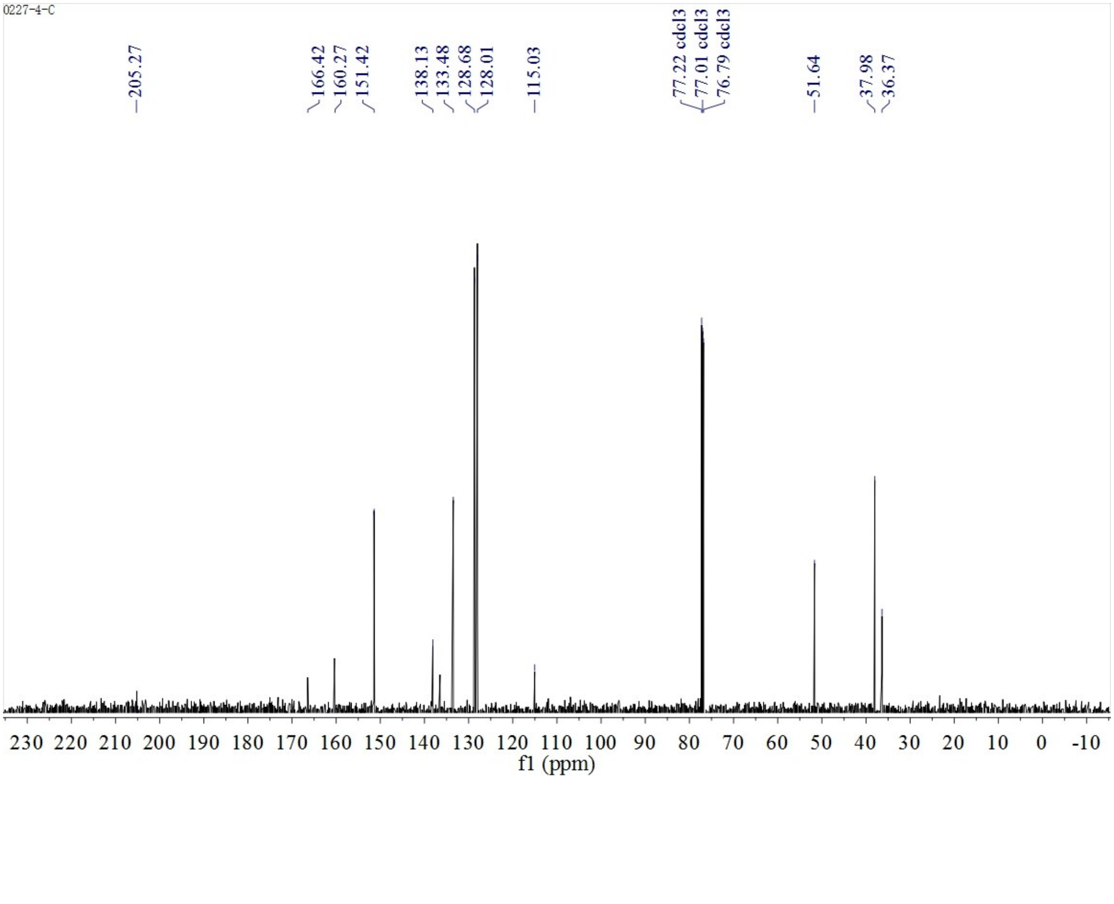
0227-4-C
205.27
166.42
160.27
151.42
138.13
133.48
128.68
128.01
115.03
77.22 cdcl3
77.01 cdcl3
76.79 cdcl3
51.64
37.98
36.37
f1 (ppm)

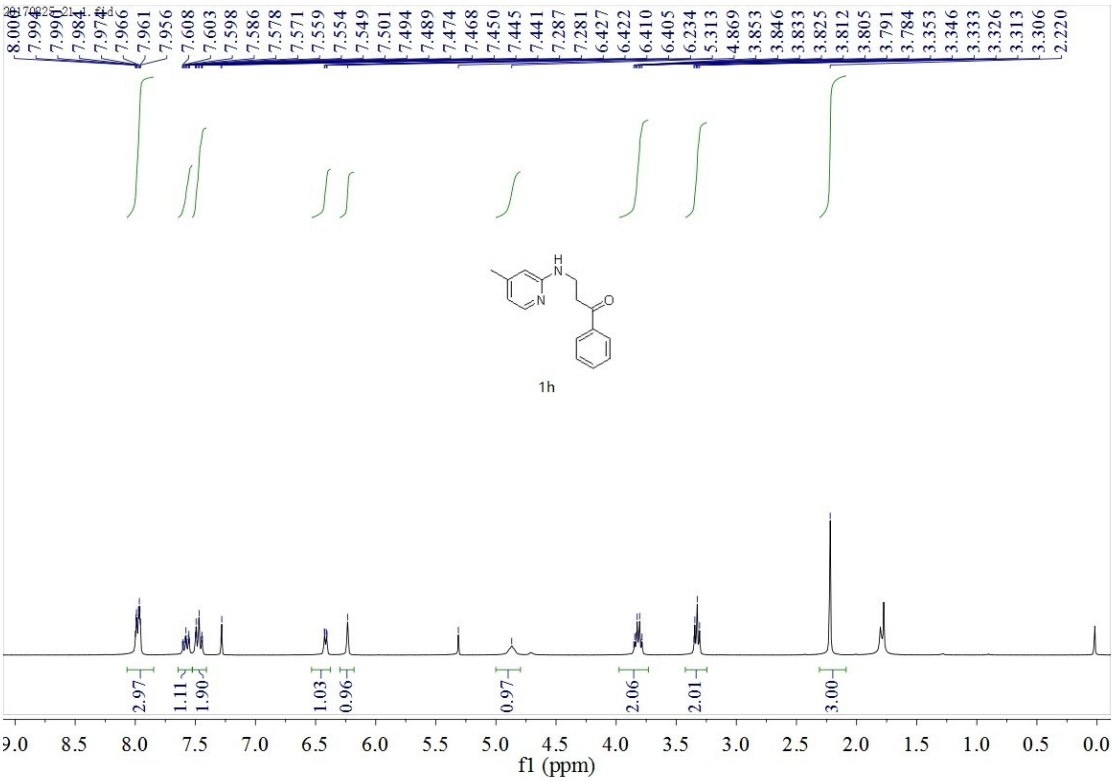

8.001
7.994
7.990
7.984
7.974
7.966
7.961
7.956
7.608
7.603
7.598
7.586
7.578
7.571
7.559
7.554
7.549
7.501
7.494
7.489
7.474
7.468
7.450
7.445
7.441
7.287
7.281
6.427
6.422
6.410
6.405
6.234
5.313
4.869
3.853
3.846
3.833
3.825
3.812
3.805
3.791
3.784
3.353
3.346
3.333
3.326
3.313
3.306
2.220
1h
2.97
1.11
1.90
1.03
0.96
0.97
2.06
2.01
3.00
9.0 8.5 8.0 7.5 7.0 6.5 6.0 5.5 5.0 4.5 4.0 3.5 3.0 2.5 2.0 1.5 1.0 0.5 0.0
f1 (ppm)

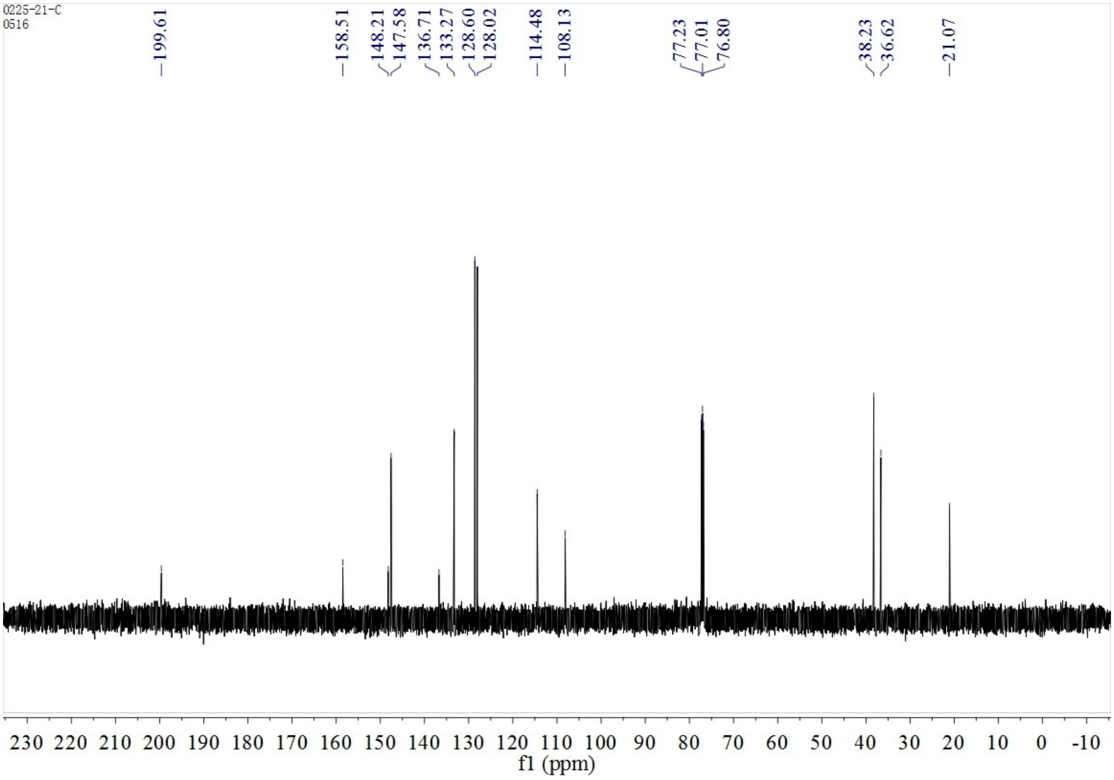

0225-21-C
0516
199.61
158.51
148.21
147.58
136.71
133.27
128.60
128.02
114.48
108.13
77.23
77.01
76.80
38.23
36.62
21.07
230 220 210 200 190 180 170 160 150 140 130 120 110 100 90 80 70 60 50 40 30 20 10 0 -10
f1 (ppm)

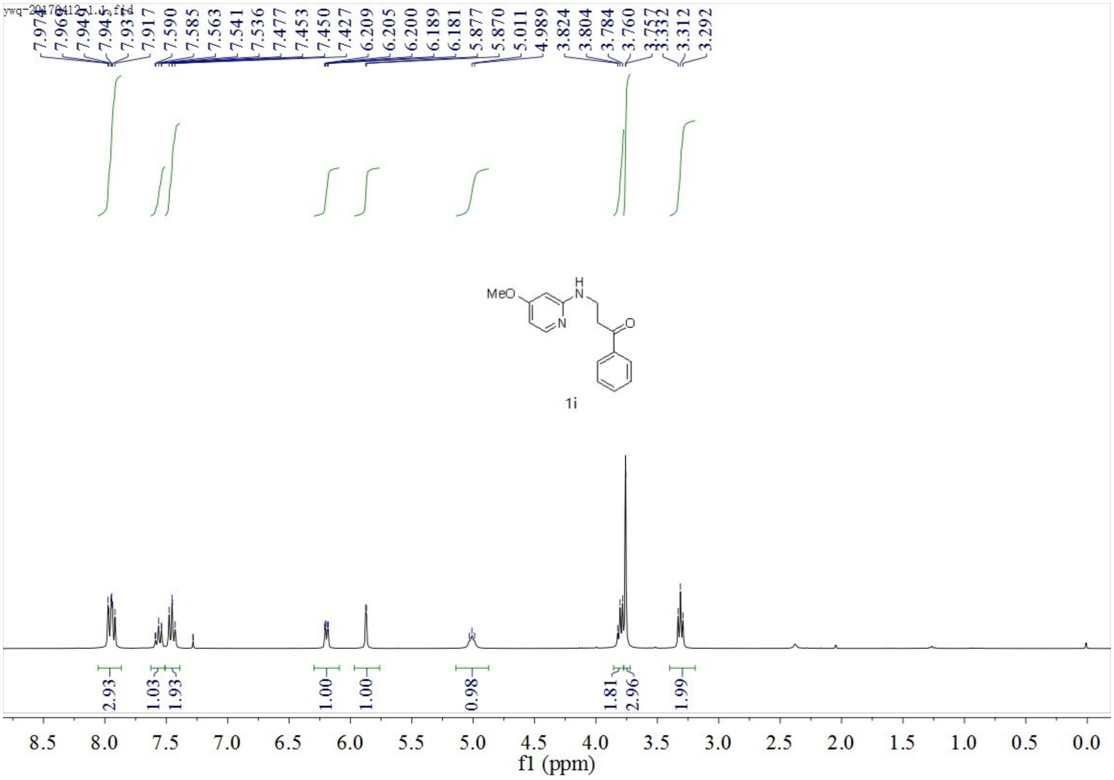
7.917
7.590
7.585
7.563
7.541
7.536
7.477
7.453
7.450
7.427
6.209
6.205
6.200
6.189
6.181
5.877
5.870
5.011
4.989
3.824
3.804
3.784
3.760
3.757
3.332
3.312
3.292
MeO
N
H
O
1i
2.93
1.03
1.93
1.00
1.00
0.98
1.81
2.96
1.99
f1 (ppm)

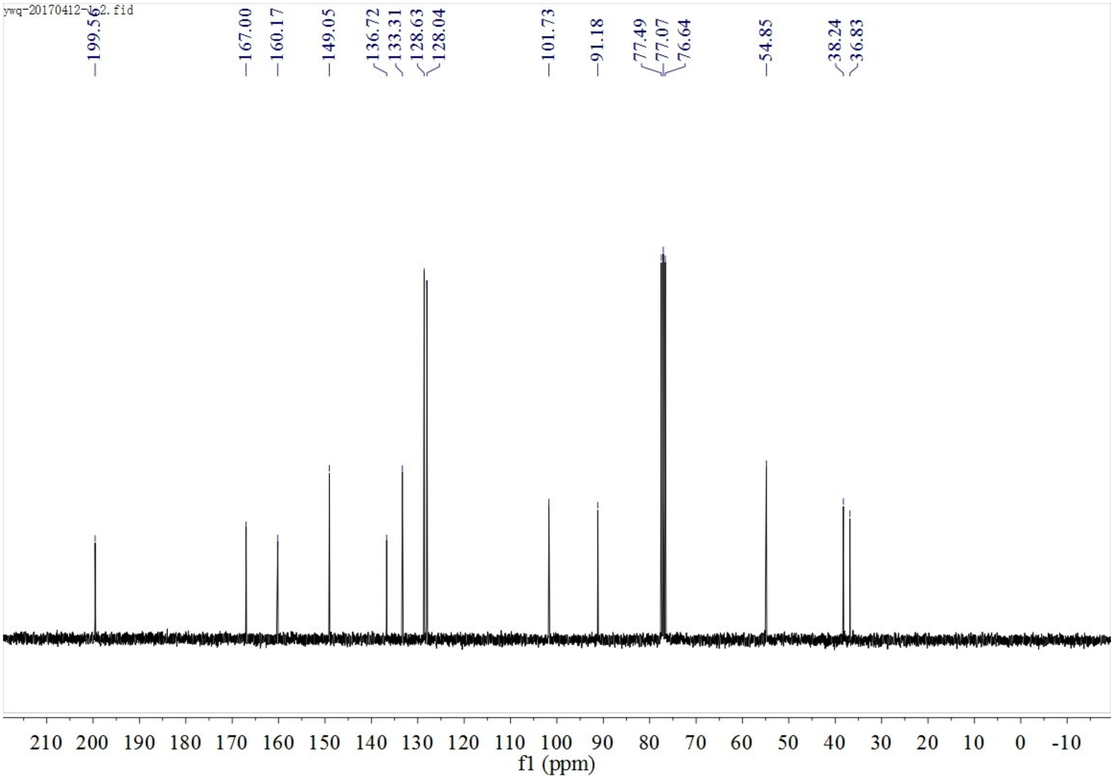
ywq-20170412-1-2.fid
167.00
160.17
149.05
136.72
133.31
128.63
128.04
101.73
91.18
77.49
77.07
76.64
54.85
38.24
36.83
f1 (ppm)

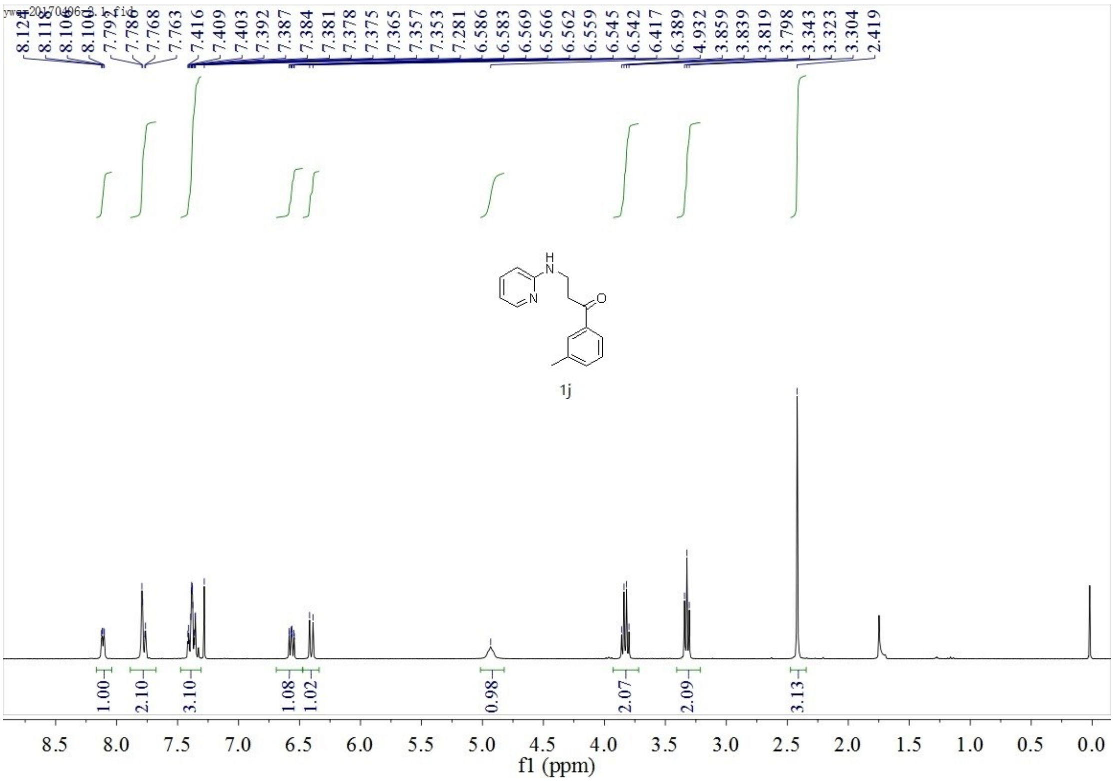
8.124
8.118
8.106
8.100
7.791
7.786
7.768
7.763
7.416
7.409
7.403
7.392
7.387
7.384
7.381
7.378
7.375
7.365
7.357
7.353
7.281
6.586
6.583
6.569
6.566
6.562
6.559
6.545
6.542
6.417
6.389
4.932
3.859
3.839
3.819
3.798
3.343
3.323
3.304
2.419
H
N
O
1j
1.00
2.10
3.10
1.08
1.02
0.98
2.07
2.09
3.13
8.5 8.0 7.5 7.0 6.5 6.0 5.5 5.0 4.5 4.0 3.5 3.0 2.5 2.0 1.5 1.0 0.5 0.0
f1 (ppm)

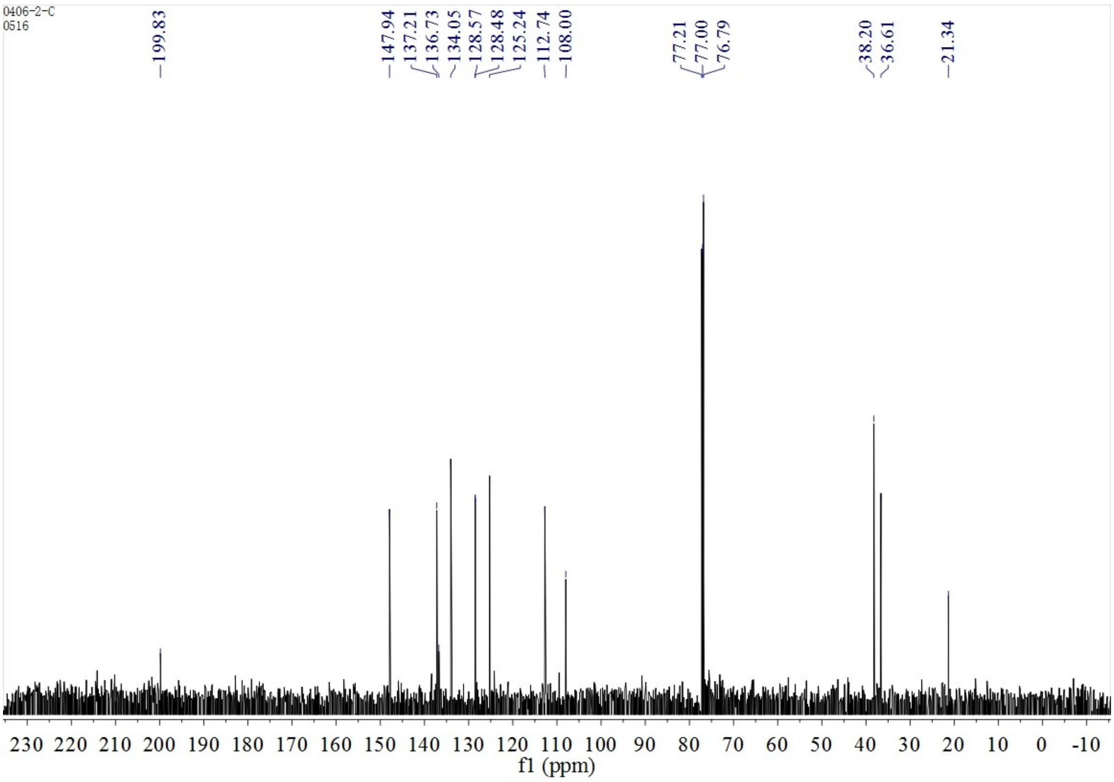
0406-2-C
0516
199.83
147.94
137.21
136.73
134.05
128.57
128.48
125.24
112.74
108.00
77.21
77.00
76.79
38.20
36.61
21.34
230 220 210 200 190 180 170 160 150 140 130 120 110 100 90 80 70 60 50 40 30 20 10 0 -10
f1 (ppm)

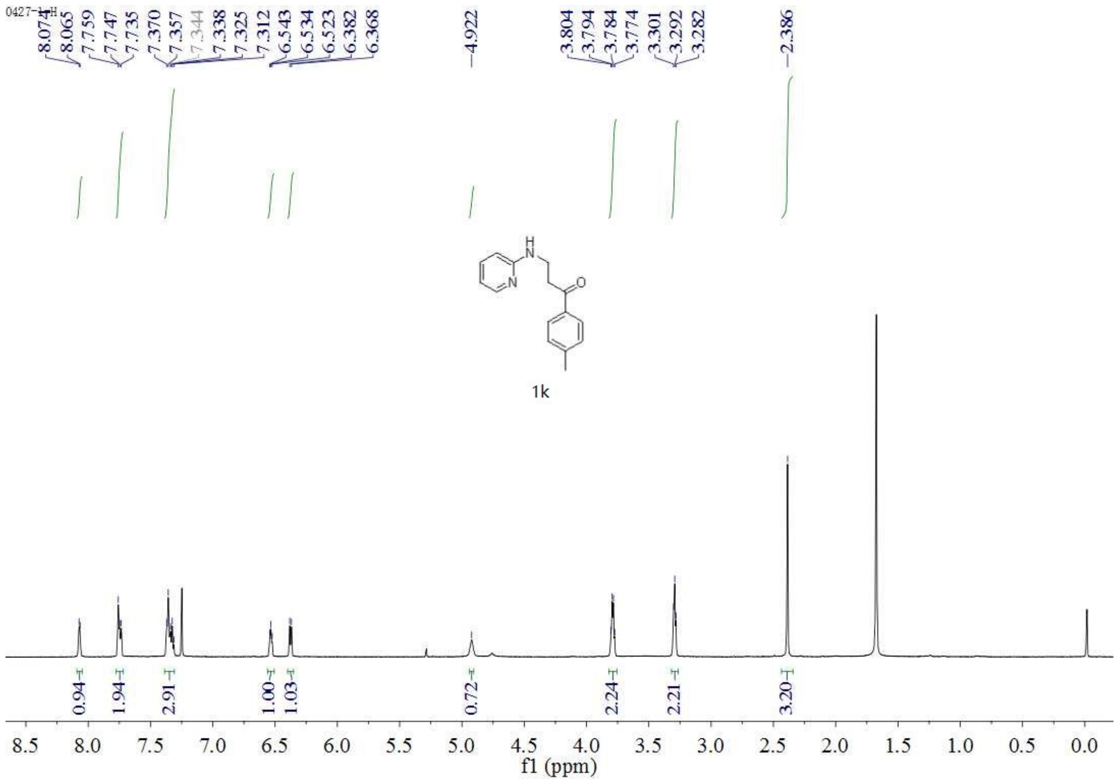
0427-1-H
8.074
8.065
7.759
7.747
7.735
7.370
7.357
7.344
7.338
7.325
7.312
6.543
6.534
6.523
6.382
6.368
4.922
3.804
3.794
3.784
3.774
3.301
3.292
3.282
2.386
1k
0.94
1.94
2.91
1.00
1.03
0.72
2.24
2.21
3.20
8.5 8.0 7.5 7.0 6.5 6.0 5.5 5.0 4.5 4.0 3.5 3.0 2.5 2.0 1.5 1.0 0.5 0.0
f1 (ppm)

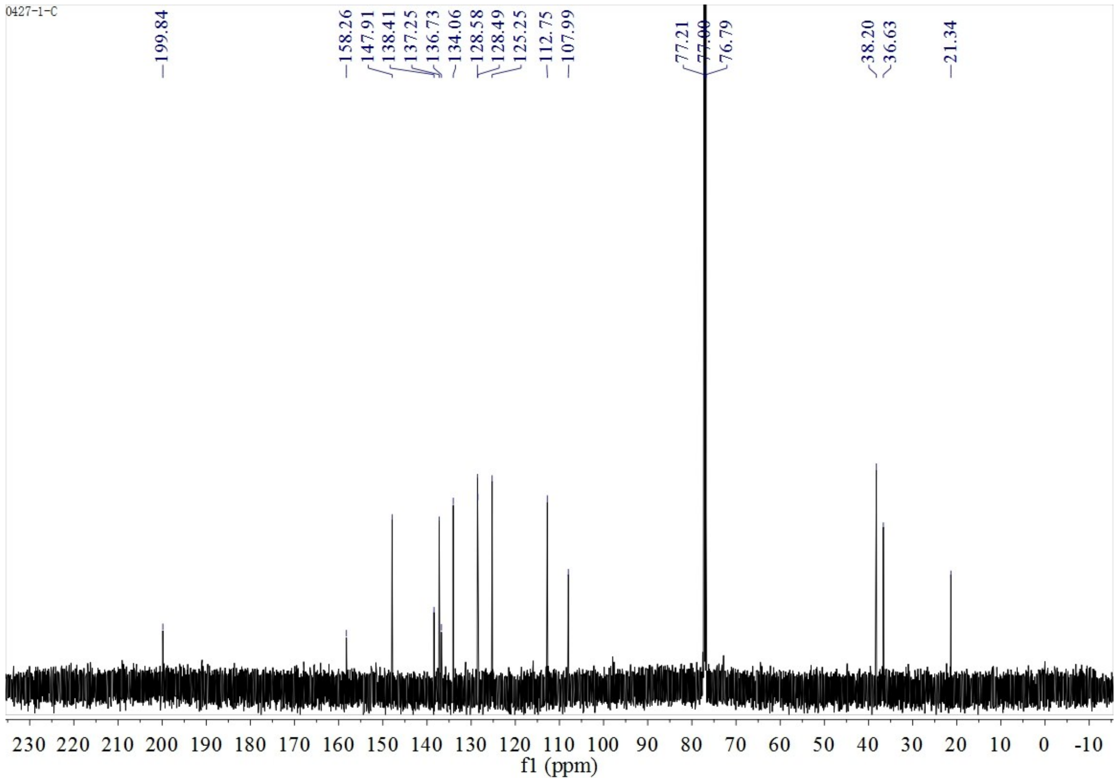
0427-1-C
199.84
158.26
147.91
138.41
137.25
136.73
134.06
128.58
128.49
125.25
112.75
107.99
77.21
77.00
76.79
38.20
36.63
21.34
230 220 210 200 190 180 170 160 150 140 130 120 110 100 90 80 70 60 50 40 30 20 10 0 -10
f1 (ppm)

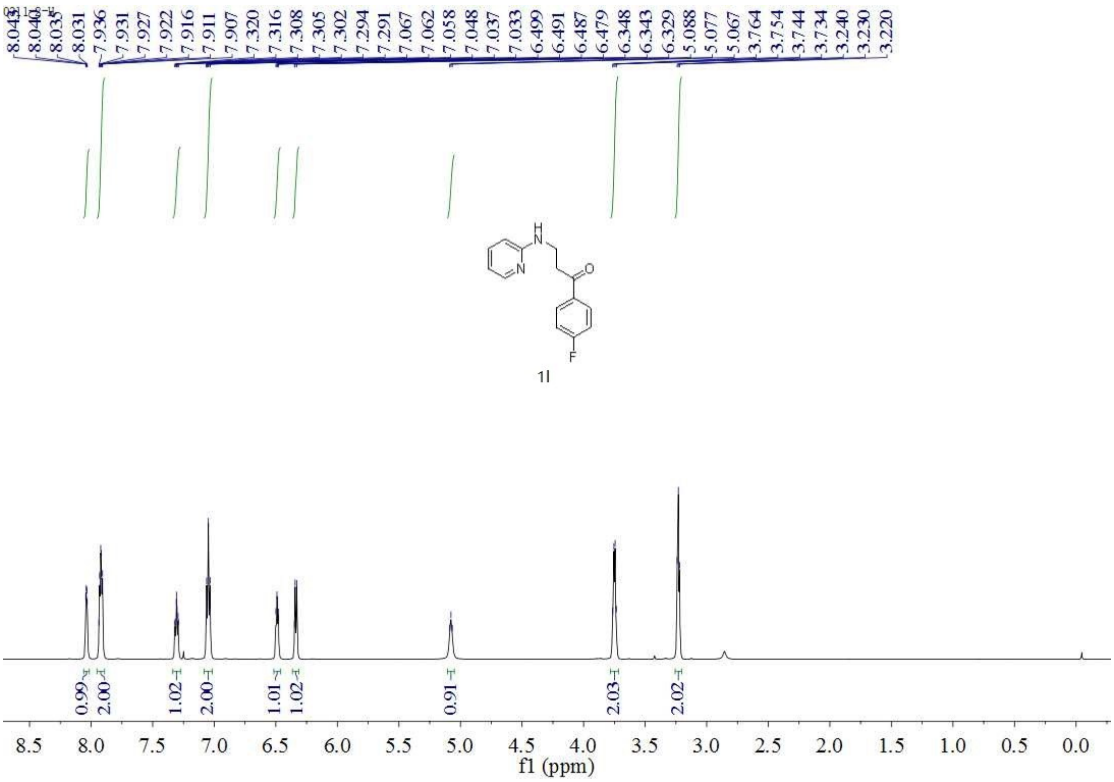
8.040
8.035
8.031
7.936
7.931
7.927
7.922
7.916
7.911
7.907
7.320
7.316
7.308
7.305
7.302
7.294
7.291
7.067
7.062
7.058
7.048
7.037
7.033
6.499
6.491
6.487
6.479
6.348
6.343
6.329
5.088
5.077
5.067
3.764
3.754
3.744
3.734
3.240
3.230
3.220
1l
0.99
2.00
1.02
2.00
1.01
1.02
0.91
2.03
2.02
8.5 8.0 7.5 7.0 6.5 6.0 5.5 5.0 4.5 4.0 3.5 3.0 2.5 2.0 1.5 1.0 0.5 0.0
f1 (ppm)

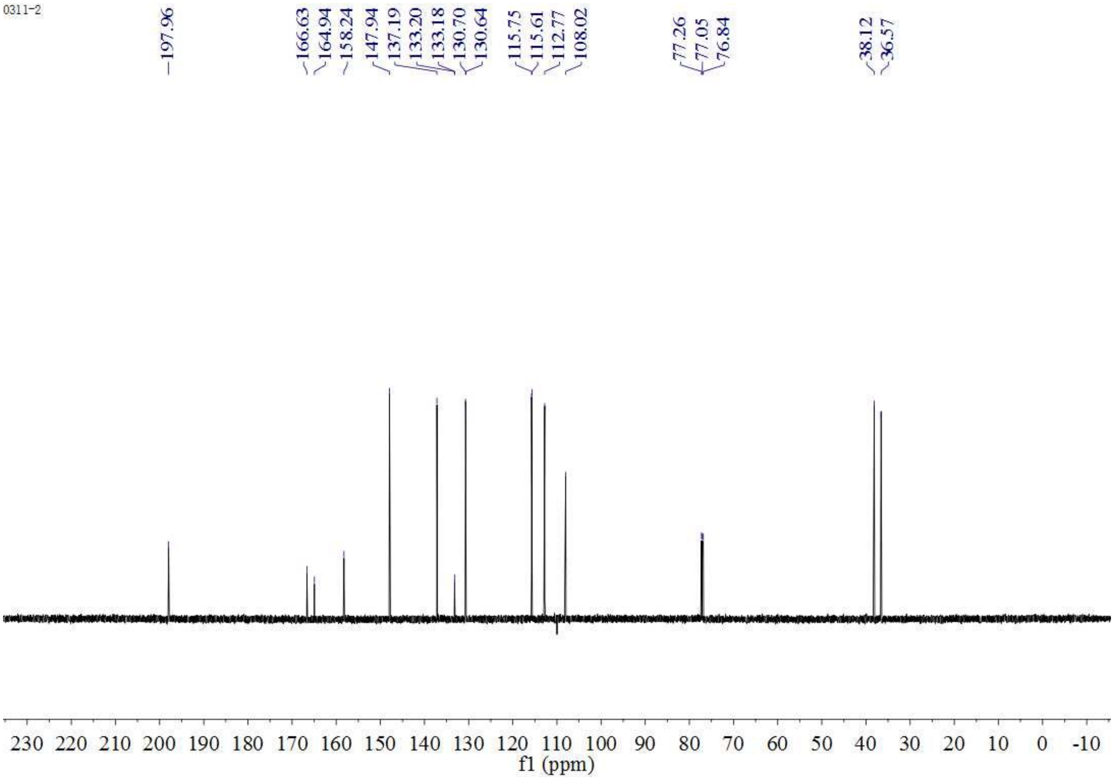
0311-2
197.96
166.63
164.94
158.24
147.94
137.19
133.20
133.18
130.70
130.64
115.75
115.61
112.77
108.02
77.26
77.05
76.84
38.12
36.57
230 220 210 200 190 180 170 160 150 140 130 120 110 100 90 80 70 60 50 40 30 20 10 0 -10
f1 (ppm)

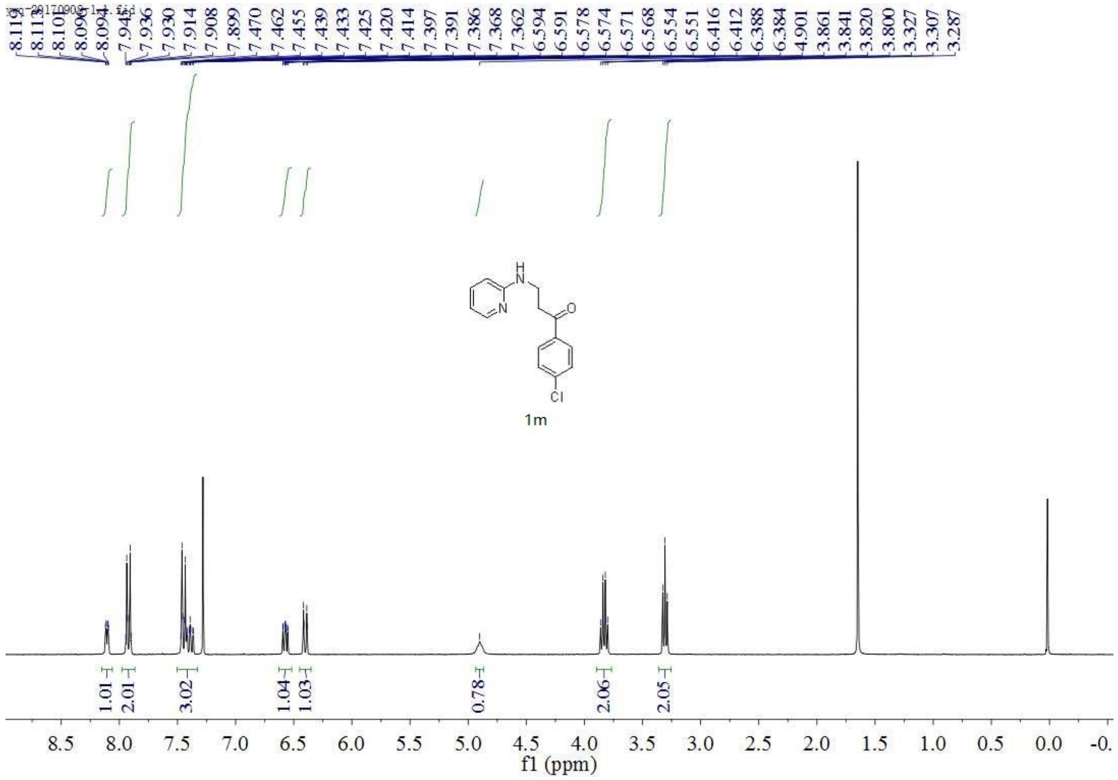
8.113
8.101
8.094
7.936
7.930
7.914
7.908
7.899
7.470
7.462
7.455
7.439
7.433
7.425
7.420
7.414
7.397
7.391
7.386
7.368
7.362
6.594
6.591
6.578
6.574
6.571
6.568
6.554
6.551
6.416
6.412
6.388
6.384
4.901
3.861
3.841
3.820
3.800
3.327
3.307
3.287
H
N
N
O
Cl
1m
1.01
2.01
3.02
1.04
1.03
0.78
2.06
2.05
8.5 8.0 7.5 7.0 6.5 6.0 5.5 5.0 4.5 4.0 3.5 3.0 2.5 2.0 1.5 1.0 0.5 0.0 -0.
f1 (ppm)

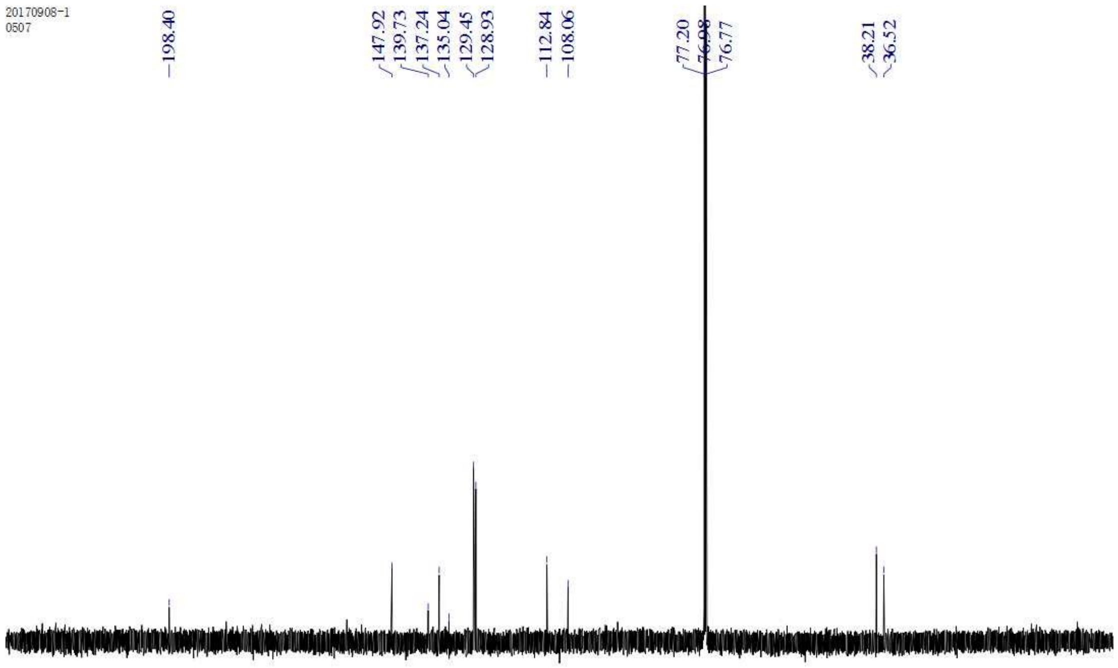
20170908-1
0507
198.40
147.92
139.73
137.24
135.04
129.45
128.93
112.84
108.06
77.20
76.98
76.77
38.21
36.52
230 220 210 200 190 180 170 160 150 140 130 120 110 100 90 80 70 60 50 40 30 20 10 0 -10
f1 (ppm)

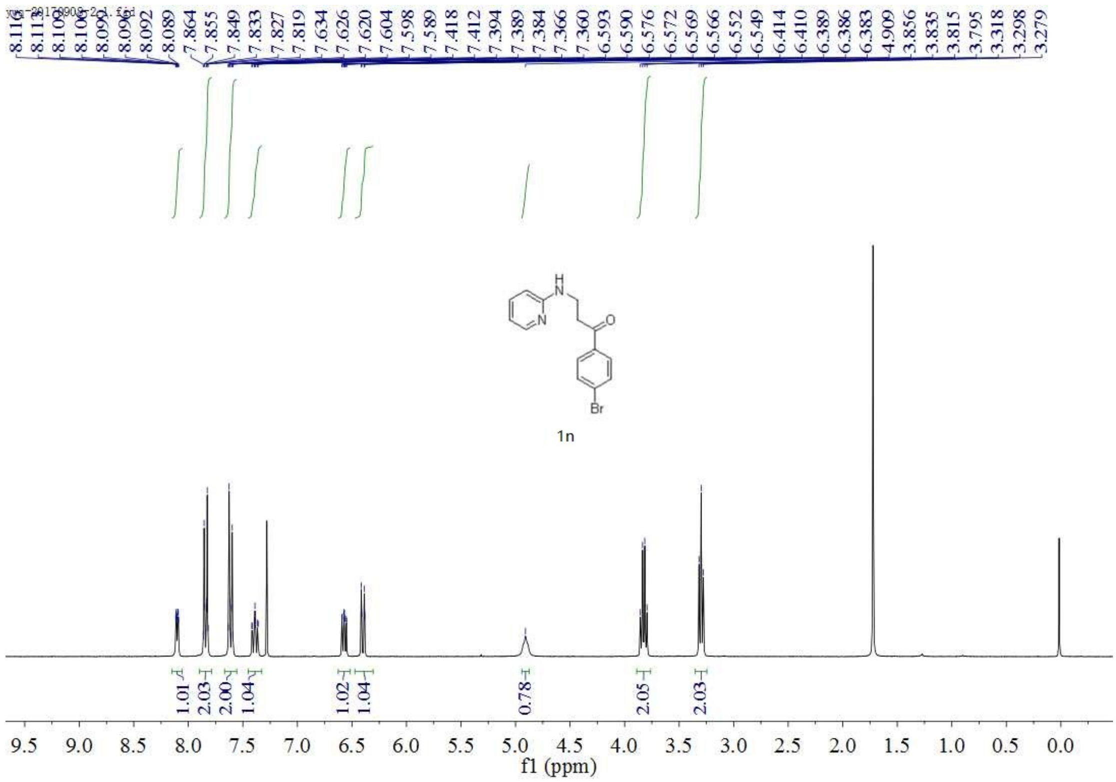
8.092
8.089
7.864
7.855
7.849
7.833
7.827
7.819
7.634
7.626
7.620
7.604
7.598
7.589
7.418
7.412
7.394
7.389
7.384
7.366
7.360
6.593
6.590
6.576
6.572
6.569
6.566
6.552
6.549
6.414
6.410
6.389
6.386
6.383
4.909
3.856
3.835
3.815
3.795
3.318
3.298
3.279
Br
1n
1.01
2.03
2.00
1.04
1.02
1.04
0.78
2.05
2.03
9.5 9.0 8.5 8.0 7.5 7.0 6.5 6.0 5.5 5.0 4.5 4.0 3.5 3.0 2.5 2.0 1.5 1.0 0.5 0.0
f1 (ppm)

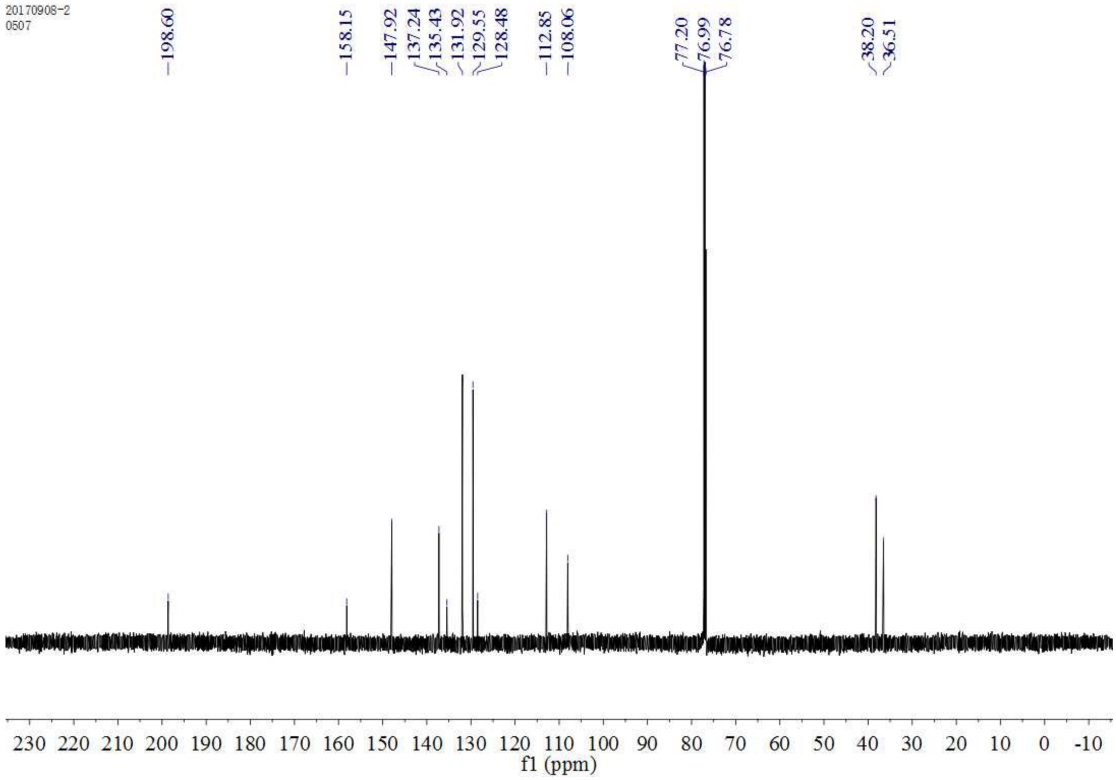
20170908-2
0507
198.60
158.15
147.92
137.24
135.43
131.92
129.55
128.48
112.85
108.06
77.20
76.99
76.78
38.20
36.51
230 220 210 200 190 180 170 160 150 140 130 120 110 100 90 80 70 60 50 40 30 20 10 0 -10
f1 (ppm)

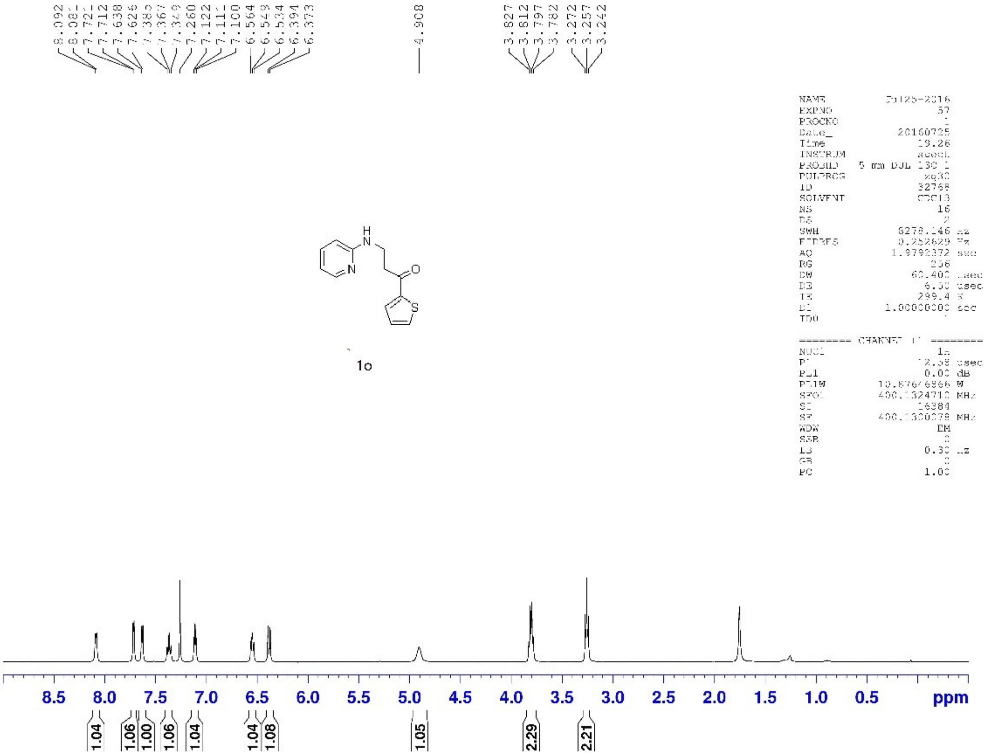
8.092
8.081
7.721
7.712
7.638
7.626
7.385
7.367
7.349
7.260
7.122
7.111
7.100
6.564
6.549
6.534
6.394
6.373
4.908
3.827
3.812
3.797
3.782
3.272
3.257
3.242
1o
8.5 8.0 7.5 7.0 6.5 6.0 5.5 5.0 4.5 4.0 3.5 3.0 2.5 2.0 1.5 1.0 0.5 ppm
1.04 1.06 1.00 1.06 1.04 1.04 1.08 1.05 2.29 2.21

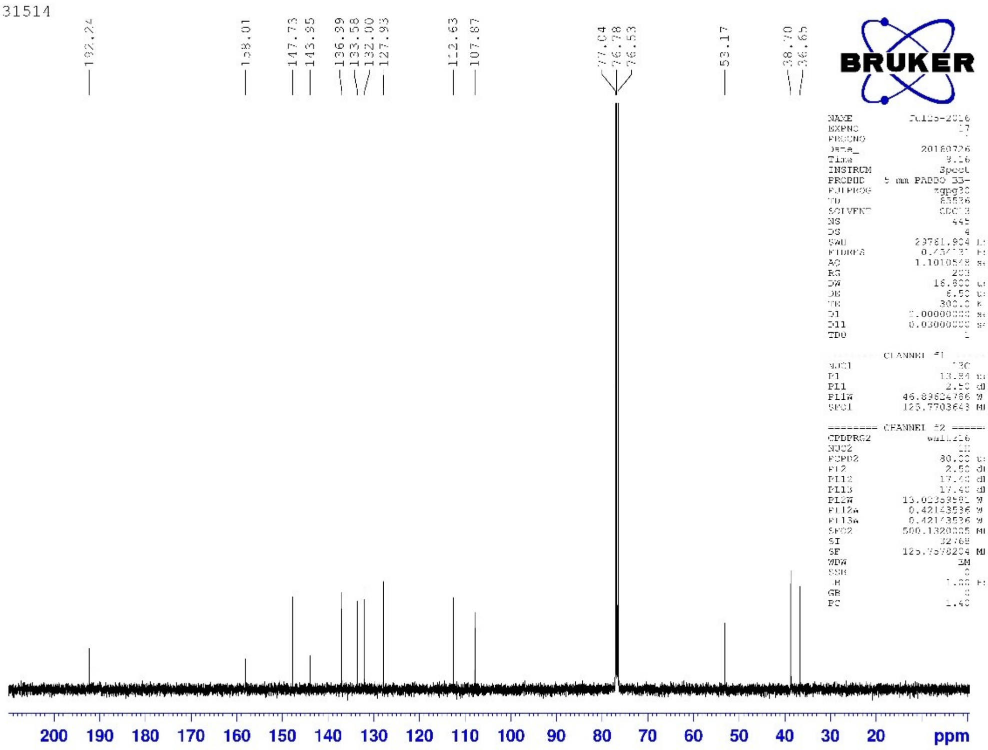
31514
192.24
158.01
147.73
143.95
136.99
133.58
132.00
127.93
112.63
107.87
77.04
76.78
76.53
53.17
38.70
36.65
200 190 180 170 160 150 140 130 120 110 100 90 80 70 60 50 40 30 20 ppm

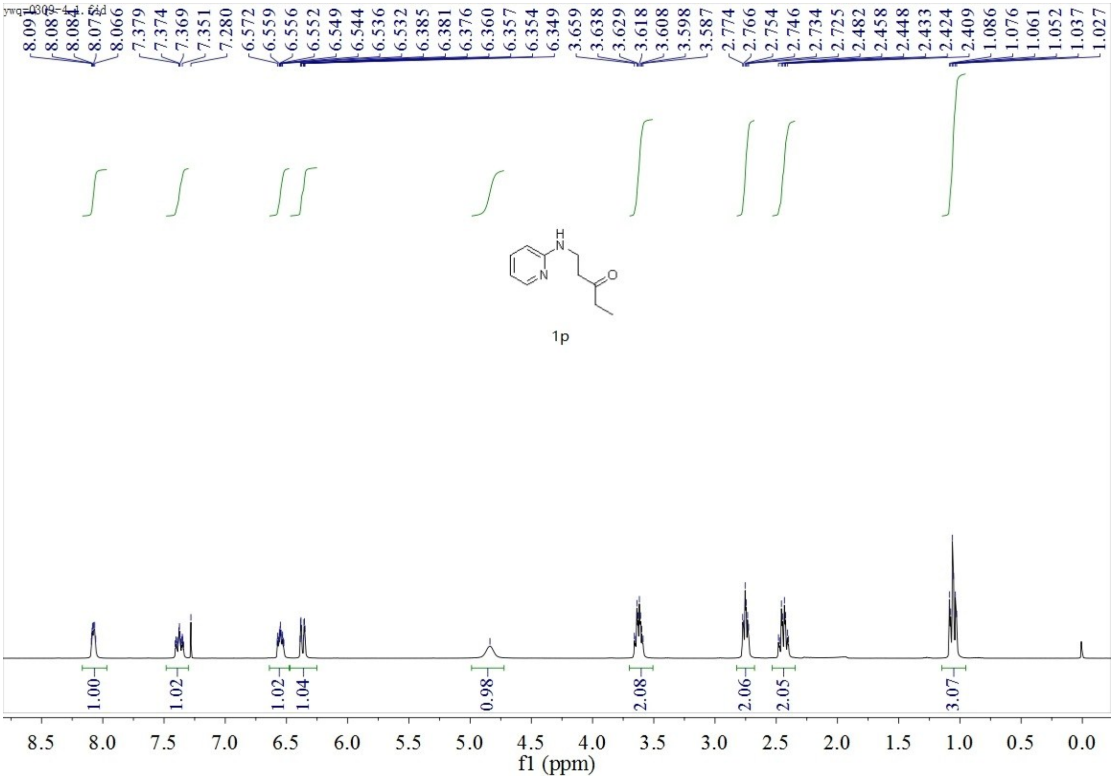
7.379
7.374
7.369
7.351
7.280
6.572
6.559
6.556
6.552
6.549
6.544
6.536
6.532
6.385
6.381
6.376
6.360
6.357
6.354
6.349
3.659
3.638
3.629
3.618
3.608
3.598
3.587
2.774
2.766
2.754
2.746
2.734
2.725
2.482
2.458
2.448
2.433
2.424
2.409
1.086
1.076
1.061
1.052
1.037
1.027
H
N
O
1p
1.00
1.02
1.02
1.04
0.98
2.08
2.06
2.05
3.07
8.5 8.0 7.5 7.0 6.5 6.0 5.5 5.0 4.5 4.0 3.5 3.0 2.5 2.0 1.5 1.0 0.5 0.0
f1 (ppm)

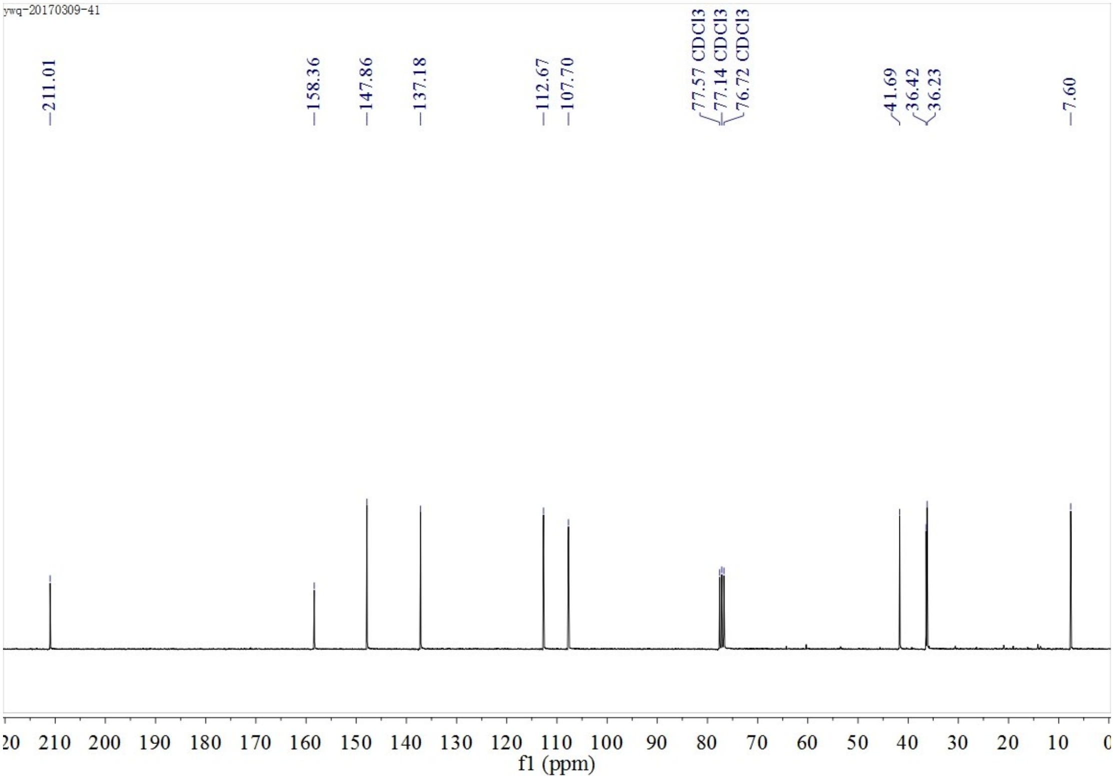
ywq-20170309-41
211.01
158.36
147.86
137.18
112.67
107.70
77.57 CDCl3
77.14 CDCl3
76.72 CDCl3
41.69
36.42
36.23
7.60
210 200 190 180 170 160 150 140 130 120 110 100 90 80 70 60 50 40 30 20 10
f1 (ppm)

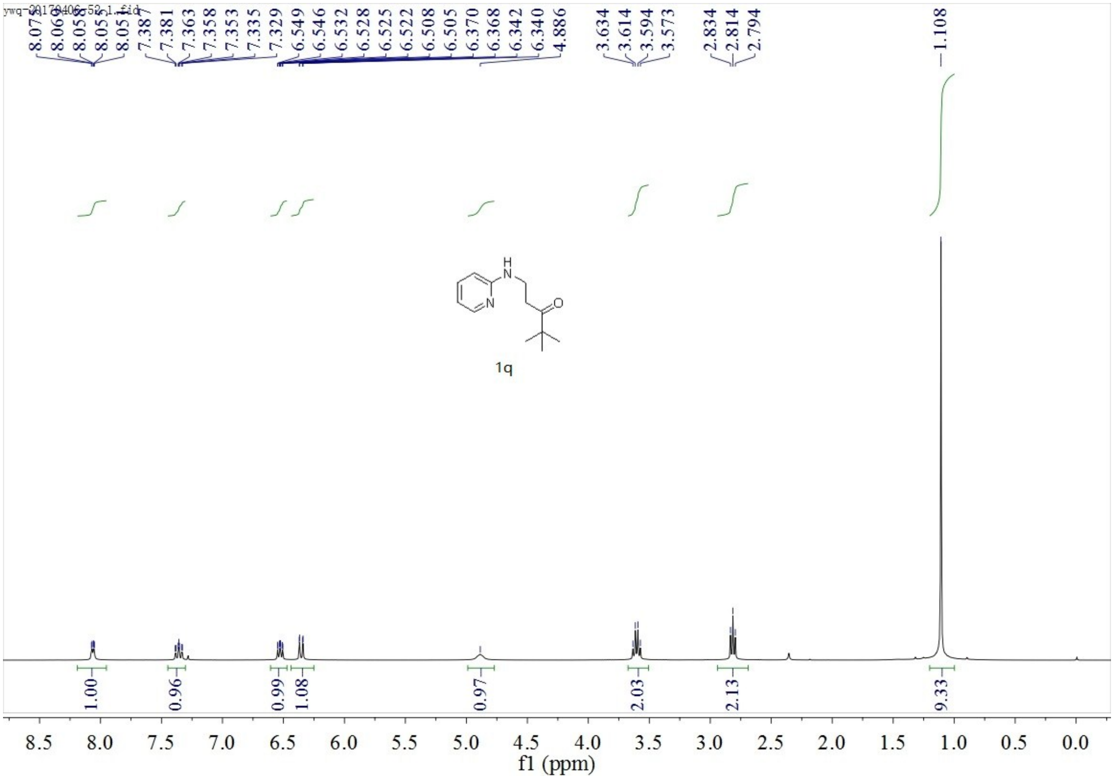
8.070
8.066
8.055
8.051
7.387
7.381
7.363
7.358
7.353
7.335
7.329
6.549
6.546
6.532
6.528
6.525
6.522
6.508
6.505
6.370
6.368
6.342
6.340
4.886
3.634
3.614
3.594
3.573
2.834
2.814
2.794
1.108
1q
1.00
0.96
0.99
1.08
0.97
2.03
2.13
9.33
f1 (ppm)

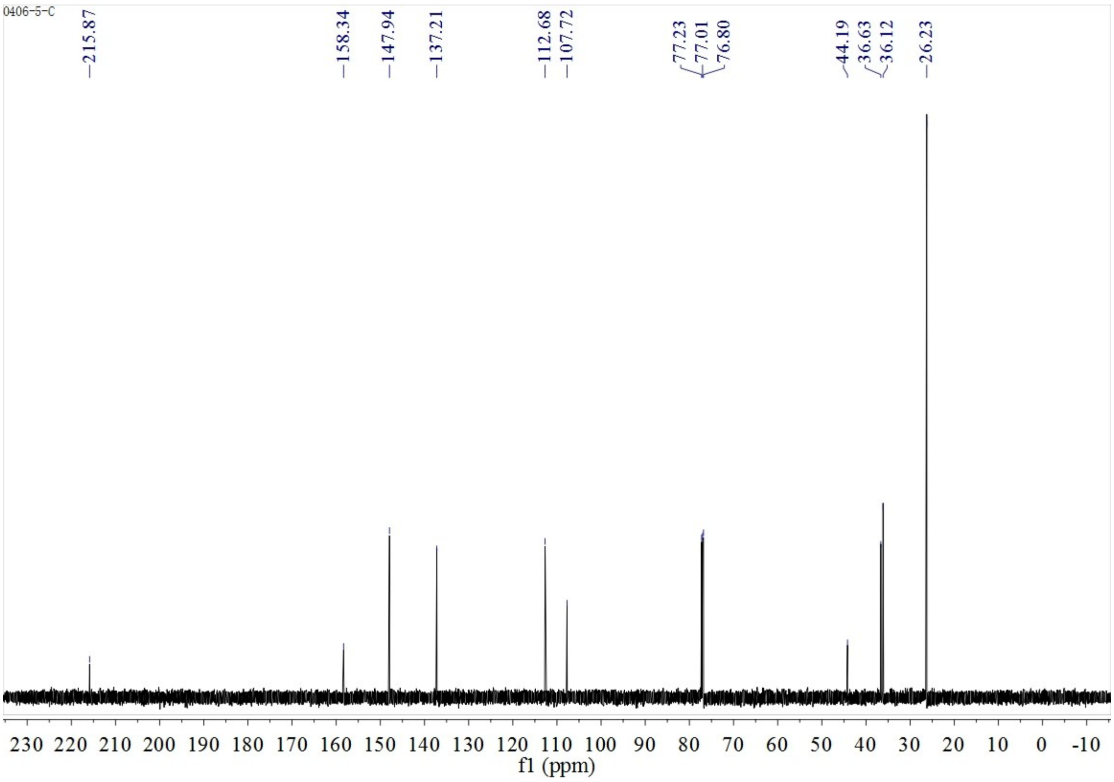
0406-5-C
215.87
158.34
147.94
137.21
112.68
107.72
77.23
77.01
76.80
44.19
36.63
36.12
26.23
f1 (ppm)

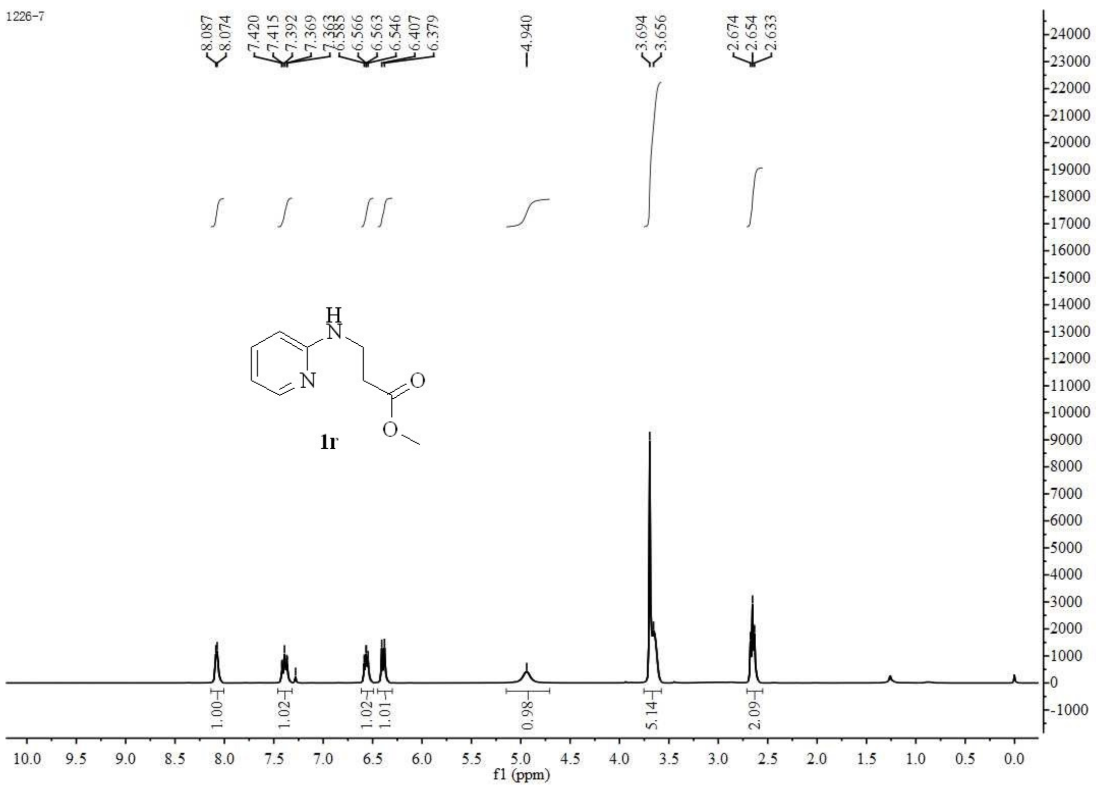
1226-7
8.087
8.074
7.420
7.415
7.392
7.369
7.383
6.585
6.566
6.563
6.546
6.407
6.379
4.940
3.694
3.656
2.674
2.654
2.633
1r
1.00
1.02
1.02
1.01
0.98
5.14
2.09
f1 (ppm)

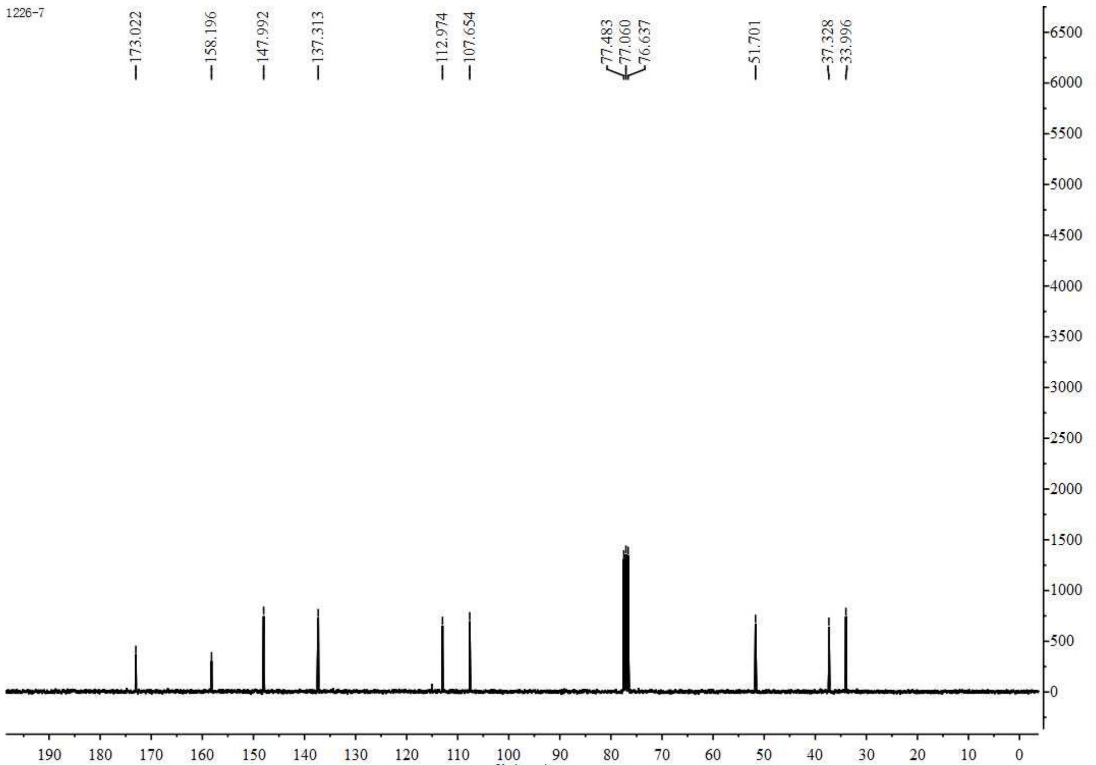
1226-7
173.022
158.196
147.992
137.313
112.974
107.654
77.483
77.060
76.637
51.701
37.328
33.996
f1 (ppm)

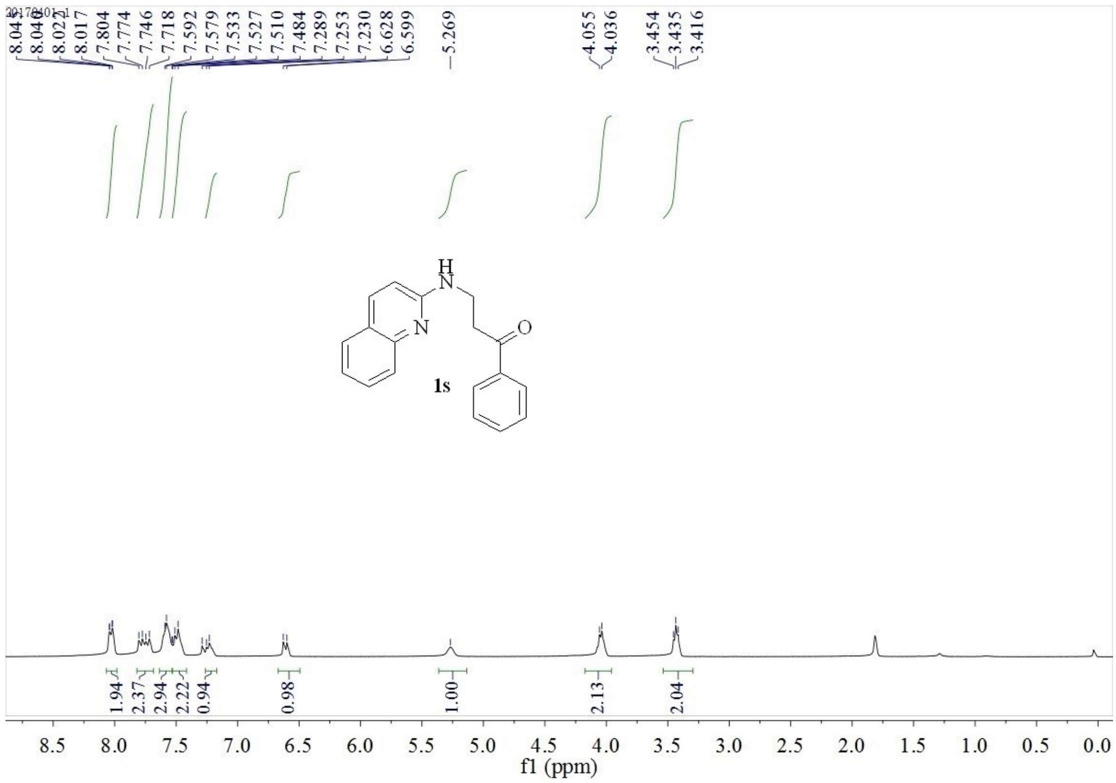

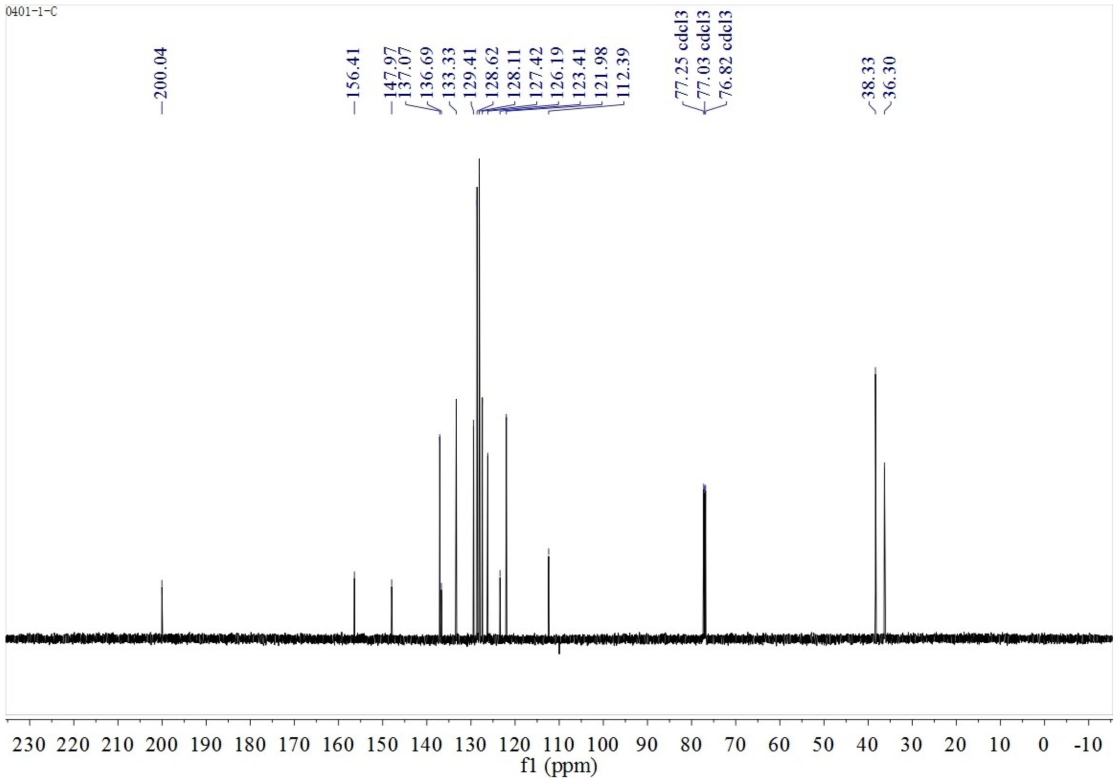

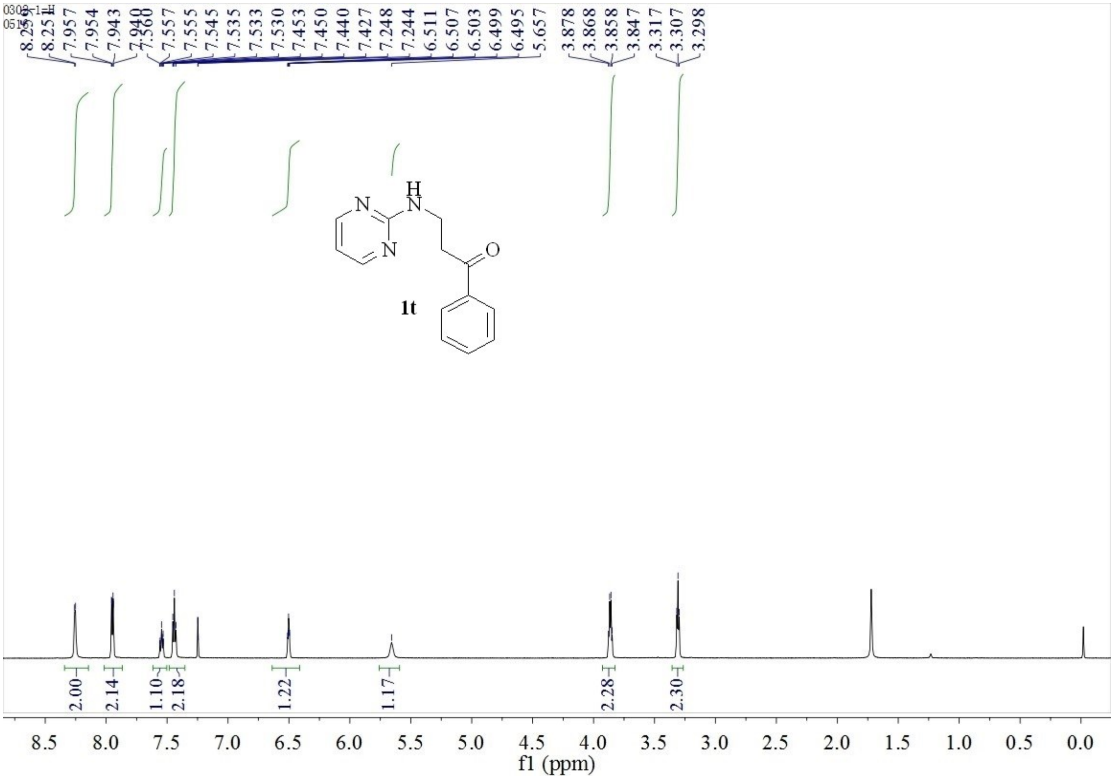
8.257
8.251
7.957
7.954
7.943
7.940
7.560
7.557
7.555
7.545
7.535
7.533
7.530
7.453
7.450
7.440
7.427
7.248
7.244
6.511
6.507
6.503
6.499
6.495
5.657
3.878
3.868
3.858
3.847
3.317
3.307
3.298
1t
2.00
2.14
1.10
2.18
1.22
1.17
2.28
2.30
f1 (ppm)

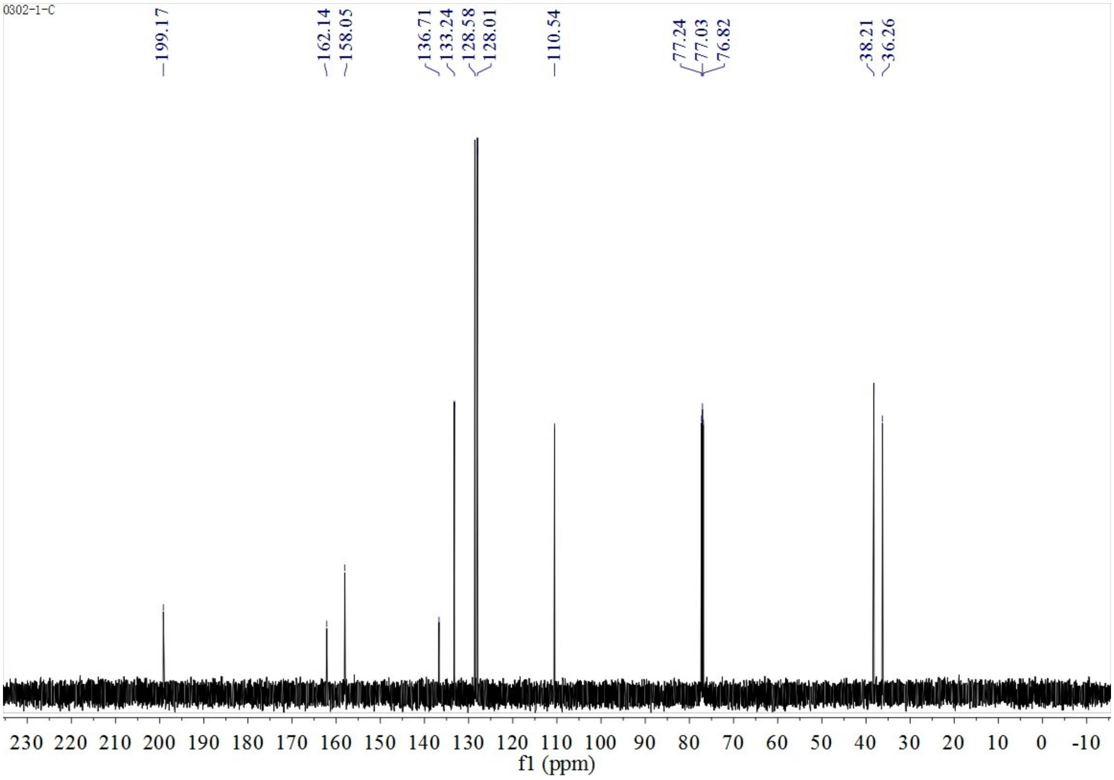
0302-1-C
199.17
162.14
158.05
136.71
133.24
128.58
128.01
110.54
77.24
77.03
76.82
38.21
36.26
f1 (ppm)

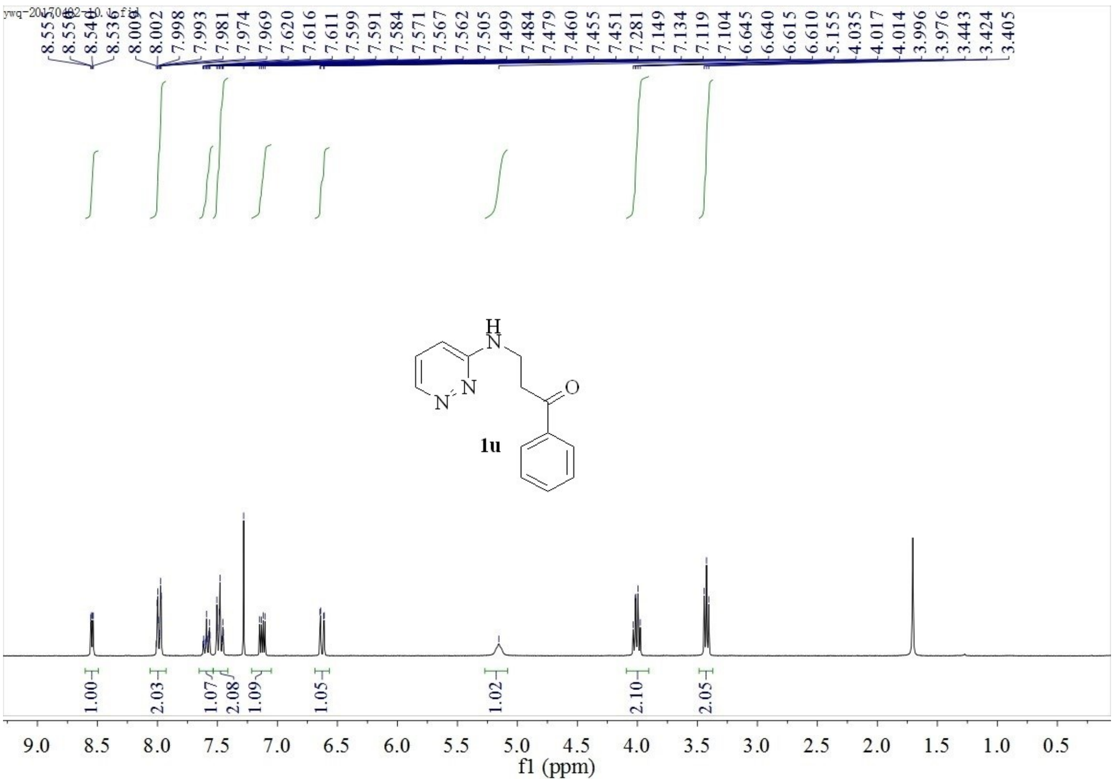
ywq-20170402-10.1.fid
8.555 8.550 8.541 8.536 8.009 8.002 7.998 7.993 7.981 7.974 7.969 7.620 7.616 7.611 7.599 7.591 7.584 7.571 7.567 7.562 7.505 7.499 7.484 7.479 7.460 7.455 7.451 7.281 7.149 7.134 7.119 7.104 6.645 6.640 6.615 6.610 5.155 4.035 4.017 4.014 3.996 3.976 3.443 3.424 3.405
H
N
N
N
O
1u
1.00 2.03 1.07 2.08 1.09 1.05 1.02 2.10 2.05
9.0 8.5 8.0 7.5 7.0 6.5 6.0 5.5 5.0 4.5 4.0 3.5 3.0 2.5 2.0 1.5 1.0 0.5
f1 (ppm)

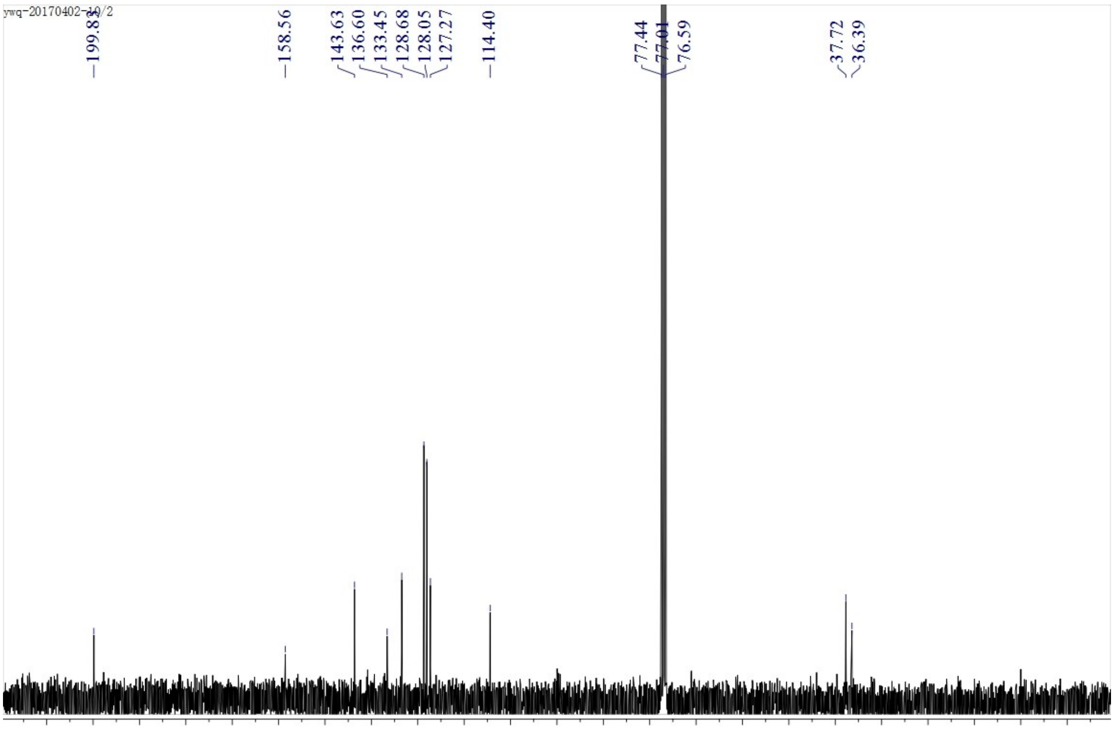
ywq-20170402-10/2
199.82
158.56
143.63 136.60 133.45 128.68 128.05 127.27
114.40
77.44 77.01 76.59
37.72 36.39
210 200 190 180 170 160 150 140 130 120 110 100 90 80 70 60 50 40 30 20 10 0 -10
f1 (ppm)

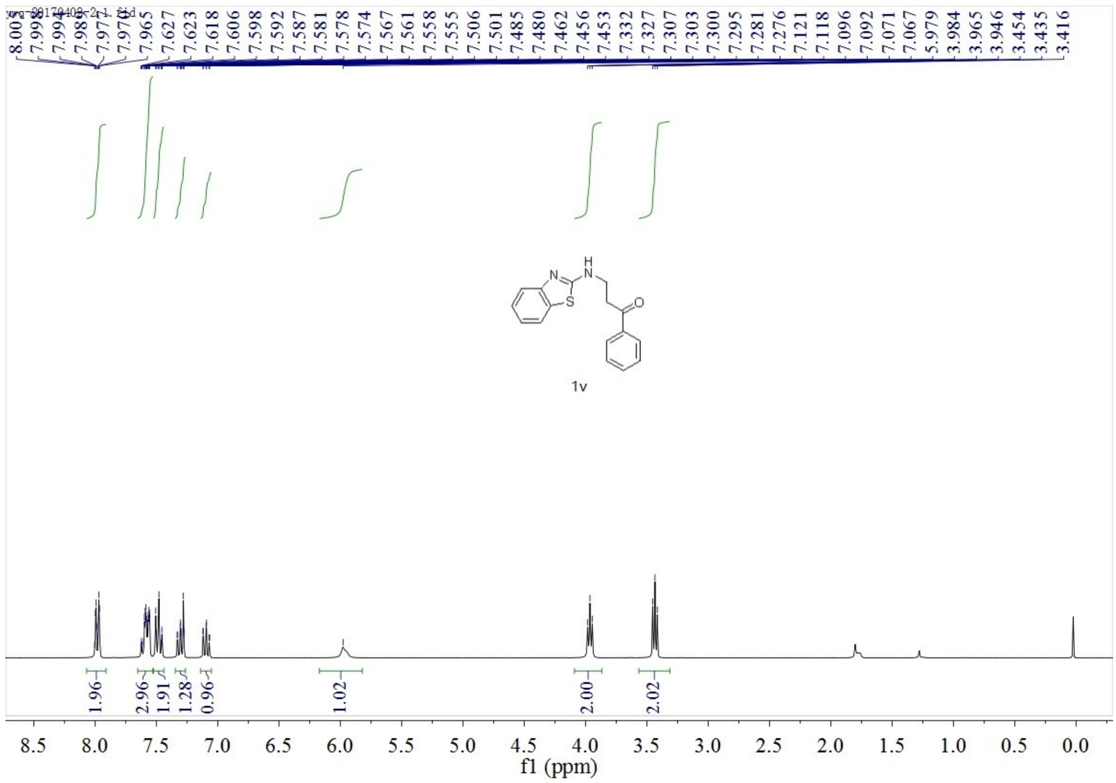
ywq-20170402-2.1.fid
8.003
7.998
7.994
7.989
7.977
7.970
7.965
7.627
7.623
7.618
7.606
7.598
7.592
7.587
7.581
7.578
7.574
7.567
7.561
7.558
7.555
7.506
7.501
7.485
7.480
7.462
7.456
7.453
7.332
7.327
7.307
7.303
7.300
7.295
7.281
7.276
7.121
7.118
7.096
7.092
7.071
7.067
5.979
3.984
3.965
3.946
3.454
3.435
3.416
N
H
S
O
1v
1.96
2.96
1.91
1.28
0.96
1.02
2.00
2.02
8.5 8.0 7.5 7.0 6.5 6.0 5.5 5.0 4.5 4.0 3.5 3.0 2.5 2.0 1.5 1.0 0.5 0.0
f1 (ppm)

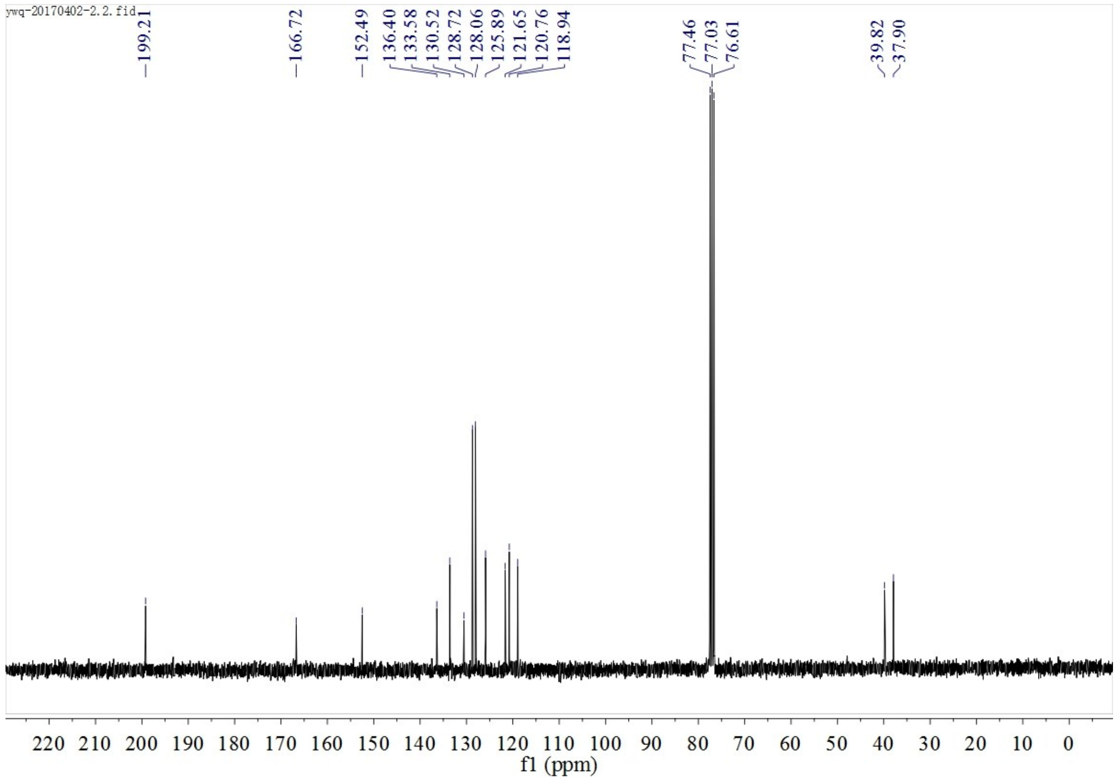
ywq-20170402-2.2.fid
199.21
166.72
152.49
136.40
133.58
130.52
128.72
128.06
125.89
121.65
120.76
118.94
77.46
77.03
76.61
39.82
37.90
220 210 200 190 180 170 160 150 140 130 120 110 100 90 80 70 60 50 40 30 20 10 0
f1 (ppm)

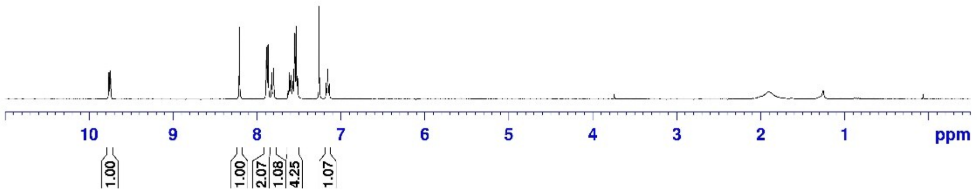

10
9
8
7
6
5
4
3
2
1
ppm

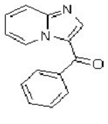

2a

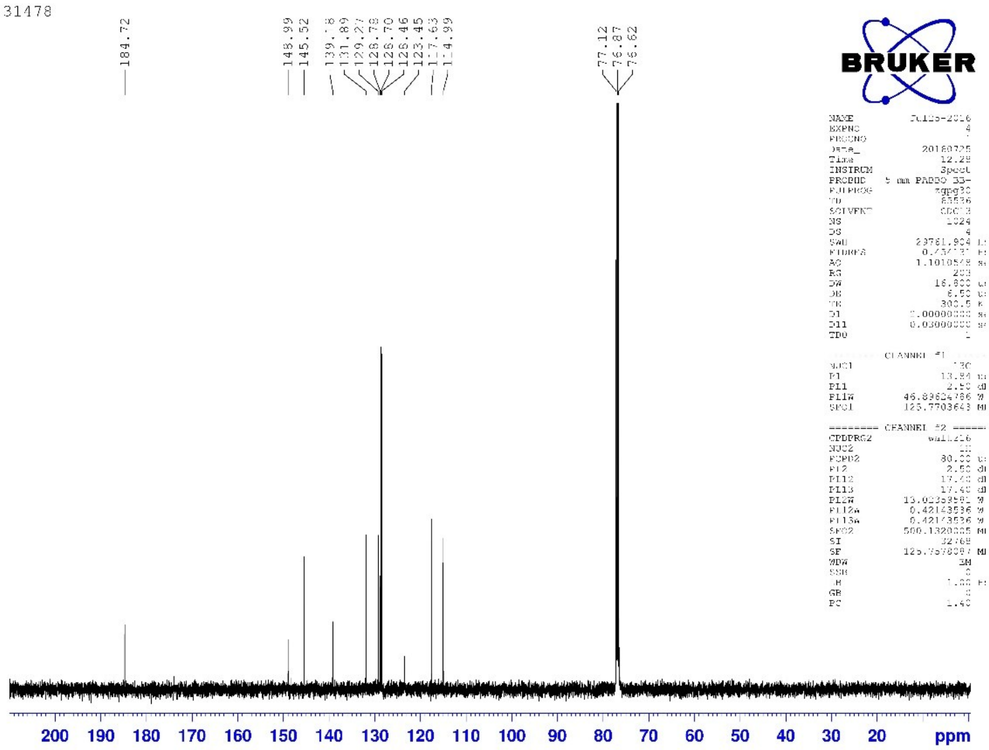

184.72
148.99
145.52
77.12
76.87
76.62
200
190
180
170
160
150
140
130
120
110
100
90
80
70
60
50
40
30
20
ppm

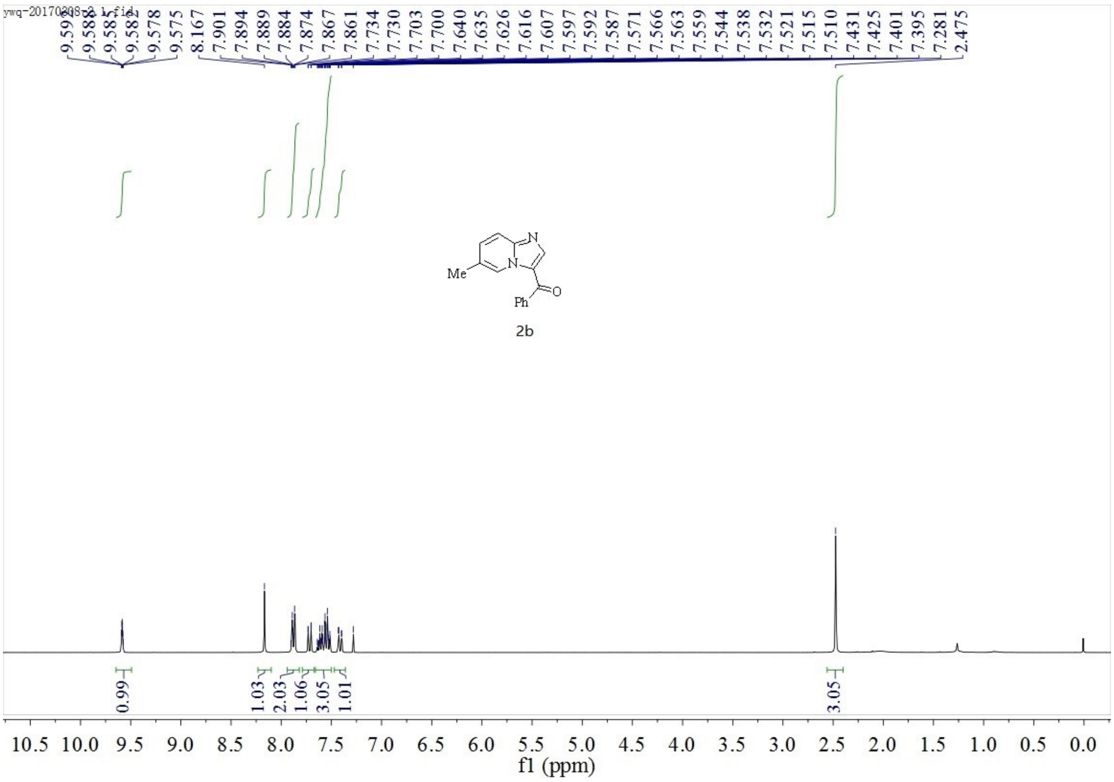
9.593 9.588 9.583 9.582 9.578 9.575 8.167 7.901 7.894 7.889 7.884 7.874 7.867 7.861 7.734 7.730 7.703 7.700 7.640 7.635 7.626 7.616 7.607 7.597 7.592 7.587 7.571 7.566 7.563 7.559 7.544 7.538 7.532 7.521 7.515 7.510 7.431 7.425 7.401 7.395 7.281 2.475
Me
N
N
O
Ph
2b
0.99
1.03
2.03
1.06
3.05
1.01
3.05
10.5 10.0 9.5 9.0 8.5 8.0 7.5 7.0 6.5 6.0 5.5 5.0 4.5 4.0 3.5 3.0 2.5 2.0 1.5 1.0 0.5 0.0
f1 (ppm)

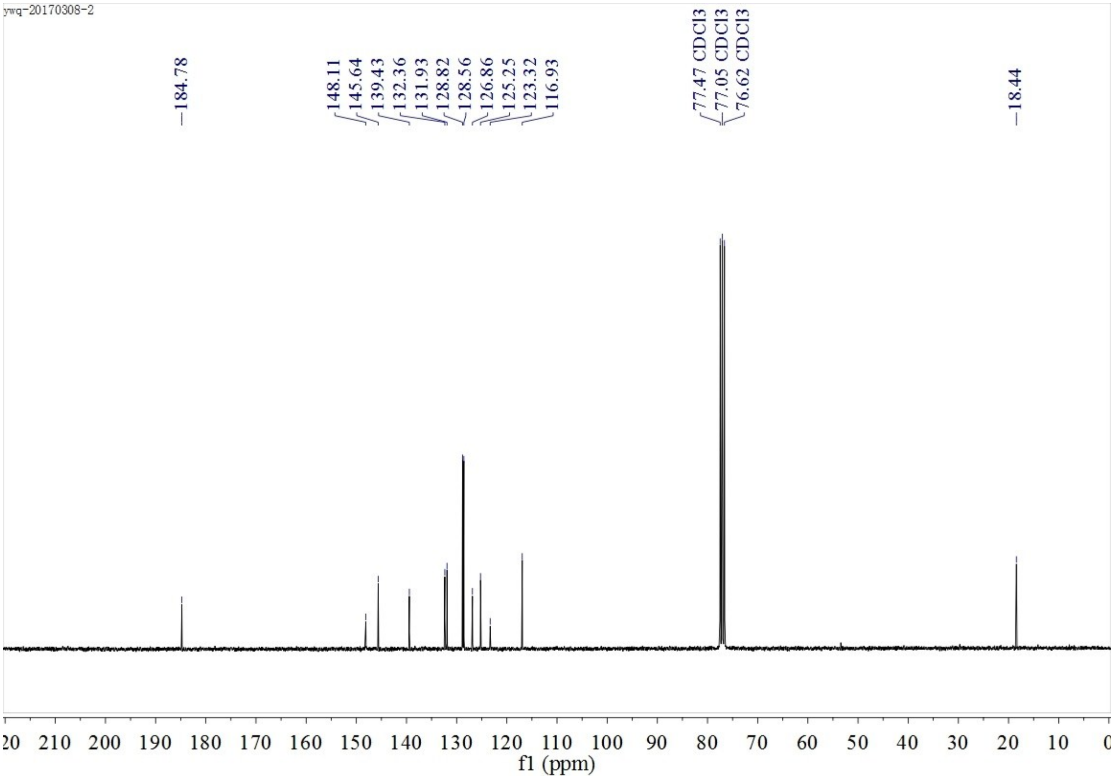
ywq-20170308-2
184.78
148.11 145.64 139.43 132.36 131.93 128.82 128.56 126.86 125.25 123.32 116.93
77.47 CDCl3
77.05 CDCl3
76.62 CDCl3
18.44
210 200 190 180 170 160 150 140 130 120 110 100 90 80 70 60 50 40 30 20 10
f1 (ppm)

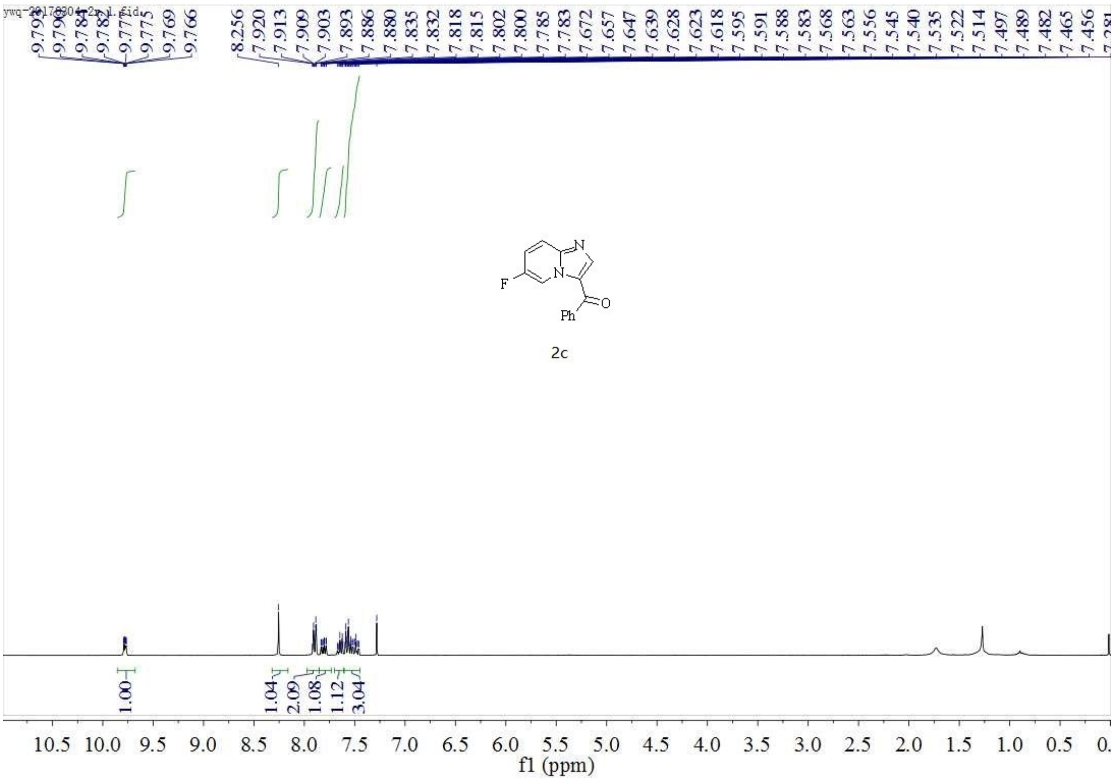

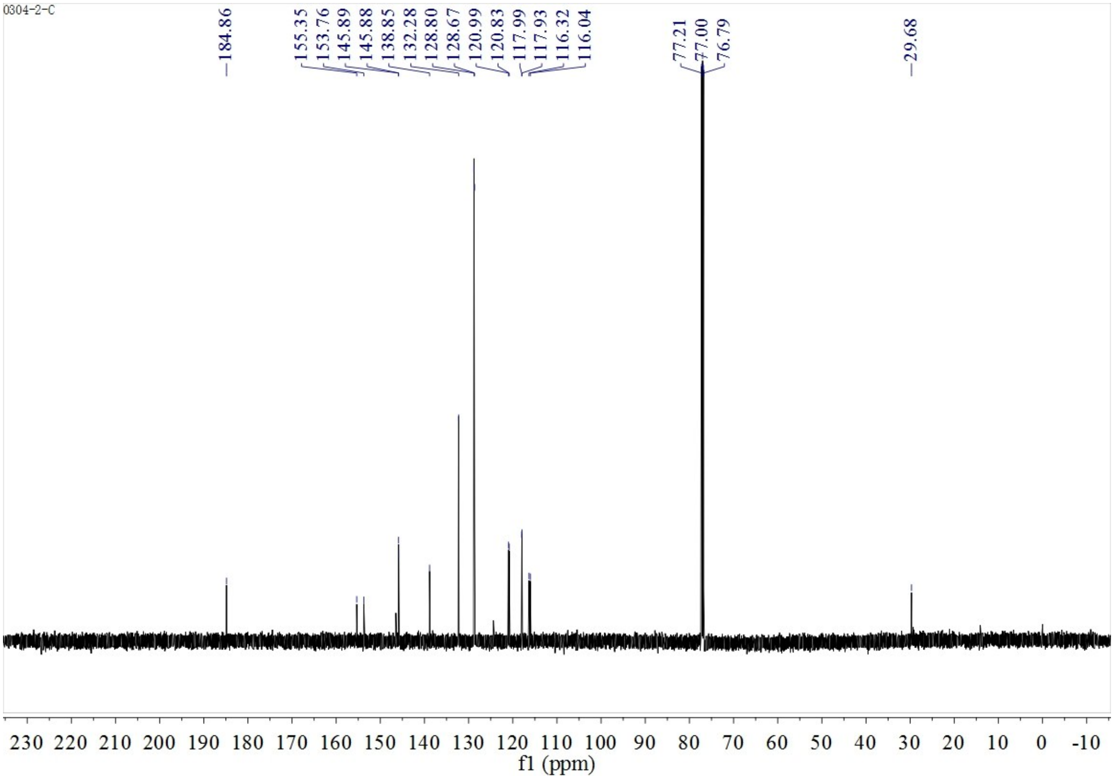

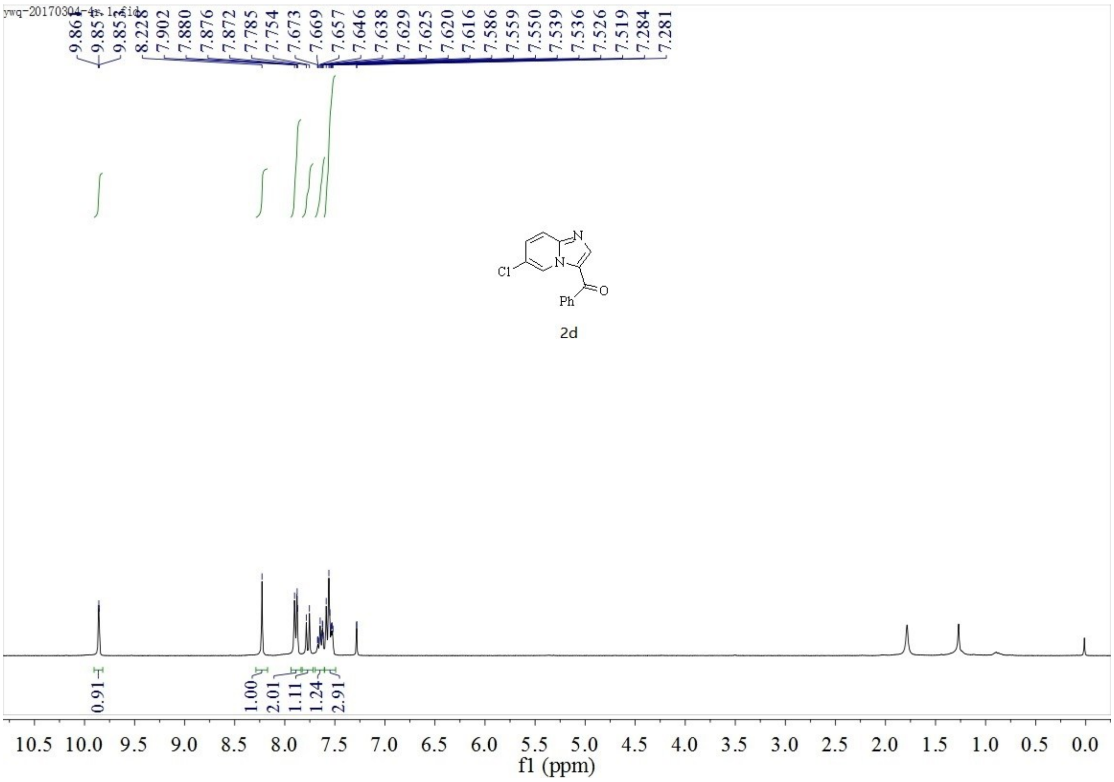
ywq-20170304-4r.1.fid
9.861
9.857
9.853
8.228
7.902
7.880
7.876
7.872
7.785
7.754
7.673
7.669
7.657
7.646
7.638
7.629
7.625
7.620
7.616
7.586
7.559
7.550
7.539
7.536
7.526
7.519
7.284
7.281
Cl
N
N
O
Ph
2d
0.91
1.00
2.01
1.11
1.24
2.91
10.5 10.0 9.5 9.0 8.5 8.0 7.5 7.0 6.5 6.0 5.5 5.0 4.5 4.0 3.5 3.0 2.5 2.0 1.5 1.0 0.5 0.0
f1 (ppm)

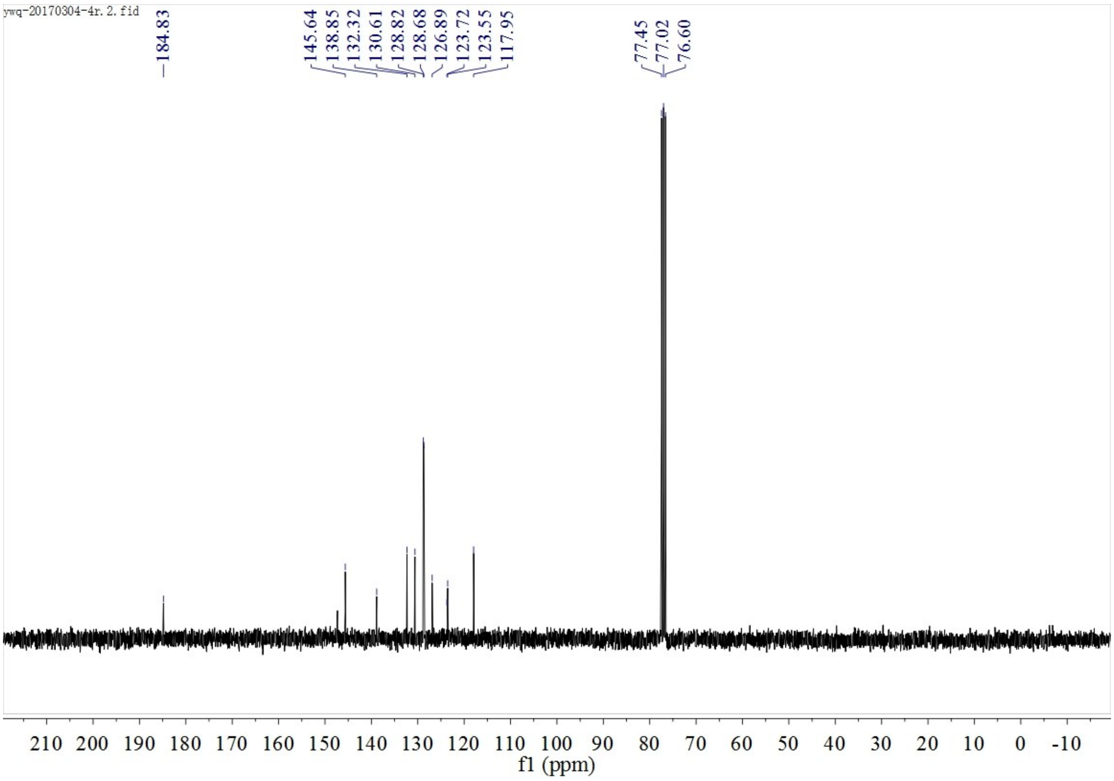
ywq-20170304-4r.2.fid
184.83
145.64
138.85
132.32
130.61
128.82
128.68
126.89
123.72
123.55
117.95
77.45
77.02
76.60
210 200 190 180 170 160 150 140 130 120 110 100 90 80 70 60 50 40 30 20 10 0 -10
f1 (ppm)

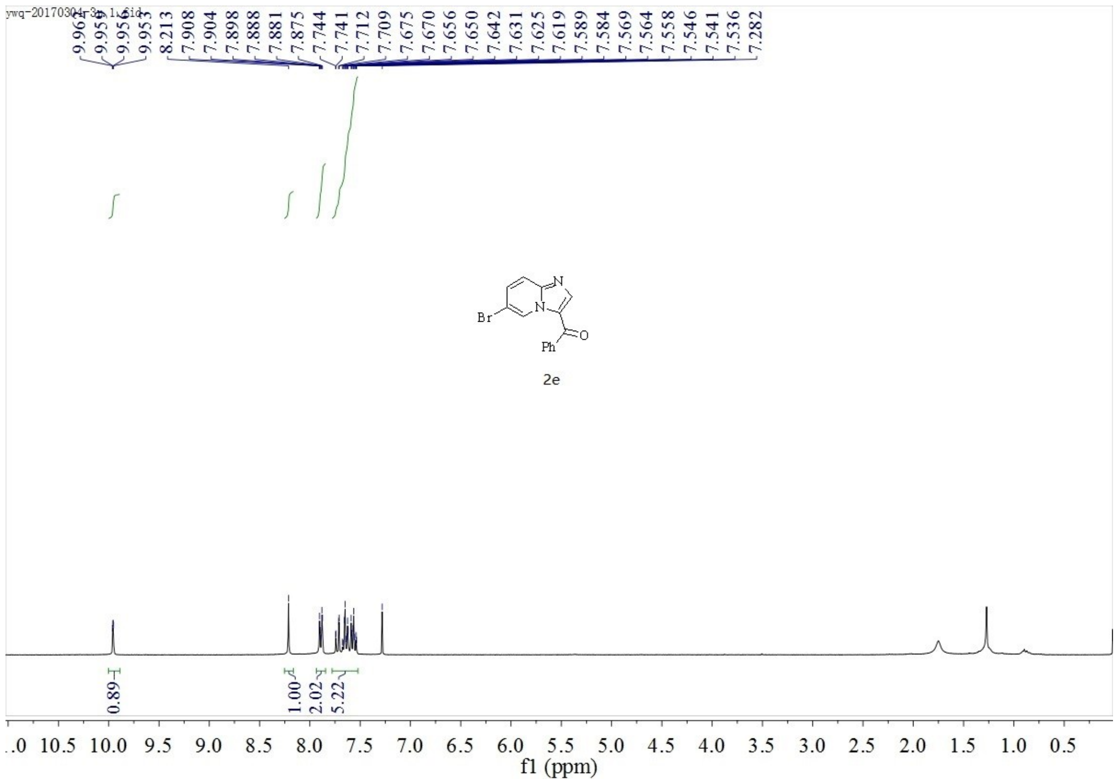
9.961
9.959
9.956
9.953
8.213
7.908
7.904
7.898
7.888
7.881
7.875
7.744
7.741
7.712
7.709
7.675
7.670
7.656
7.650
7.642
7.631
7.625
7.619
7.589
7.584
7.569
7.564
7.558
7.546
7.541
7.536
7.282
Br
N
N
O
Ph
2e
0.89
1.00
2.02
5.22
f1 (ppm)

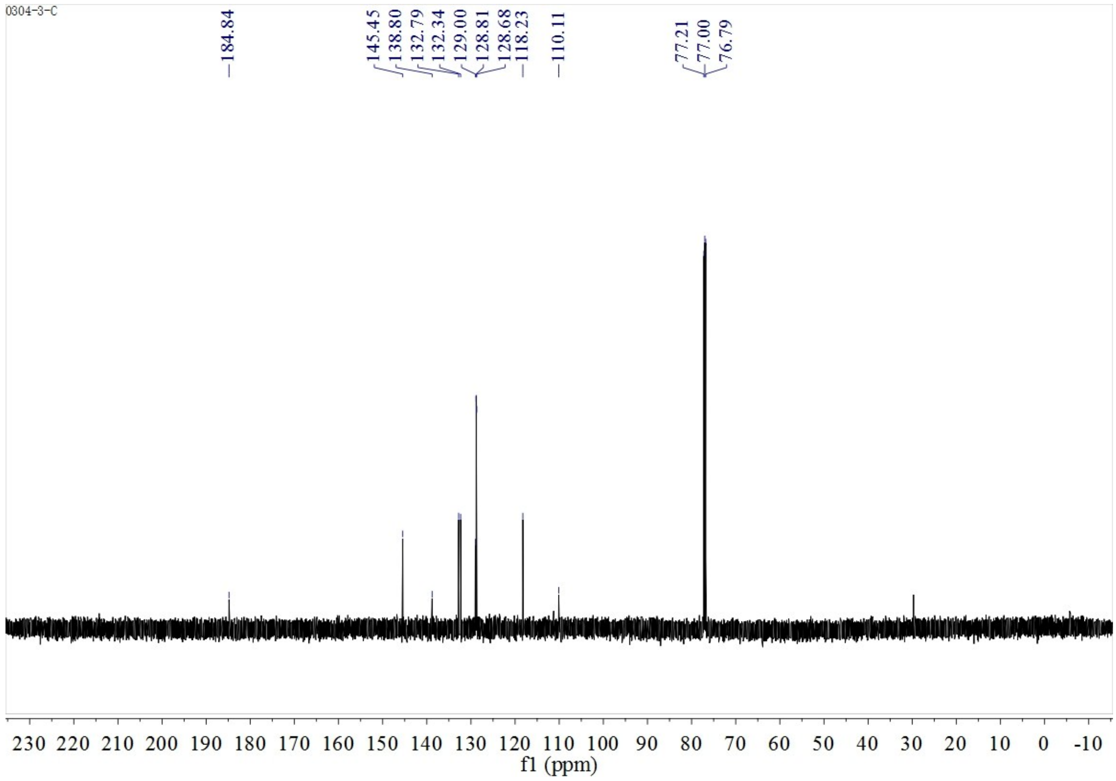
0304-3-C
184.84
145.45
138.80
132.79
132.34
129.00
128.81
128.68
118.23
110.11
77.21
77.00
76.79
f1 (ppm)

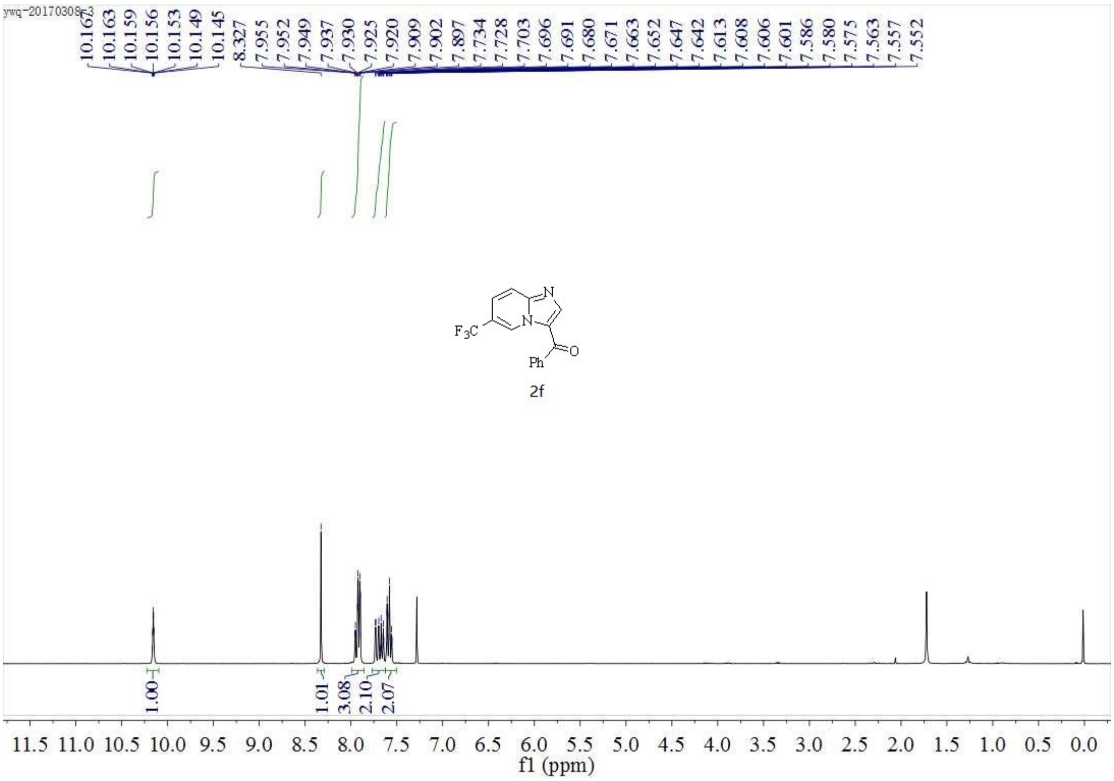
ywq-20170308-3
10.163
10.159
10.156
10.153
10.149
10.145
8.327
7.955
7.952
7.949
7.937
7.930
7.925
7.920
7.909
7.902
7.897
7.734
7.728
7.703
7.696
7.691
7.680
7.671
7.663
7.652
7.647
7.642
7.613
7.608
7.606
7.601
7.586
7.580
7.575
7.563
7.557
7.552
N
N
F_3C
O
Ph
2f
1.00
1.01
3.08
2.10
2.07
11.5 11.0 10.5 10.0 9.5 9.0 8.5 8.0 7.5 7.0 6.5 6.0 5.5 5.0 4.5 4.0 3.5 3.0 2.5 2.0 1.5 1.0 0.5 0.0
f1 (ppm)

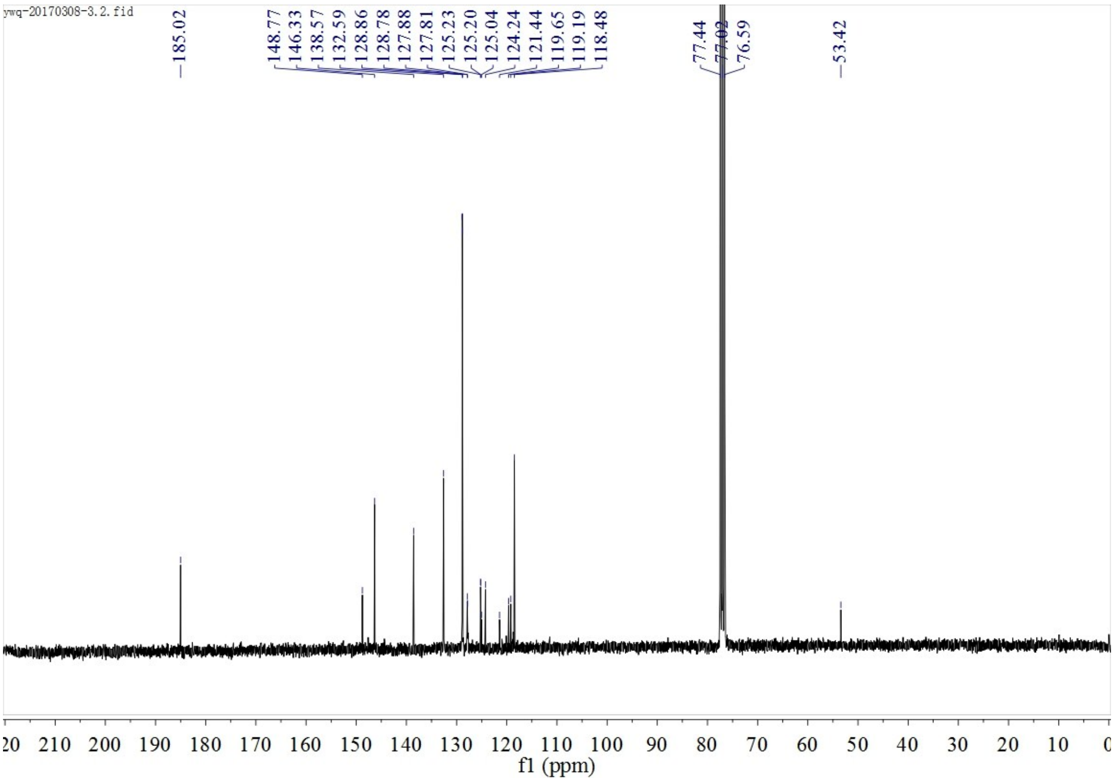
ywq-20170308-3.2.fid
185.02
148.77
146.33
138.57
132.59
128.86
128.78
127.88
127.81
125.23
125.20
125.04
124.24
121.44
119.65
119.19
118.48
77.44
77.02
76.59
53.42
210 200 190 180 170 160 150 140 130 120 110 100 90 80 70 60 50 40 30 20 10
f1 (ppm)

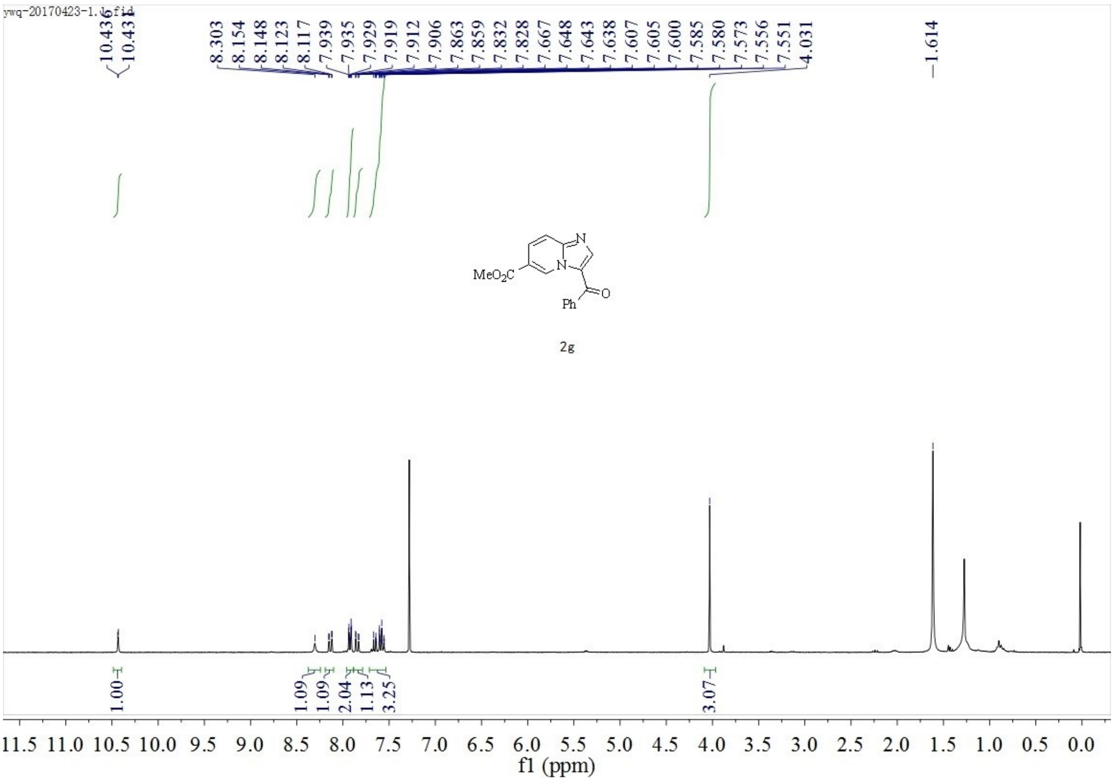
ywq-20170423-1.1.fid
10.436
10.431
8.303
8.154
8.148
8.123
8.117
7.939
7.935
7.929
7.919
7.912
7.906
7.863
7.859
7.832
7.828
7.667
7.648
7.643
7.638
7.607
7.605
7.600
7.585
7.580
7.573
7.556
7.551
4.031
1.614
MeO2C
N
N
Ph
O
2g
1.00
1.09
1.09
2.04
1.13
3.25
3.07
11.5 11.0 10.5 10.0 9.5 9.0 8.5 8.0 7.5 7.0 6.5 6.0 5.5 5.0 4.5 4.0 3.5 3.0 2.5 2.0 1.5 1.0 0.5 0.0
f1 (ppm)

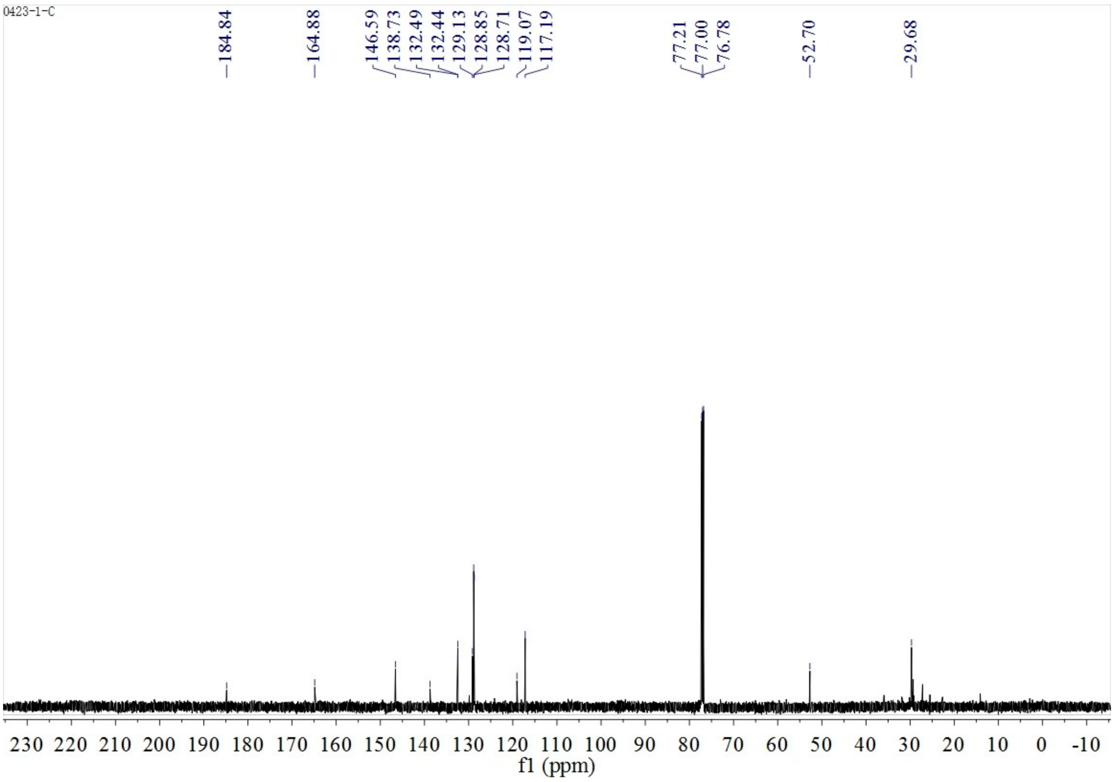
0423-1-C
184.84
164.88
146.59
138.73
132.49
132.44
129.13
128.85
128.71
119.07
117.19
77.21
77.00
76.78
52.70
29.68
230 220 210 200 190 180 170 160 150 140 130 120 110 100 90 80 70 60 50 40 30 20 10 0 -10
f1 (ppm)

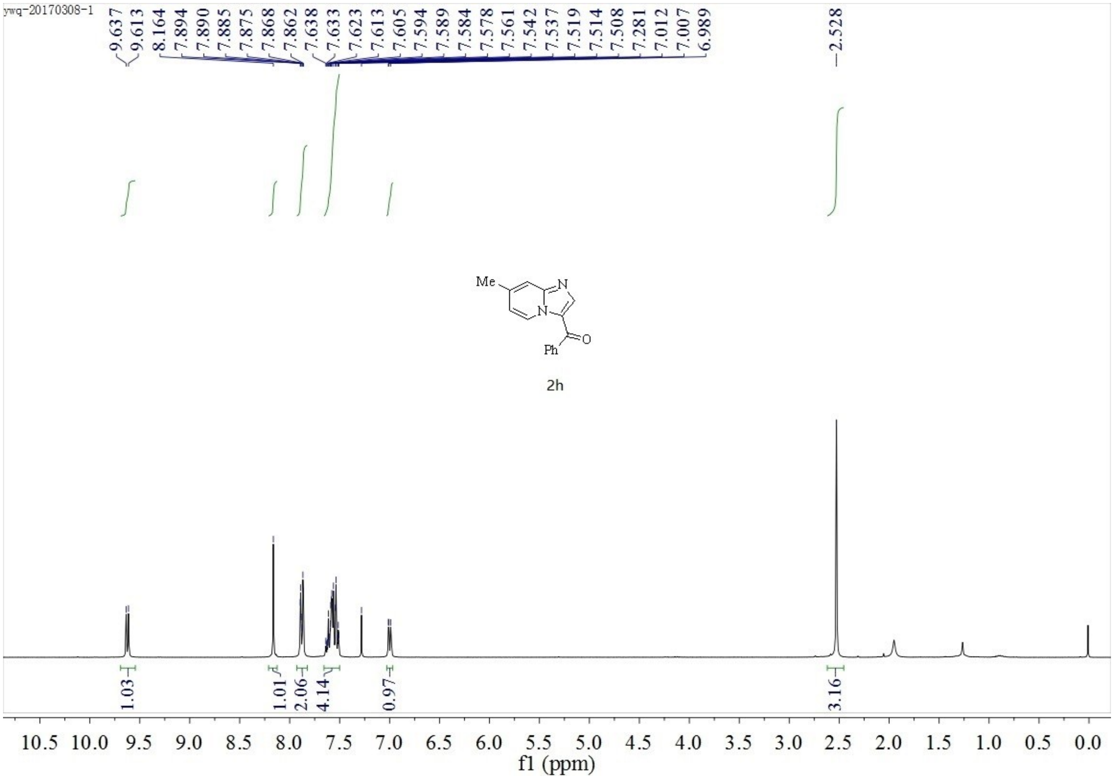

ywq-20170308-1
9.637
9.613
8.164
7.894
7.890
7.885
7.875
7.868
7.862
7.638
7.633
7.623
7.613
7.605
7.594
7.589
7.584
7.578
7.561
7.542
7.537
7.519
7.514
7.508
7.281
7.012
7.007
6.989
2.528
Me
N
N
O
Ph
2h
1.03
1.01
2.06
4.14
0.97
3.16
10.5 10.0 9.5 9.0 8.5 8.0 7.5 7.0 6.5 6.0 5.5 5.0 4.5 4.0 3.5 3.0 2.5 2.0 1.5 1.0 0.5 0.0
f1 (ppm)

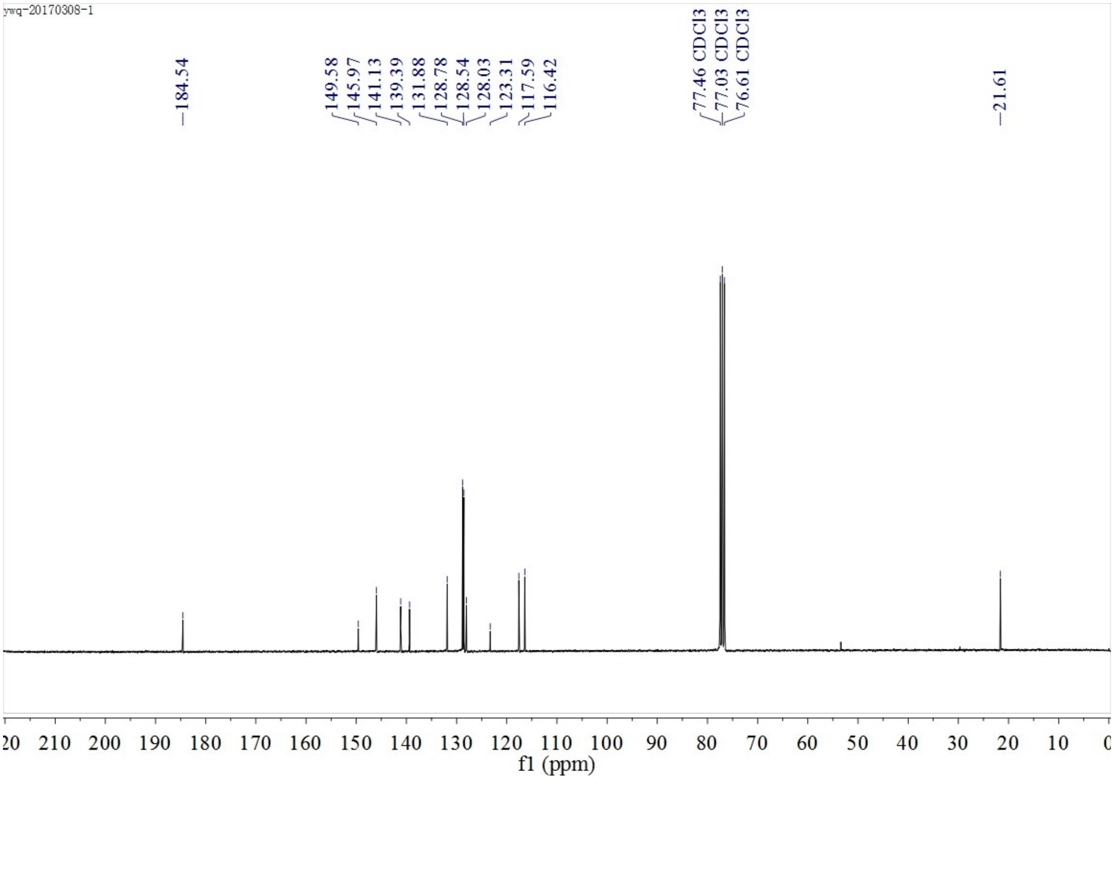

ywq-20170308-1
184.54
149.58
145.97
141.13
139.39
131.88
128.78
128.54
128.03
123.31
117.59
116.42
77.46 CDCl3
77.03 CDCl3
76.61 CDCl3
21.61
210 200 190 180 170 160 150 140 130 120 110 100 90 80 70 60 50 40 30 20 10
f1 (ppm)

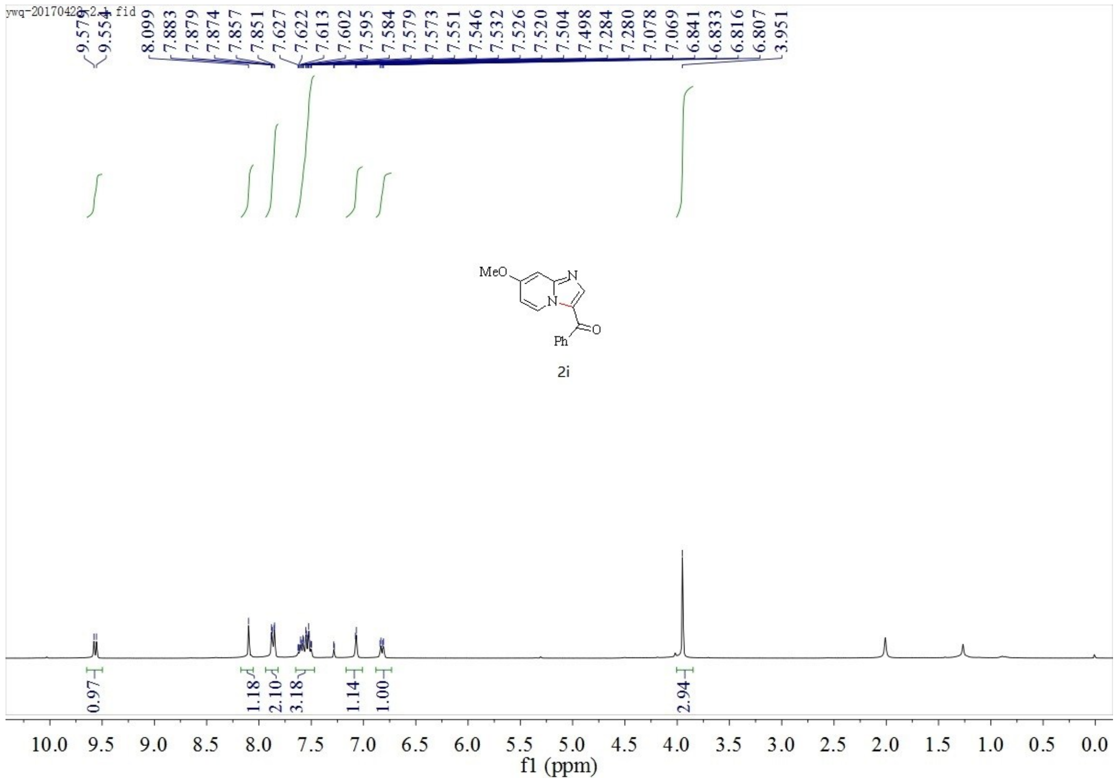
ywq-20170423-2.1.fid
9.579
9.554
8.099
7.883
7.879
7.874
7.857
7.851
7.627
7.622
7.613
7.602
7.595
7.584
7.579
7.573
7.551
7.546
7.532
7.526
7.520
7.504
7.498
7.284
7.280
7.078
7.069
6.841
6.833
6.816
6.807
3.951
MeO
N
N
O
Ph
2i
0.97
1.18
2.10
3.18
1.14
1.00
2.94
10.0 9.5 9.0 8.5 8.0 7.5 7.0 6.5 6.0 5.5 5.0 4.5 4.0 3.5 3.0 2.5 2.0 1.5 1.0 0.5 0.0
f1 (ppm)

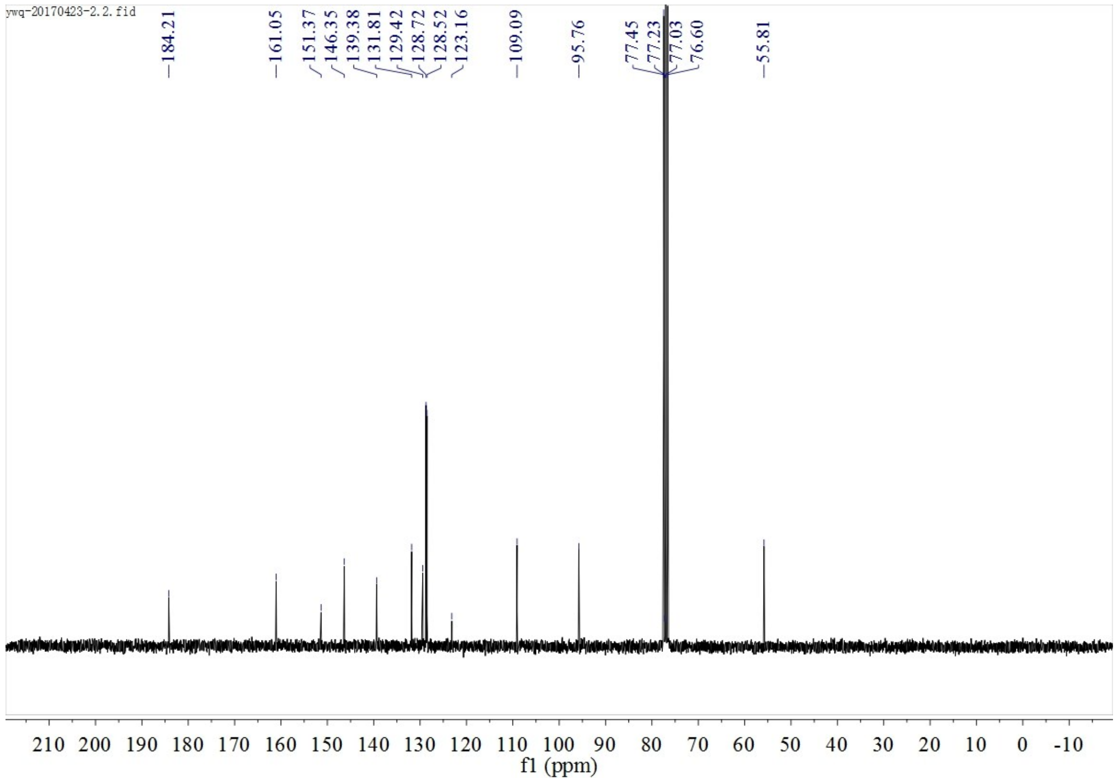
ywq-20170423-2.2.fid
184.21
161.05
151.37
146.35
139.38
131.81
129.42
128.72
128.52
123.16
109.09
95.76
77.45
77.23
77.03
76.60
55.81
210 200 190 180 170 160 150 140 130 120 110 100 90 80 70 60 50 40 30 20 10 0 -10
f1 (ppm)

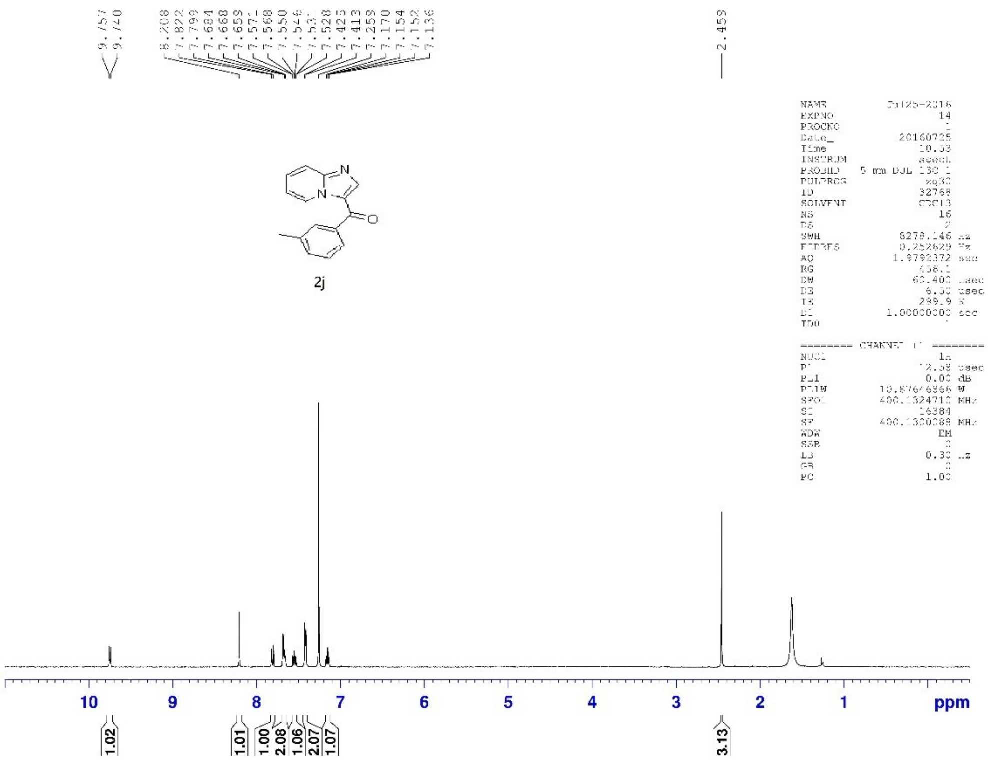
9.757
9.740
8.208
7.822
7.799
7.684
7.668
7.659
7.568
7.550
7.528
7.423
7.413
7.259
7.170
7.154
7.152
7.136
2.459
2j
10 9 8 7 6 5 4 3 2 1 ppm
1.02
1.01
1.00
2.08
1.06
2.07
1.07
3.13

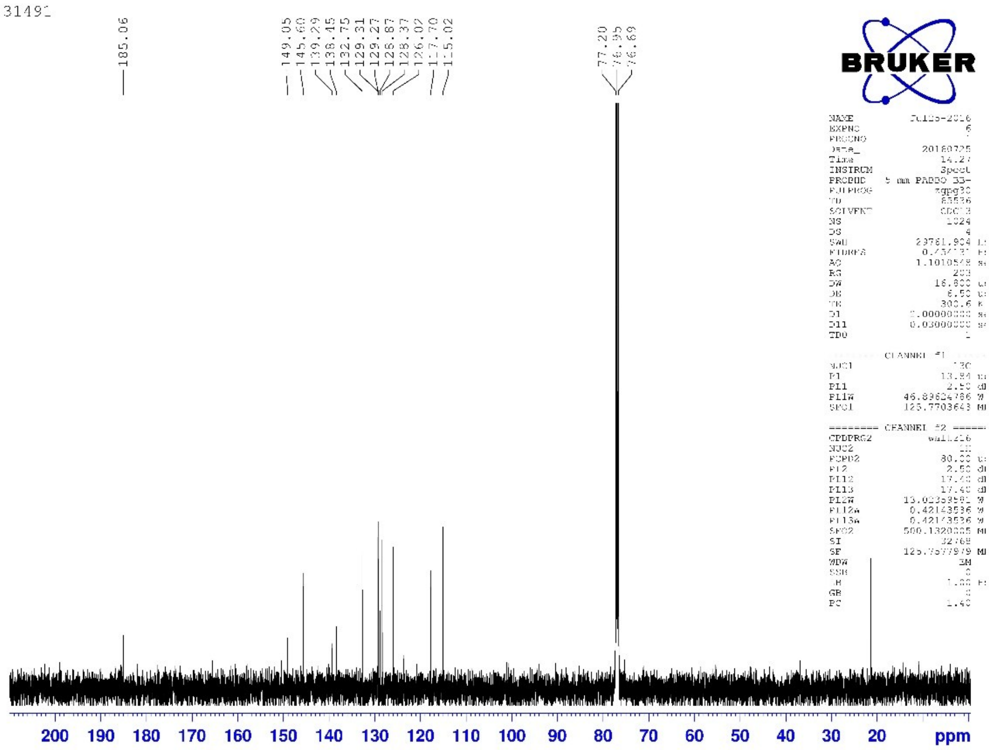
185.06
149.05
145.60
139.29
138.45
132.75
129.31
129.27
128.87
128.37
126.02
115.02
77.20
76.95
76.69
200 190 180 170 160 150 140 130 120 110 100 90 80 70 60 50 40 30 20 ppm

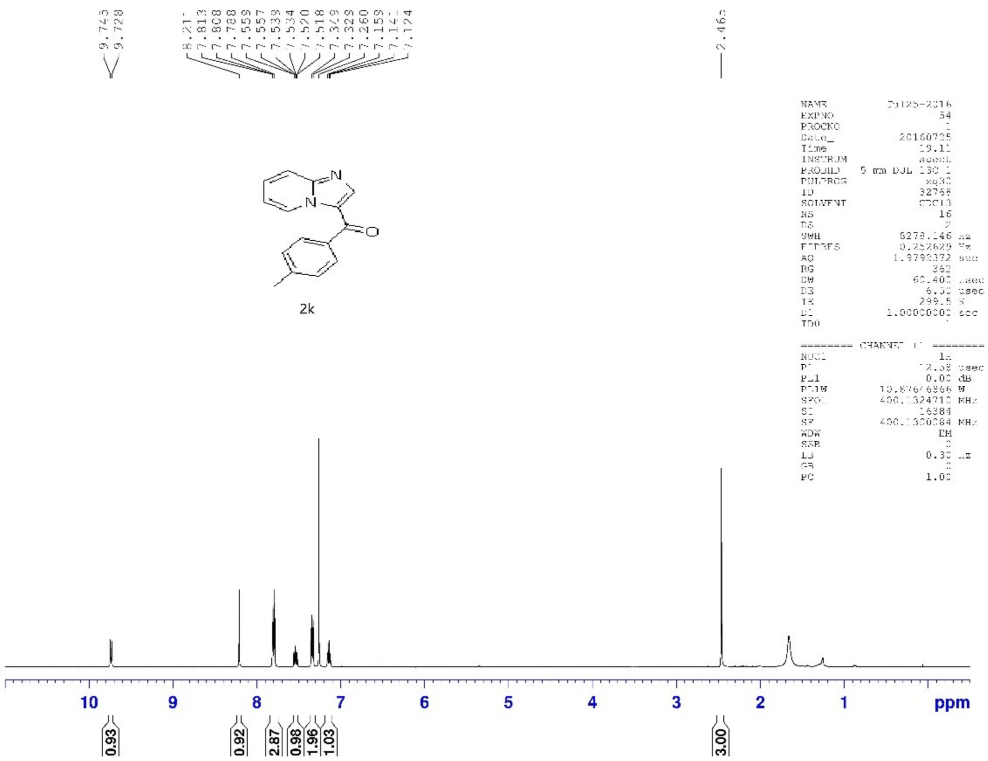

2k

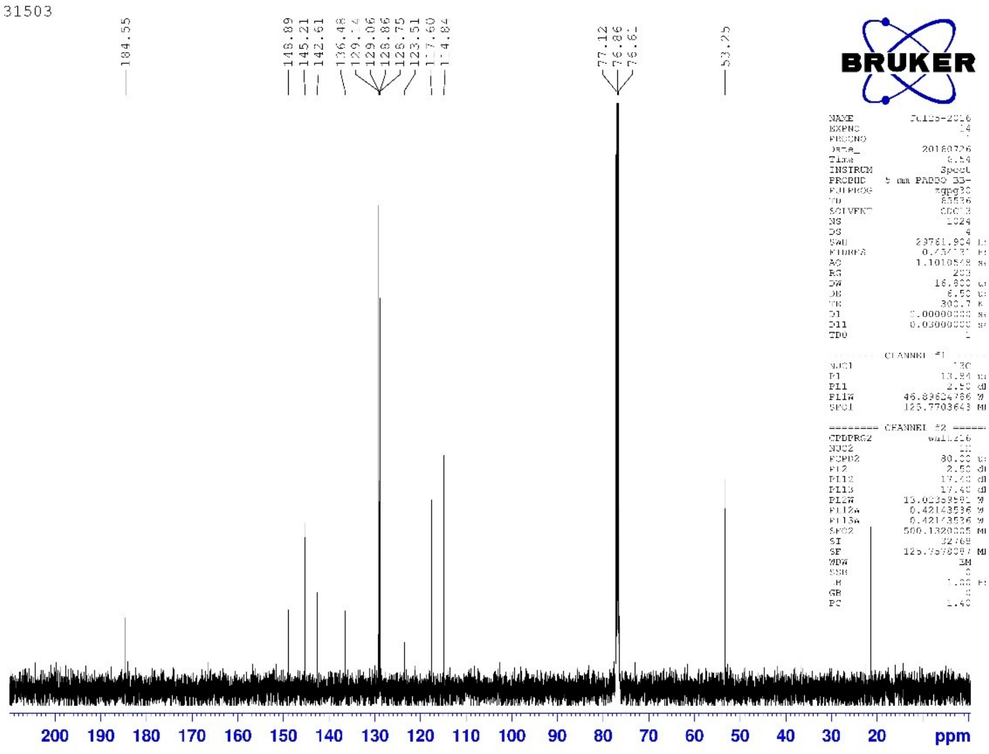

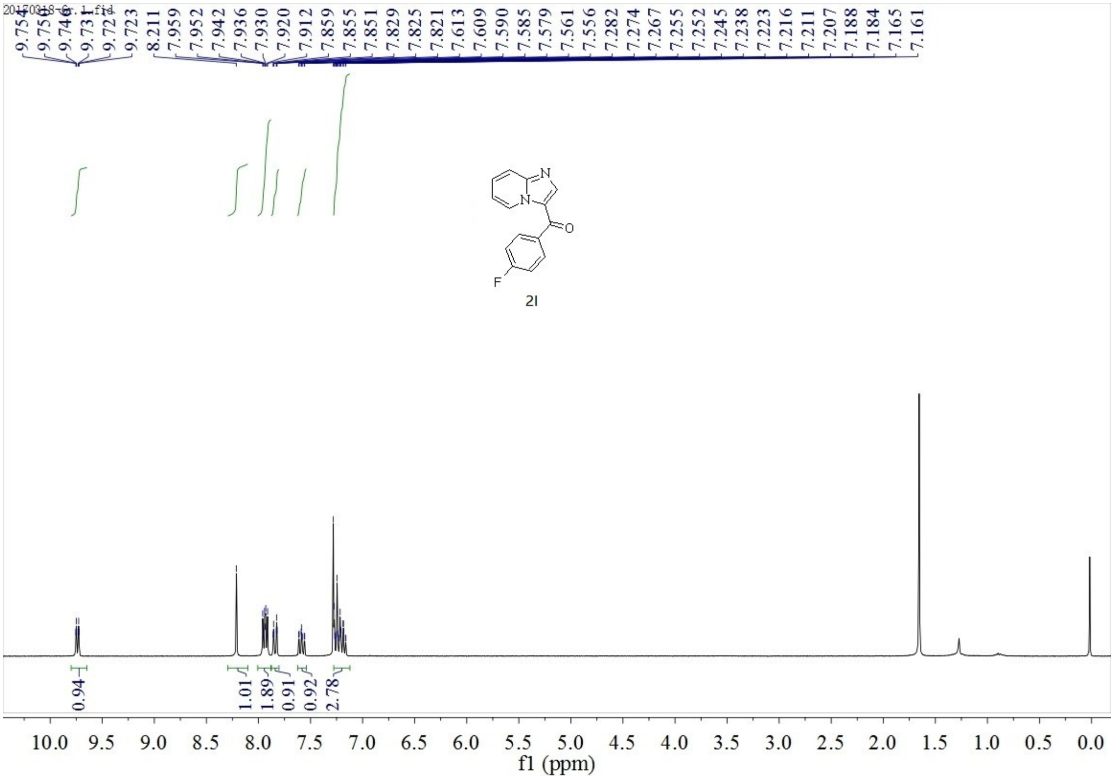
20170318-6r.1.fid
9.754
9.751
9.746
9.731
9.727
9.723
8.211
7.959
7.952
7.942
7.936
7.930
7.920
7.912
7.859
7.855
7.851
7.829
7.825
7.821
7.613
7.609
7.590
7.585
7.579
7.561
7.556
7.282
7.274
7.267
7.255
7.252
7.245
7.238
7.223
7.216
7.211
7.207
7.188
7.184
7.165
7.161
N
N
O
F
2l
0.94
1.01
1.89
0.91
0.92
2.78
10.0 9.5 9.0 8.5 8.0 7.5 7.0 6.5 6.0 5.5 5.0 4.5 4.0 3.5 3.0 2.5 2.0 1.5 1.0 0.5 0.0
f1 (ppm)

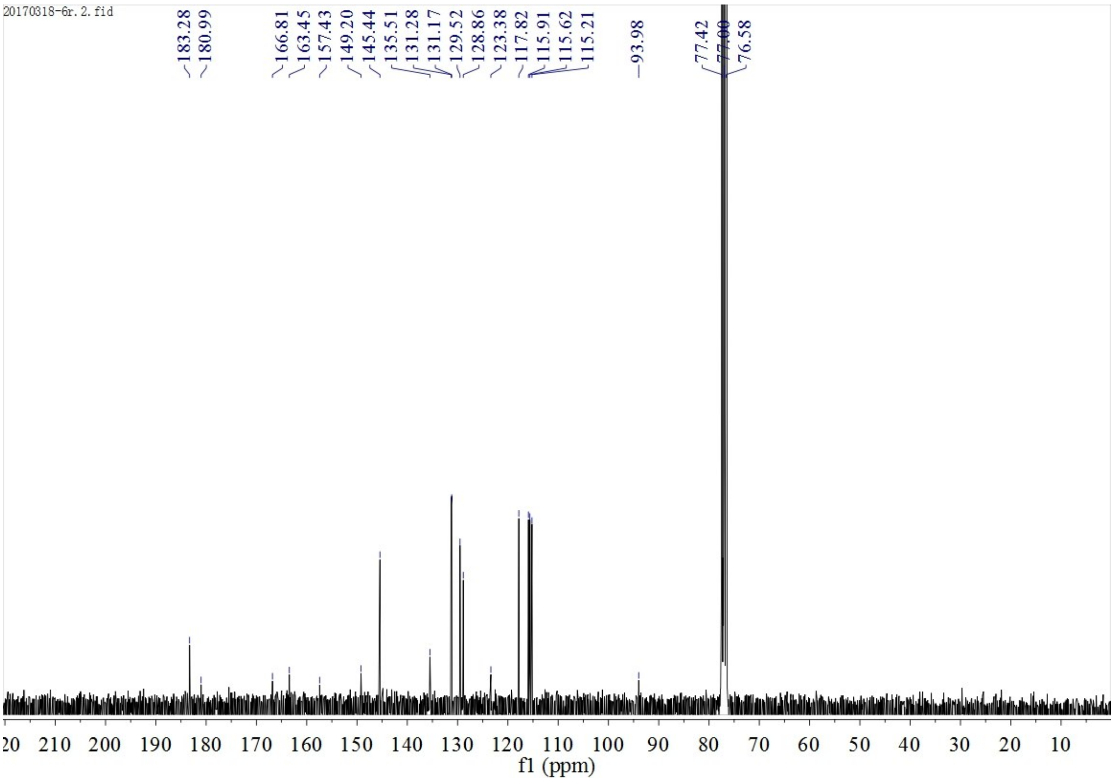
20170318-6r.2.fid
183.28
180.99
166.81
163.45
157.43
149.20
145.44
135.51
131.28
131.17
129.52
128.86
123.38
117.82
115.91
115.62
115.21
93.98
77.42
77.00
76.58
20 210 200 190 180 170 160 150 140 130 120 110 100 90 80 70 60 50 40 30 20 10
f1 (ppm)

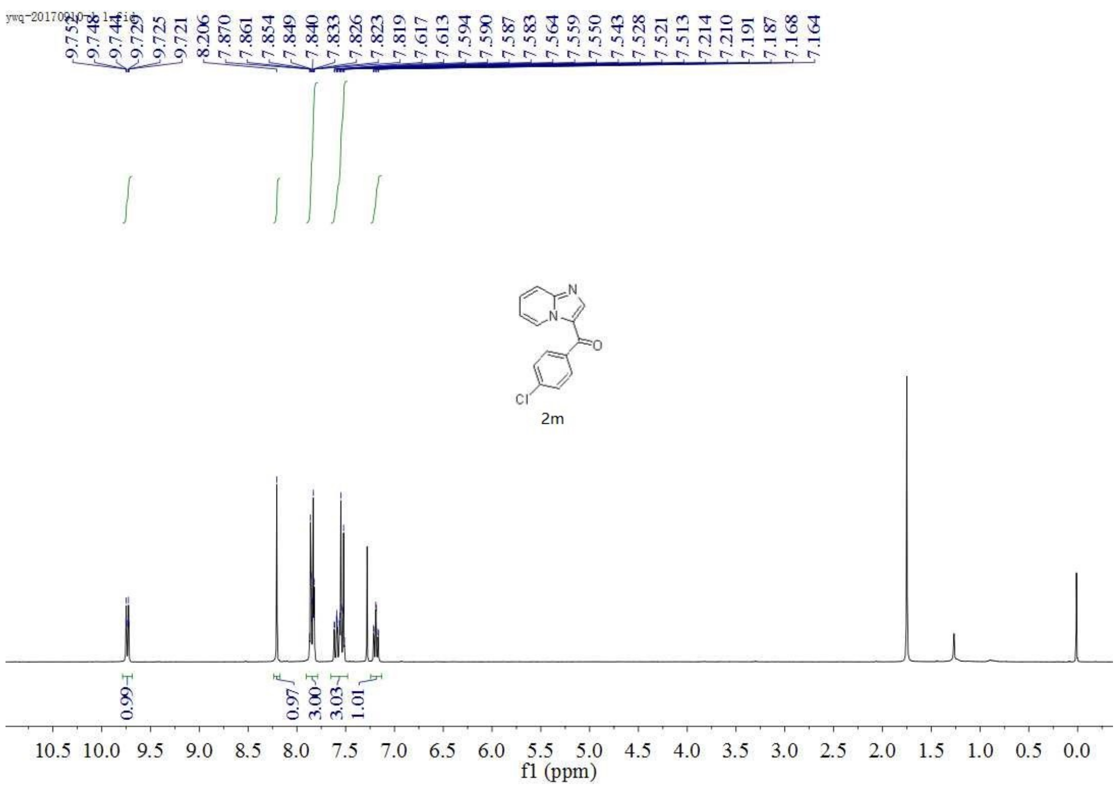
9.748
9.744
9.729
9.725
9.721
8.206
7.870
7.861
7.854
7.849
7.840
7.833
7.826
7.823
7.819
7.617
7.613
7.594
7.590
7.587
7.583
7.564
7.559
7.550
7.543
7.528
7.521
7.513
7.214
7.210
7.191
7.187
7.168
7.164
N
O
Cl
2m
0.99
0.97
3.00
3.03
1.01
10.5 10.0 9.5 9.0 8.5 8.0 7.5 7.0 6.5 6.0 5.5 5.0 4.5 4.0 3.5 3.0 2.5 2.0 1.5 1.0 0.5 0.0
f1 (ppm)

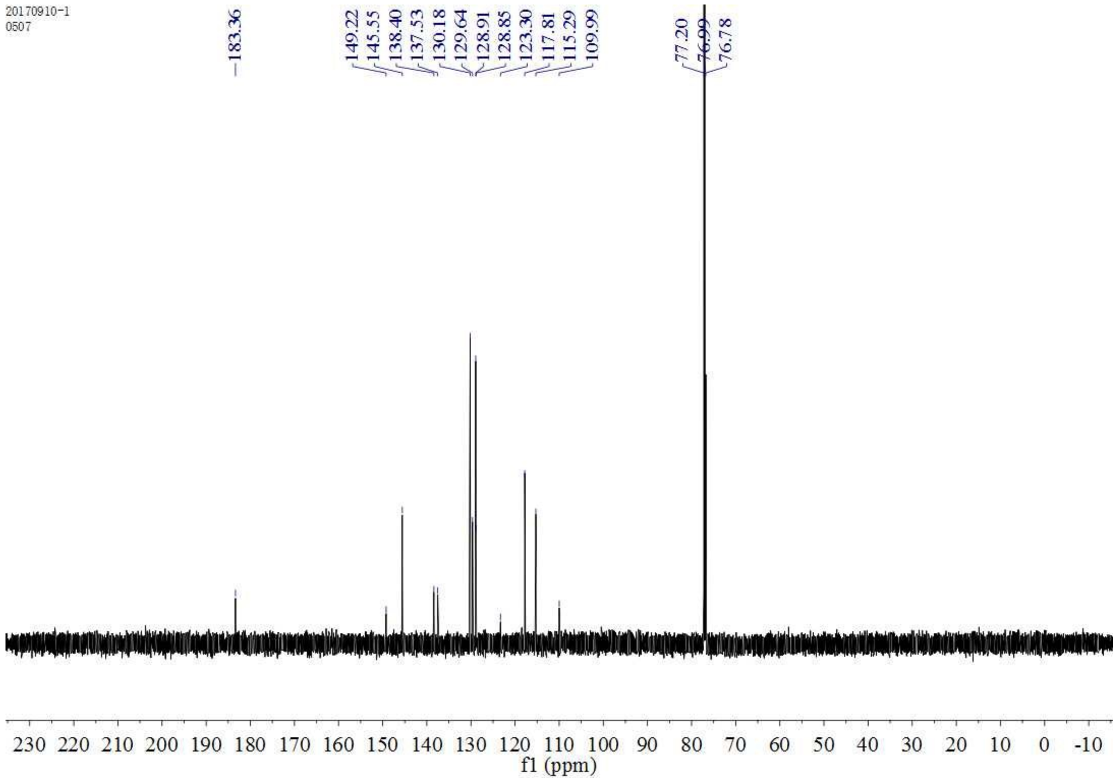
20170910-1
0507
183.36
149.22
145.55
138.40
137.53
130.18
129.64
128.91
128.85
123.30
117.81
115.29
109.99
77.20
76.99
76.78
230 220 210 200 190 180 170 160 150 140 130 120 110 100 90 80 70 60 50 40 30 20 10 0 -10
f1 (ppm)

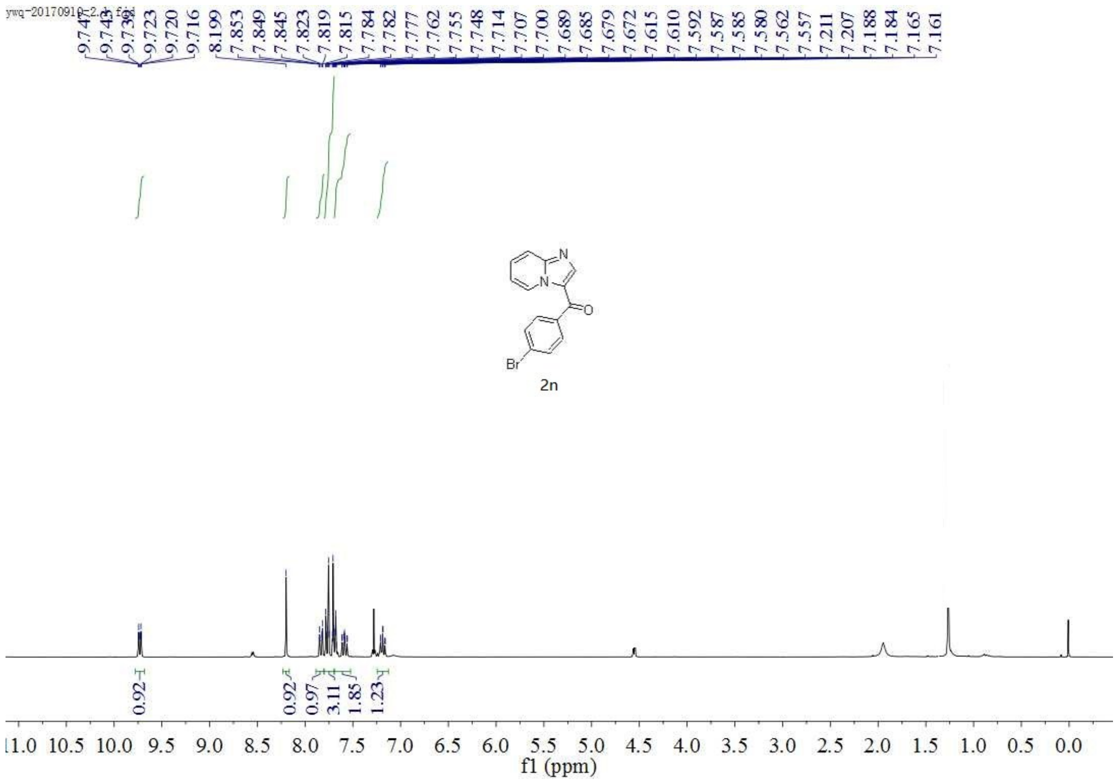
ywq-20170910-2.1.fid
9.741
9.743
9.739
9.723
9.720
9.716
8.199
7.853
7.849
7.845
7.823
7.819
7.815
7.784
7.782
7.777
7.762
7.755
7.748
7.714
7.707
7.700
7.689
7.685
7.679
7.672
7.615
7.610
7.592
7.587
7.585
7.580
7.562
7.557
7.211
7.207
7.188
7.184
7.165
7.161
N
N
O
Br
2n
0.92
0.92
0.97
3.11
1.85
1.23
11.0 10.5 10.0 9.5 9.0 8.5 8.0 7.5 7.0 6.5 6.0 5.5 5.0 4.5 4.0 3.5 3.0 2.5 2.0 1.5 1.0 0.5 0.0
f1 (ppm)

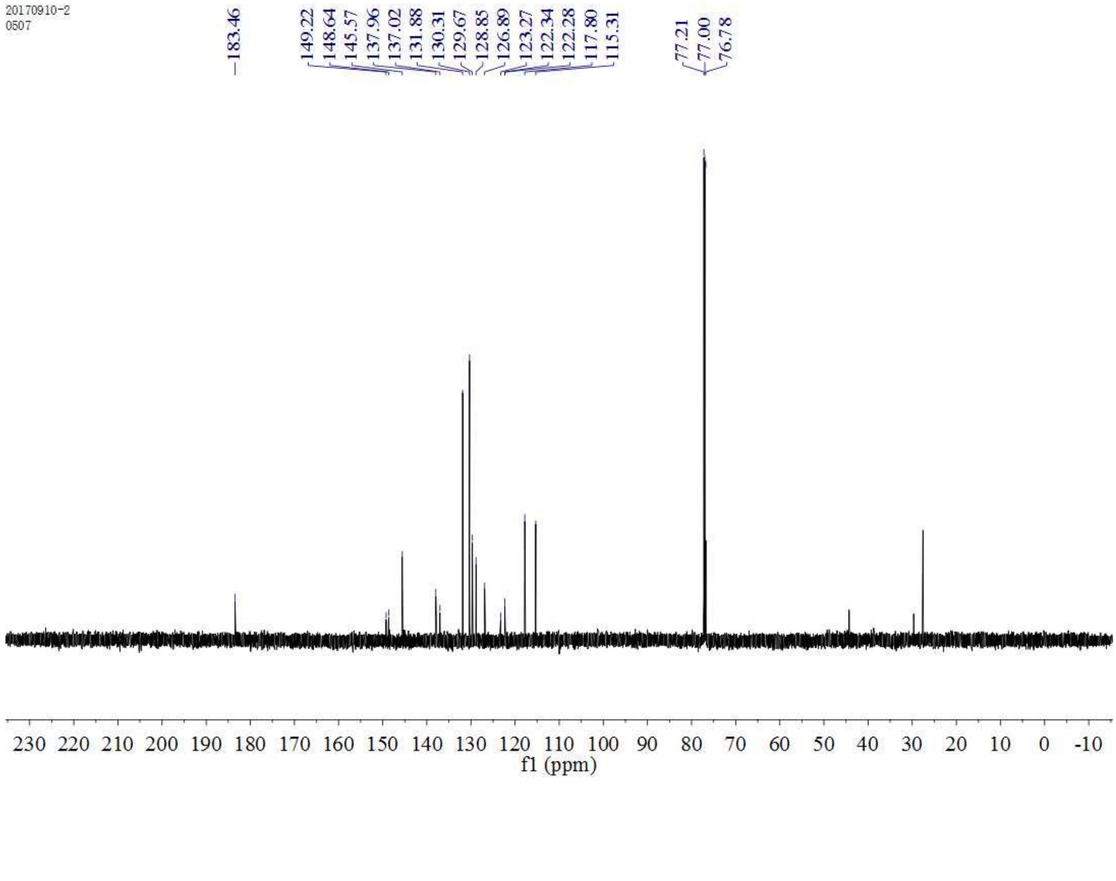
20170910-2
0507
183.46
149.22
148.64
145.57
137.96
137.02
131.88
130.31
129.67
128.85
126.89
123.27
122.34
122.28
117.80
115.31
77.21
77.00
76.78
230 220 210 200 190 180 170 160 150 140 130 120 110 100 90 80 70 60 50 40 30 20 10 0 -10
f1 (ppm)

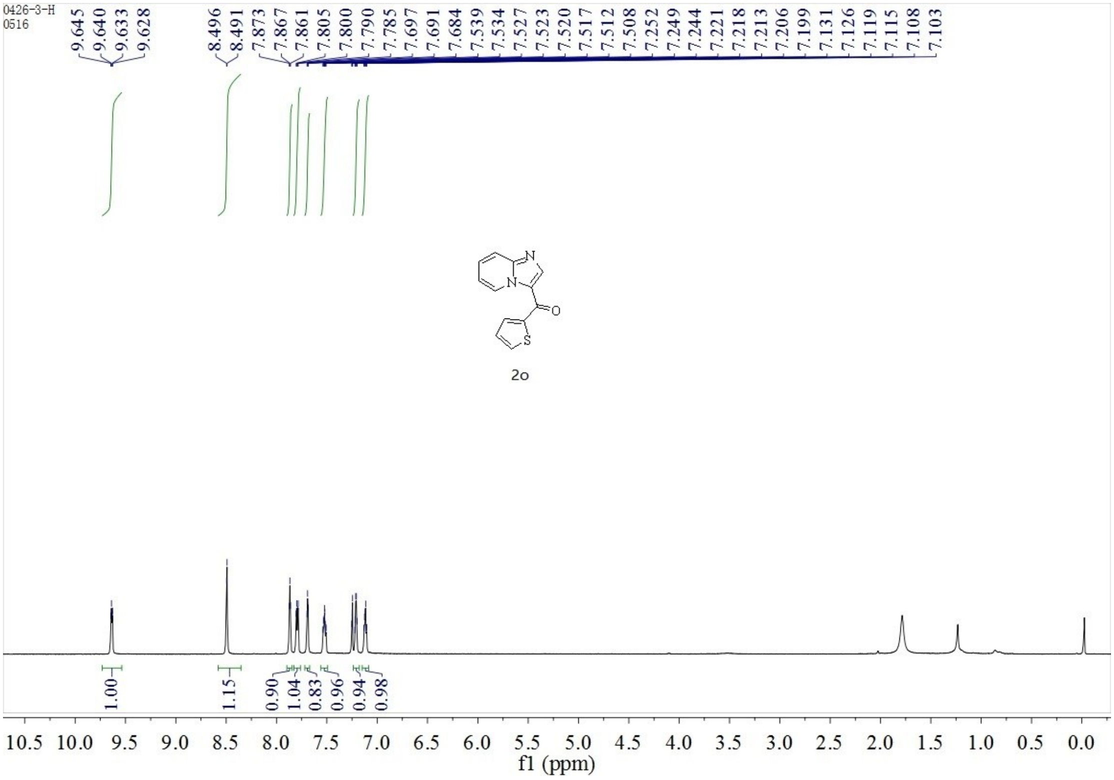
0426-3-H
0516
9.645 9.640 9.633 9.628 8.496 8.491 7.873 7.867 7.861 7.805 7.800 7.790 7.785 7.697 7.691 7.684 7.539 7.534 7.527 7.523 7.520 7.517 7.512 7.508 7.252 7.249 7.244 7.221 7.218 7.213 7.206 7.199 7.131 7.126 7.119 7.115 7.108 7.103
N
N
O
S
2o
1.00 1.15 0.90 1.04 0.83 0.96 0.94 0.98
10.5 10.0 9.5 9.0 8.5 8.0 7.5 7.0 6.5 6.0 5.5 5.0 4.5 4.0 3.5 3.0 2.5 2.0 1.5 1.0 0.5 0.0
f1 (ppm)

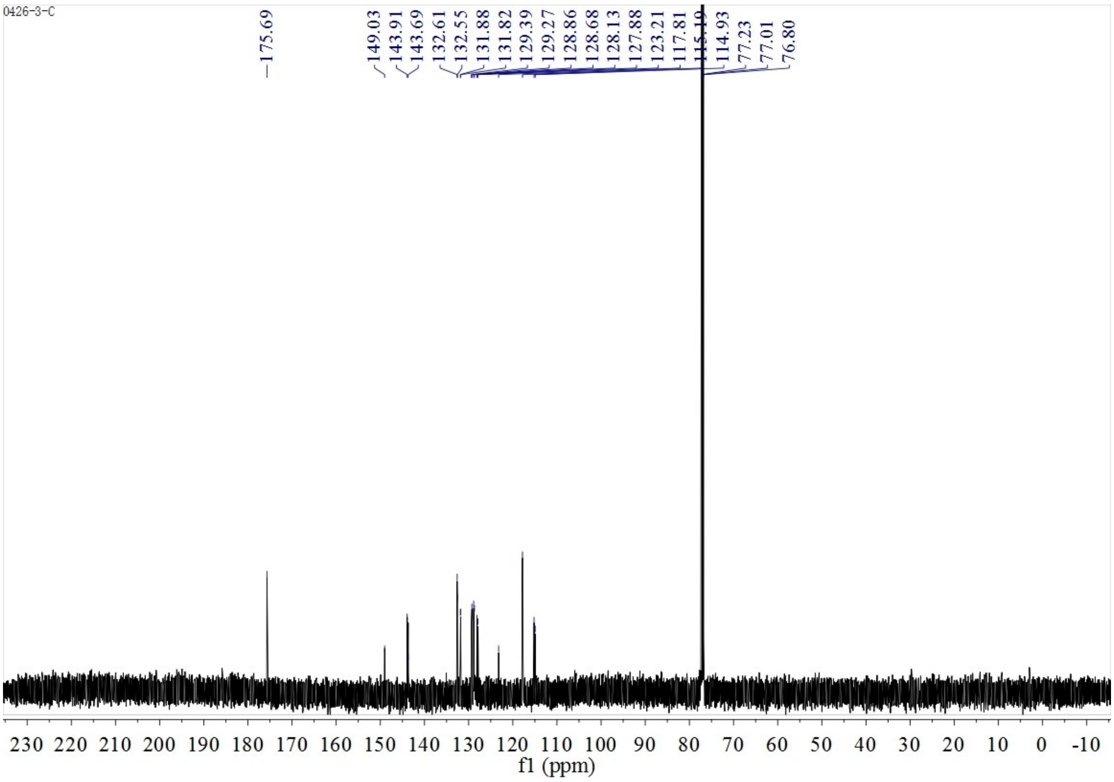
0426-3-C
175.69
149.03 143.91 143.69 132.61 132.55 131.88 131.82 129.39 129.27 128.86 128.68 128.13 127.88 123.21 117.81 115.10 114.93 77.23 77.01 76.80
230 220 210 200 190 180 170 160 150 140 130 120 110 100 90 80 70 60 50 40 30 20 10 0 -10
f1 (ppm)

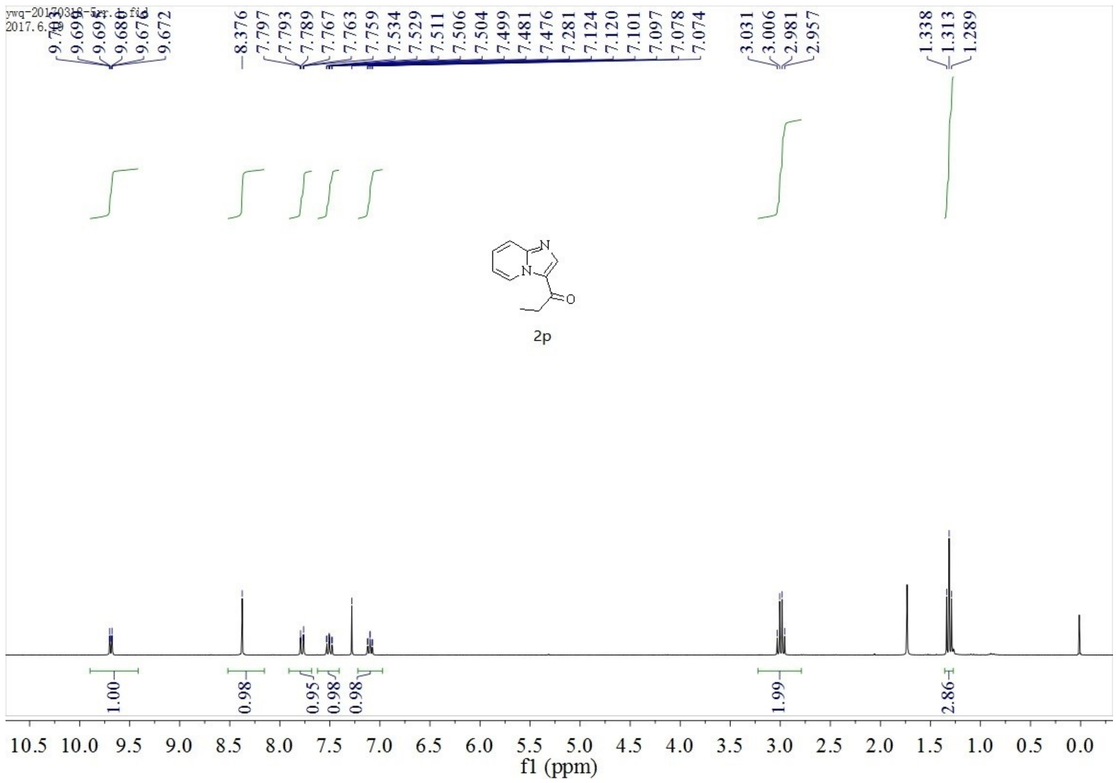

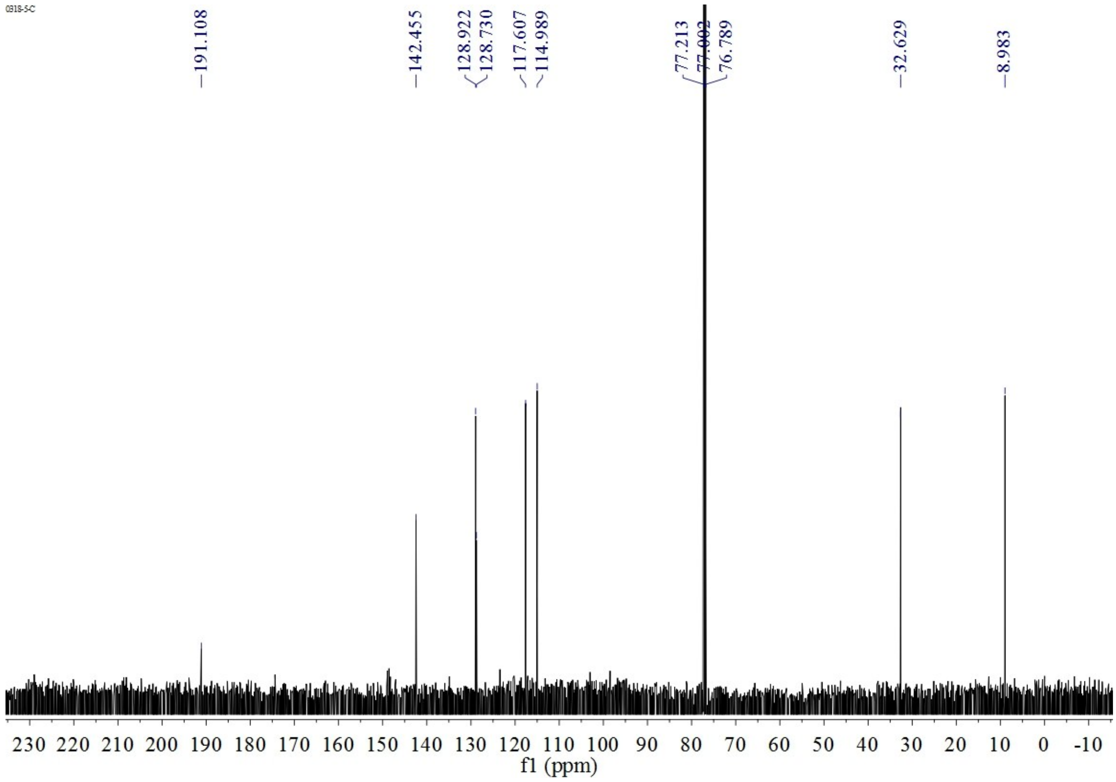

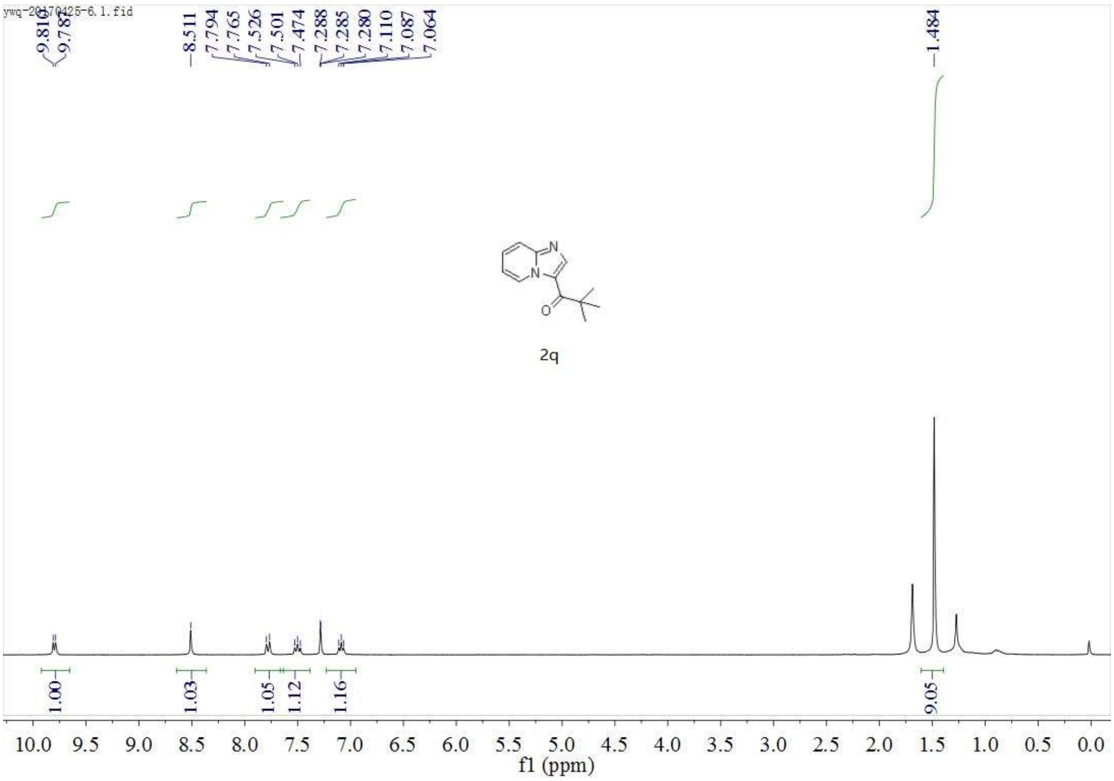
ywq-20170425-6.1.fid
9.810
9.787
8.511
7.794
7.765
7.526
7.501
7.474
7.288
7.285
7.280
7.110
7.087
7.064
1.484
N
N
O
2q
1.00
1.03
1.05
1.12
1.16
9.05
10.0 9.5 9.0 8.5 8.0 7.5 7.0 6.5 6.0 5.5 5.0 4.5 4.0 3.5 3.0 2.5 2.0 1.5 1.0 0.5 0.0
f1 (ppm)

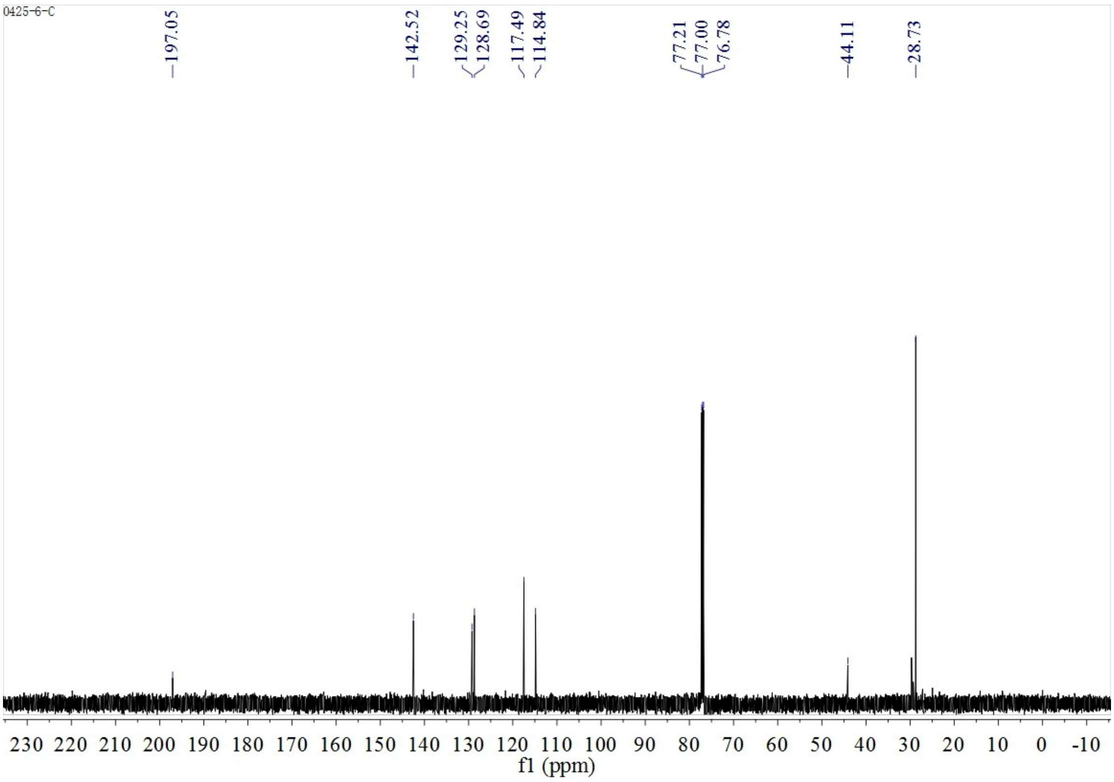
0425-6-C
197.05
142.52
129.25
128.69
117.49
114.84
77.21
77.00
76.78
44.11
28.73
230 220 210 200 190 180 170 160 150 140 130 120 110 100 90 80 70 60 50 40 30 20 10 0 -10
f1 (ppm)

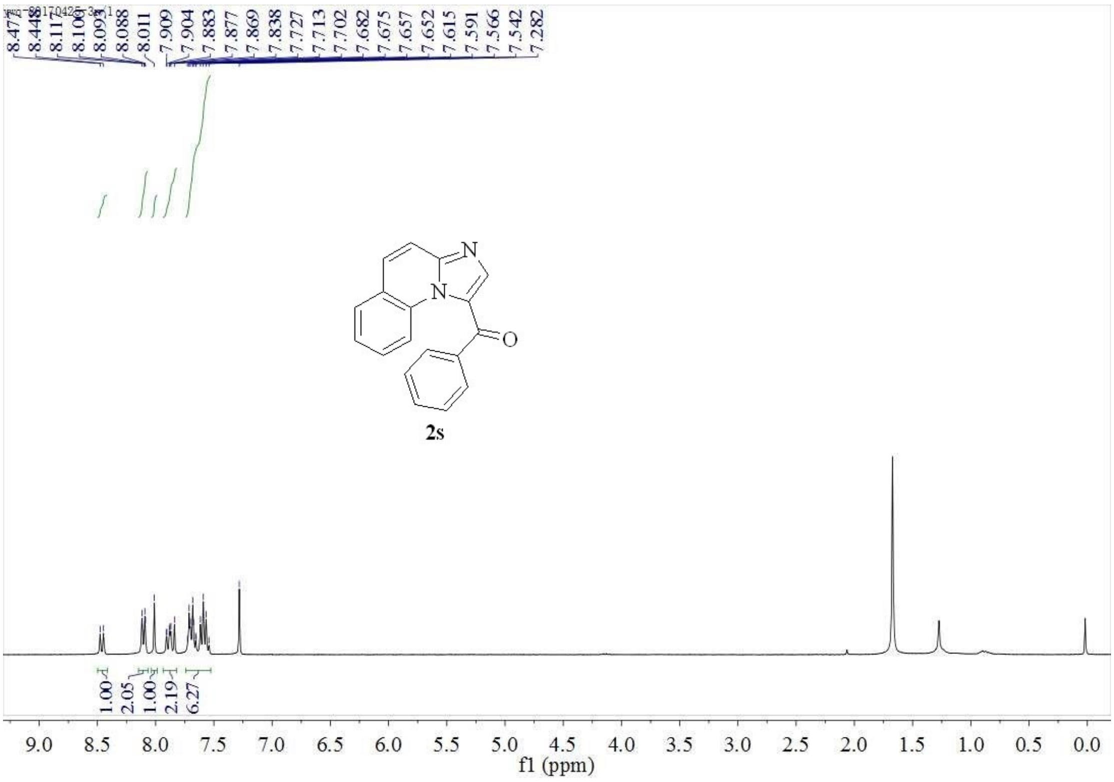

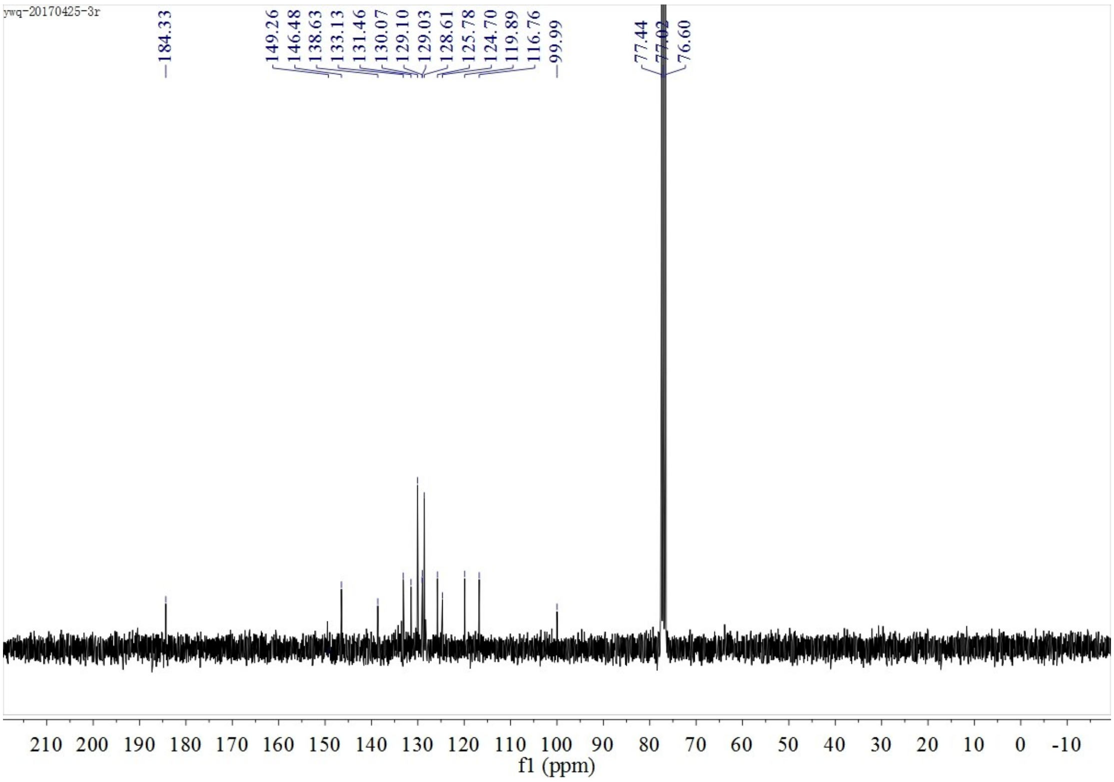

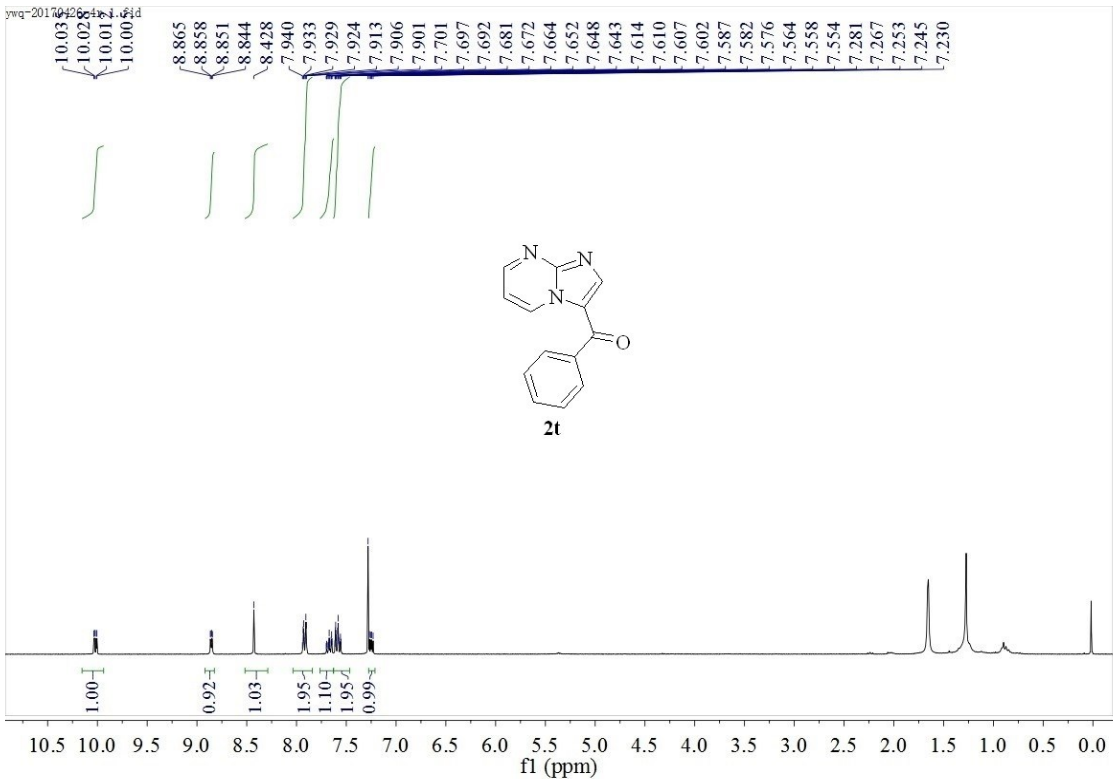
ywq-20170426-4r.1.fid
10.03 10.02 10.01 10.00 8.865 8.858 8.851 8.844 8.428 7.940 7.933 7.929 7.924 7.913 7.906 7.901 7.701 7.697 7.692 7.681 7.672 7.664 7.652 7.648 7.643 7.614 7.610 7.607 7.602 7.587 7.582 7.576 7.564 7.558 7.554 7.281 7.267 7.253 7.245 7.230
N
N
N
O
2t
1.00 0.92 1.03 1.95 1.10 1.95 0.99
10.5 10.0 9.5 9.0 8.5 8.0 7.5 7.0 6.5 6.0 5.5 5.0 4.5 4.0 3.5 3.0 2.5 2.0 1.5 1.0 0.5 0.0
f1 (ppm)

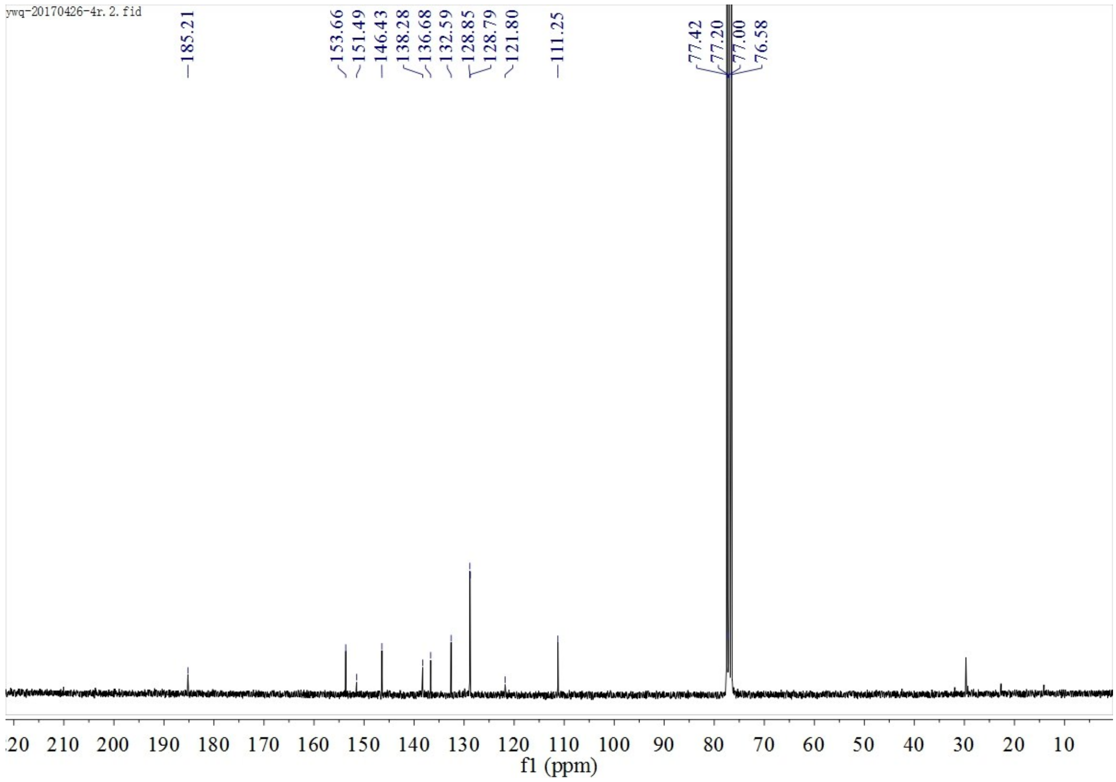
ywq-20170426-4r.2.fid
185.21
153.66 151.49 146.43 138.28 136.68 132.59 128.85 128.79 121.80
111.25
77.42 77.20 77.00 76.58
20 210 200 190 180 170 160 150 140 130 120 110 100 90 80 70 60 50 40 30 20 10
f1 (ppm)

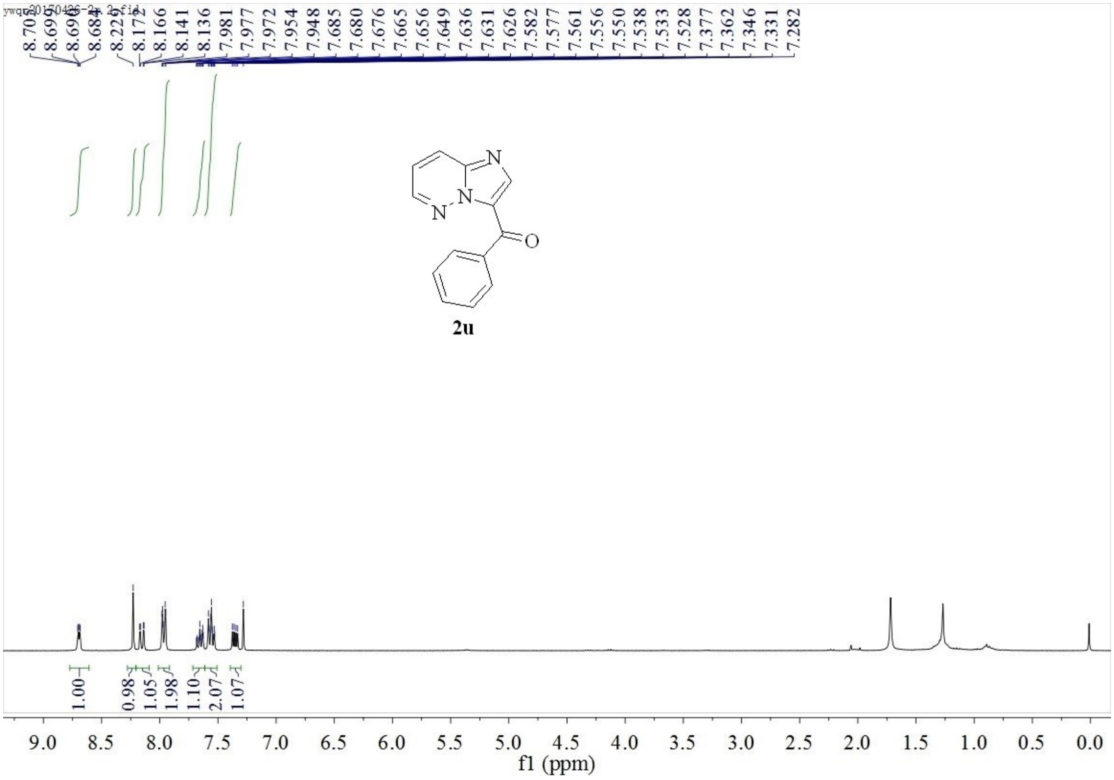
8.701
8.697
8.690
8.684
8.222
8.172
8.166
8.141
8.136
7.981
7.977
7.972
7.954
7.948
7.685
7.680
7.676
7.665
7.656
7.649
7.636
7.631
7.626
7.582
7.577
7.561
7.556
7.550
7.538
7.533
7.528
7.377
7.362
7.346
7.331
7.282
N
N
N
O
2u
1.00
0.98
1.05
1.98
1.10
2.07
1.07
f1 (ppm)

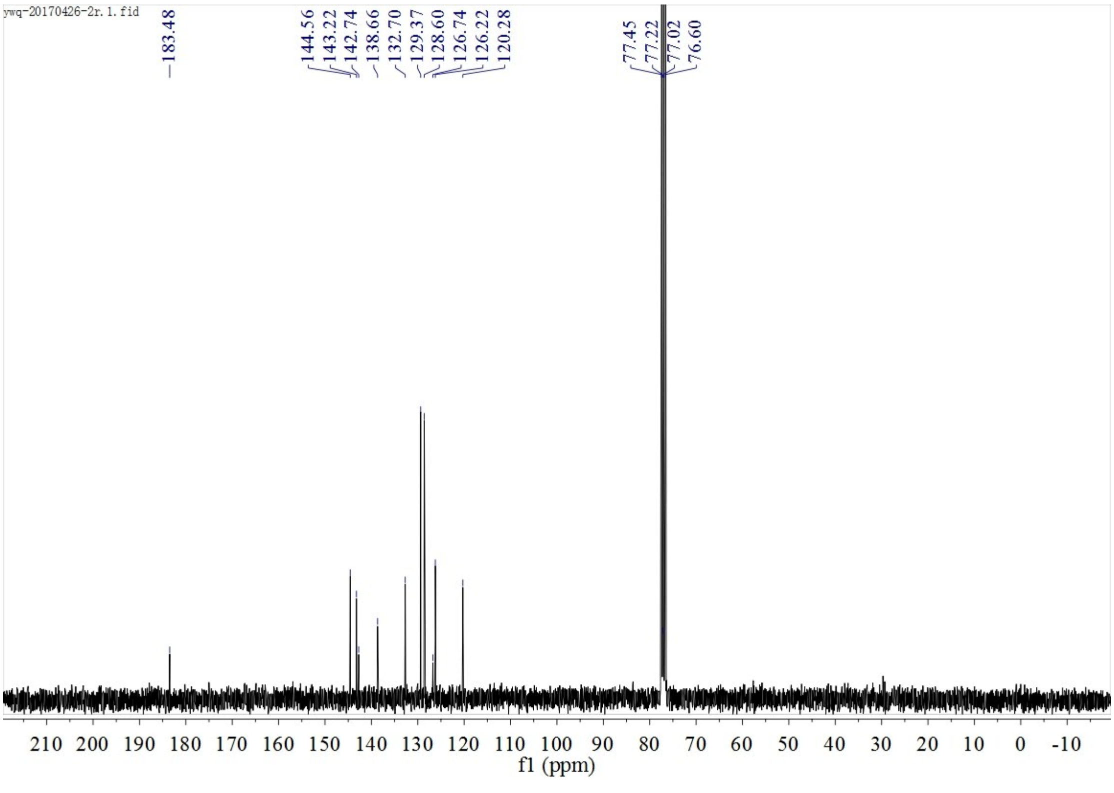
ywq-20170426-2r.1.fid
183.48
144.56
143.22
142.74
138.66
132.70
129.37
128.60
126.74
126.22
120.28
77.45
77.22
77.02
76.60
f1 (ppm)

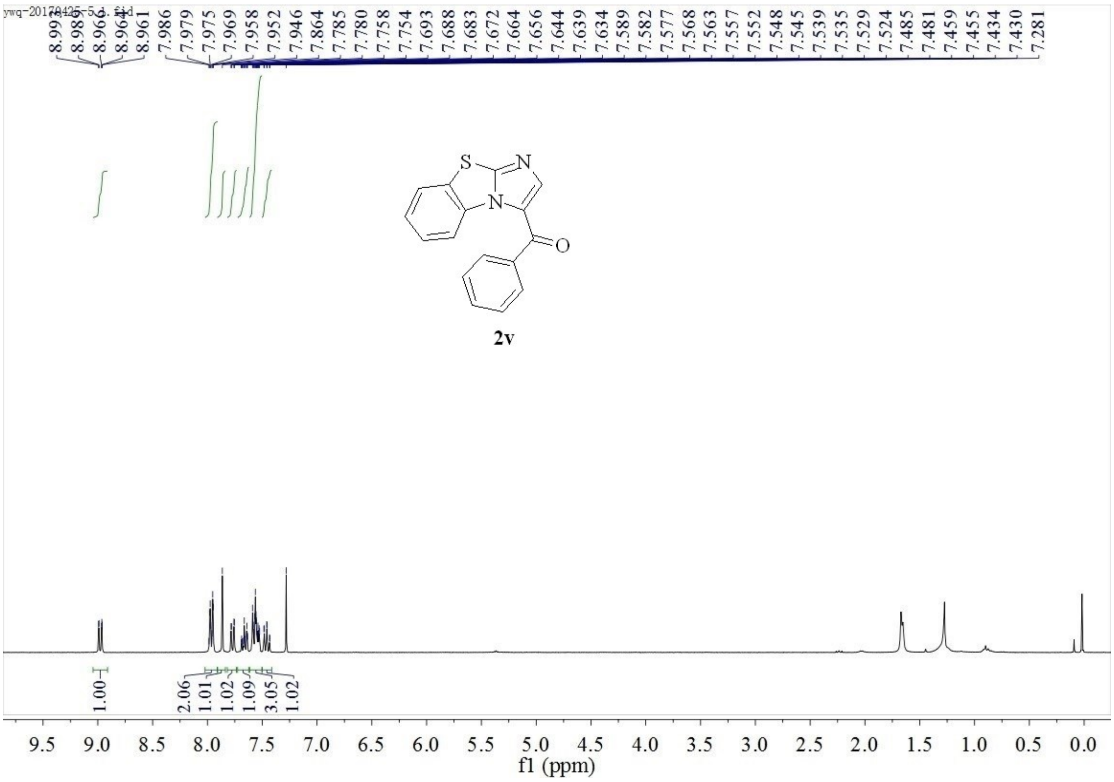
8.991
8.985
8.966
8.964
8.961
7.986
7.979
7.975
7.969
7.958
7.952
7.946
7.864
7.785
7.780
7.758
7.754
7.693
7.688
7.683
7.672
7.664
7.656
7.644
7.639
7.634
7.589
7.582
7.577
7.568
7.563
7.557
7.552
7.548
7.545
7.539
7.535
7.529
7.524
7.485
7.481
7.459
7.455
7.434
7.430
7.281
S
N
N
O
2v
1.00
2.06
1.01
1.02
1.09
3.05
1.02
9.5 9.0 8.5 8.0 7.5 7.0 6.5 6.0 5.5 5.0 4.5 4.0 3.5 3.0 2.5 2.0 1.5 1.0 0.5 0.0
f1 (ppm)

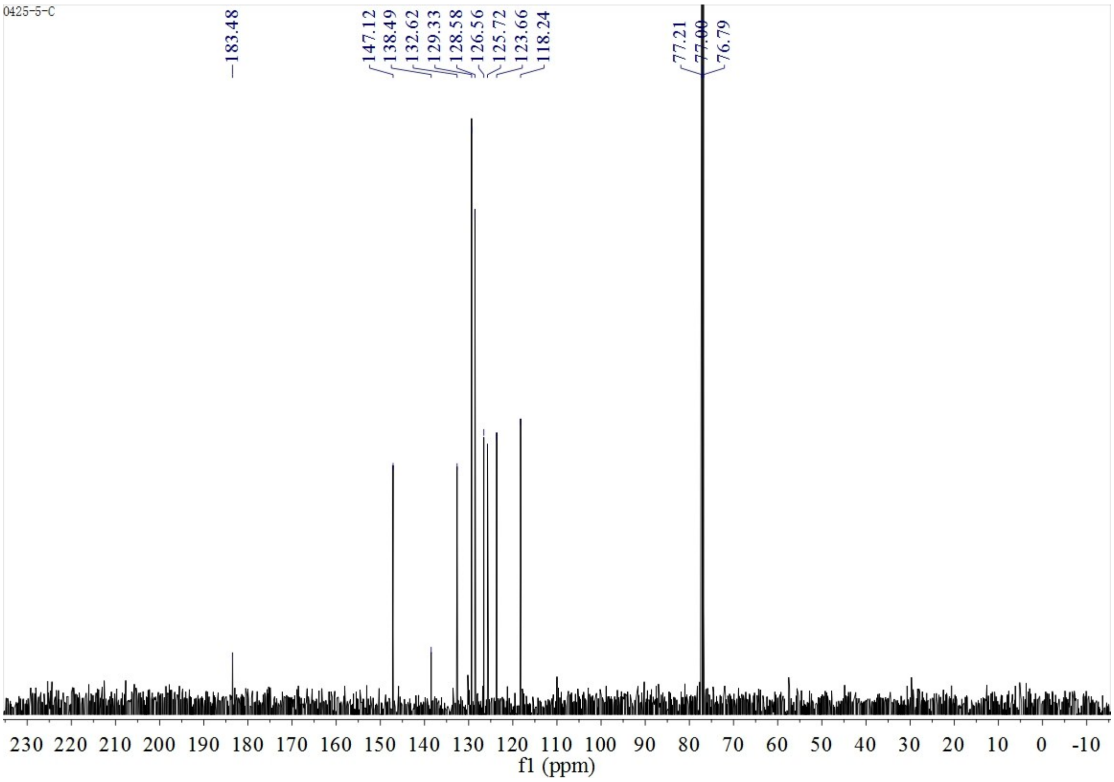
0425-5-C
183.48
147.12
138.49
132.62
129.33
128.58
126.56
125.72
123.66
118.24
77.21
77.00
76.79
230 220 210 200 190 180 170 160 150 140 130 120 110 100 90 80 70 60 50 40 30 20 10 0 -10
f1 (ppm)

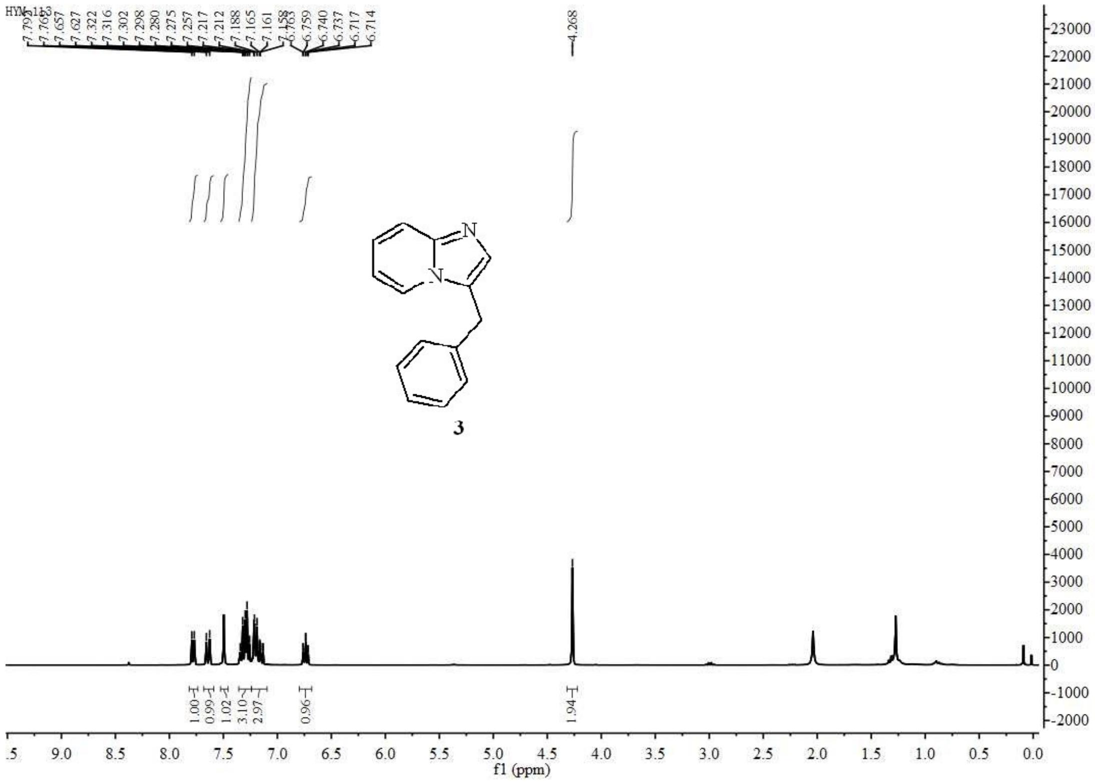
HYM-113
7.793 7.769 7.657 7.627 7.322 7.316 7.302 7.298 7.280 7.275 7.257 7.217 7.212 7.188 7.165 7.161 7.158 6.763 6.759 6.740 6.737 6.717 6.714
4.268
N
N
3
1.00 0.99 1.02 3.10 2.97 0.96 1.94
f1 (ppm)

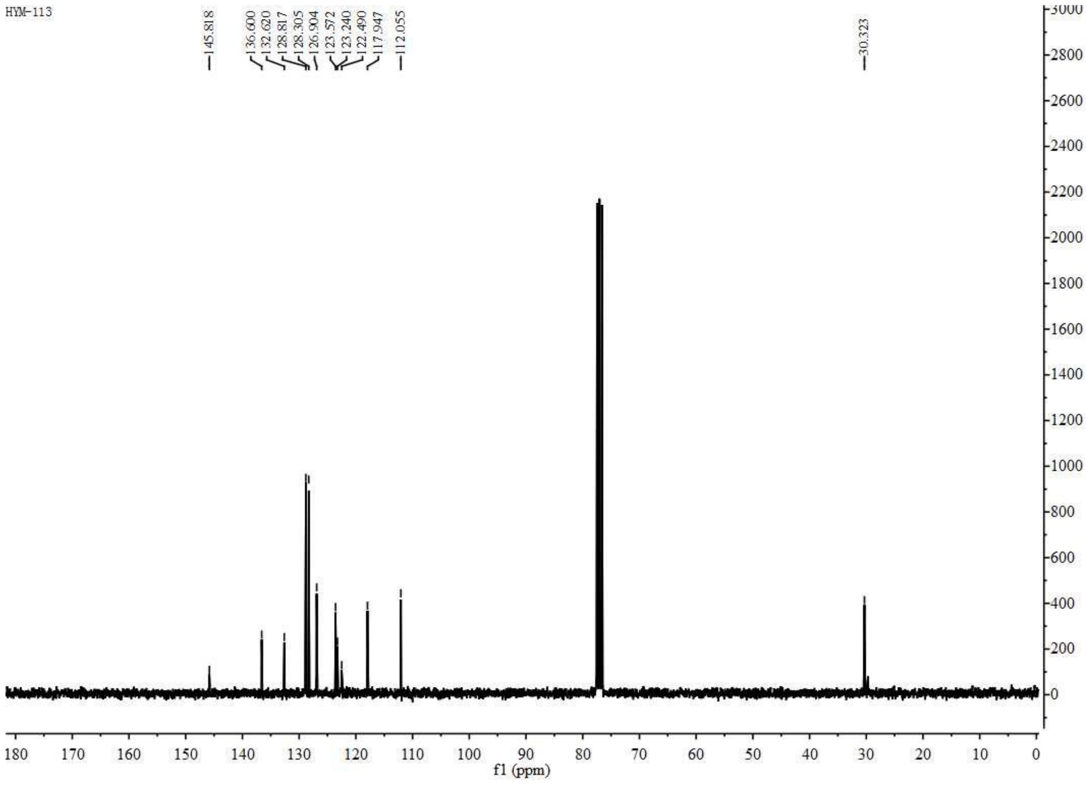
HYM-113
145.818
136.600 132.620 128.817 128.305 126.904 123.572 123.240 122.490 117.947 112.055
30.323
f1 (ppm)

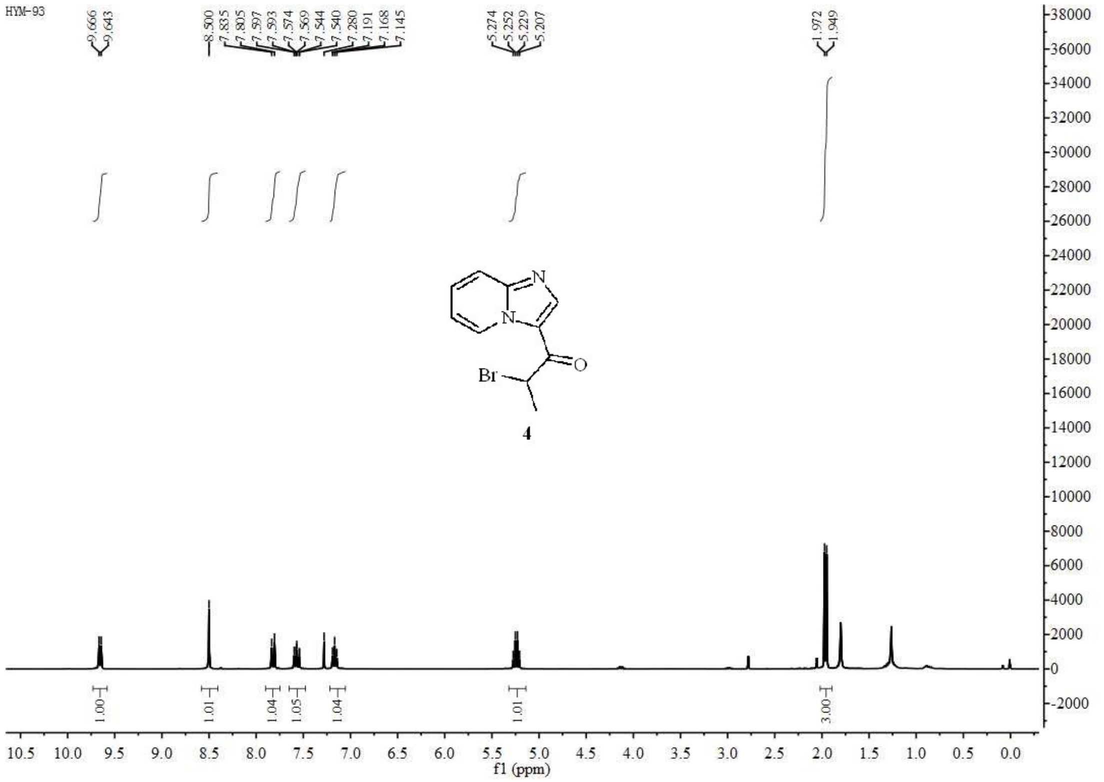
HYM-93
9.666
9.643
8.500
7.835
7.805
7.597
7.593
7.574
7.569
7.544
7.540
7.280
7.191
7.168
7.145
5.274
5.252
5.229
5.207
1.972
1.949
1.00
1.01
1.04
1.05
1.04
1.01
3.00
N
N
O
Br
4
f1 (ppm)

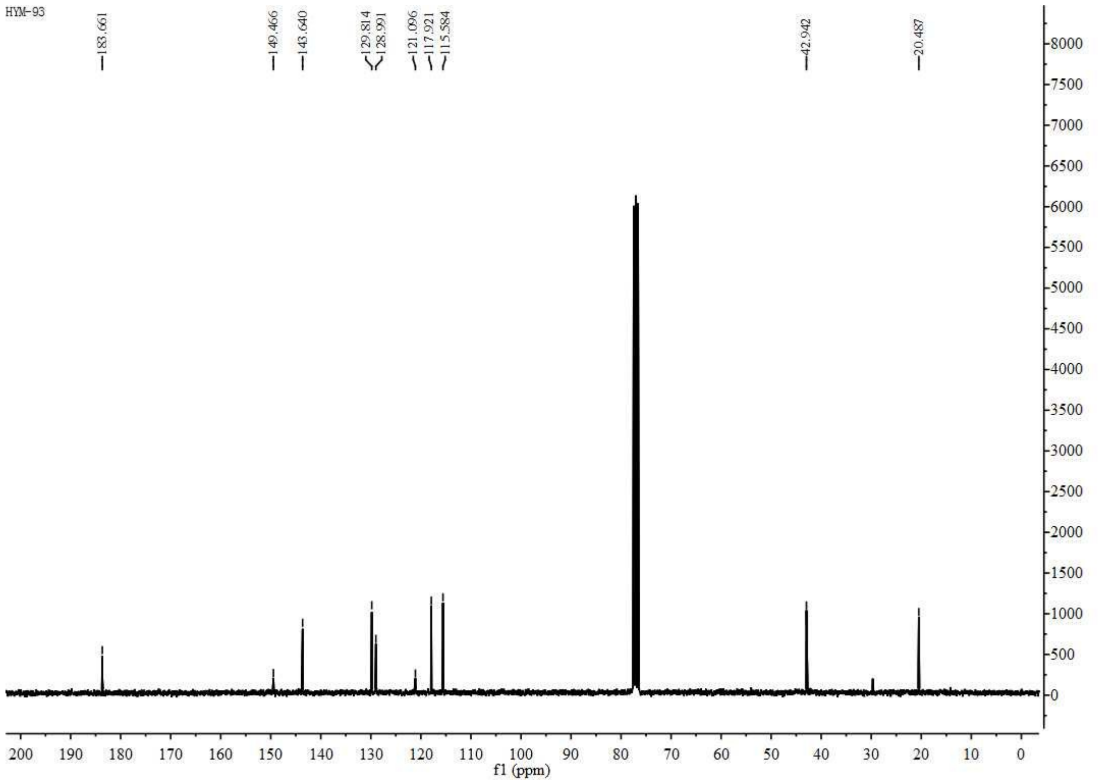
HYM-93
183.661
149.466
143.640
129.814
128.991
121.096
117.921
115.584
42.942
20.487
f1 (ppm)

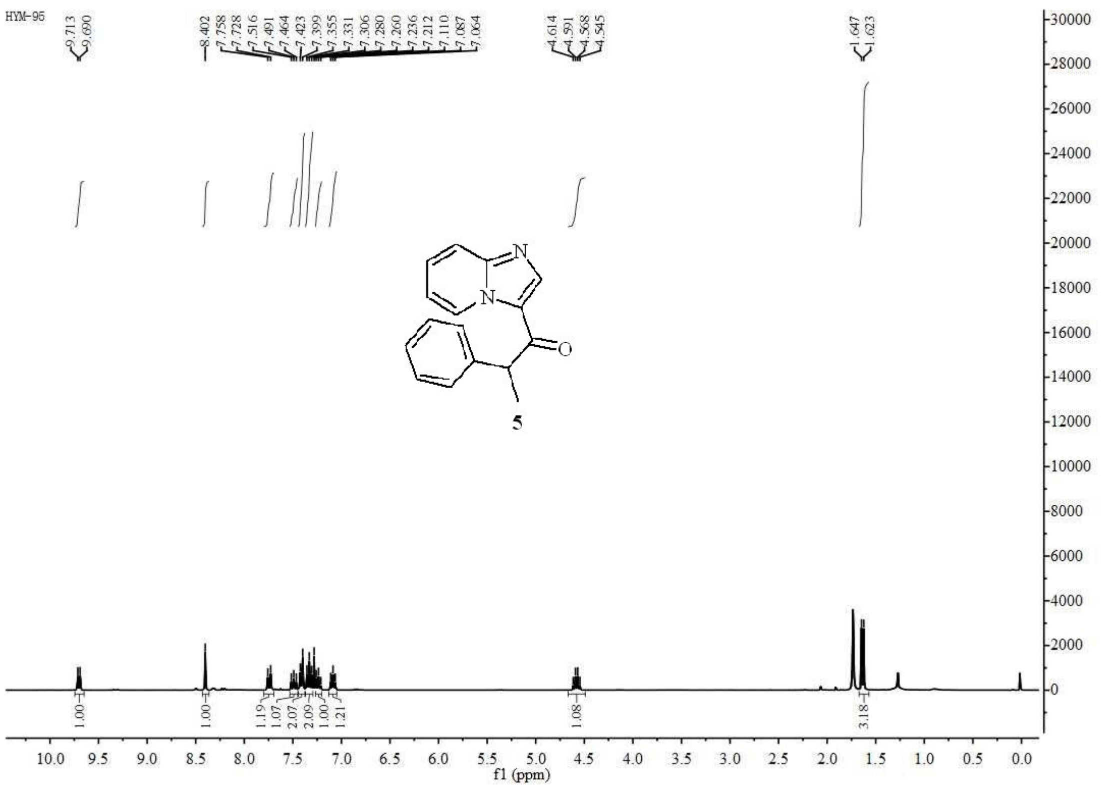
HYM-95
9.713
9.690
8.402
7.758
7.728
7.516
7.491
7.464
7.423
7.399
7.355
7.331
7.306
7.280
7.260
7.236
7.212
7.110
7.087
7.064
4.614
4.591
4.568
4.545
1.647
1.623
N
N
O
5
1.00
1.00
1.19
1.07
2.07
2.09
1.00
1.21
1.08
3.18
f1 (ppm)

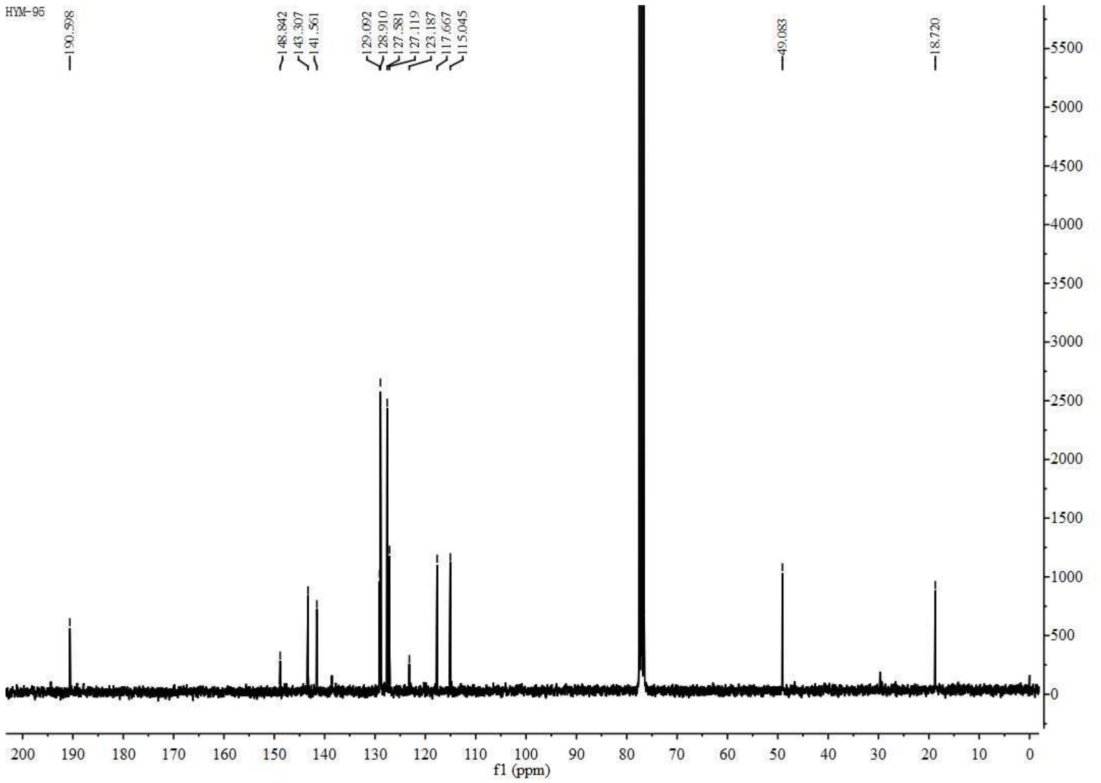
HYM-95
190.598
148.842
143.307
141.561
129.092
128.910
127.581
127.119
123.187
117.667
115.045
49.083
18.720
f1 (ppm)

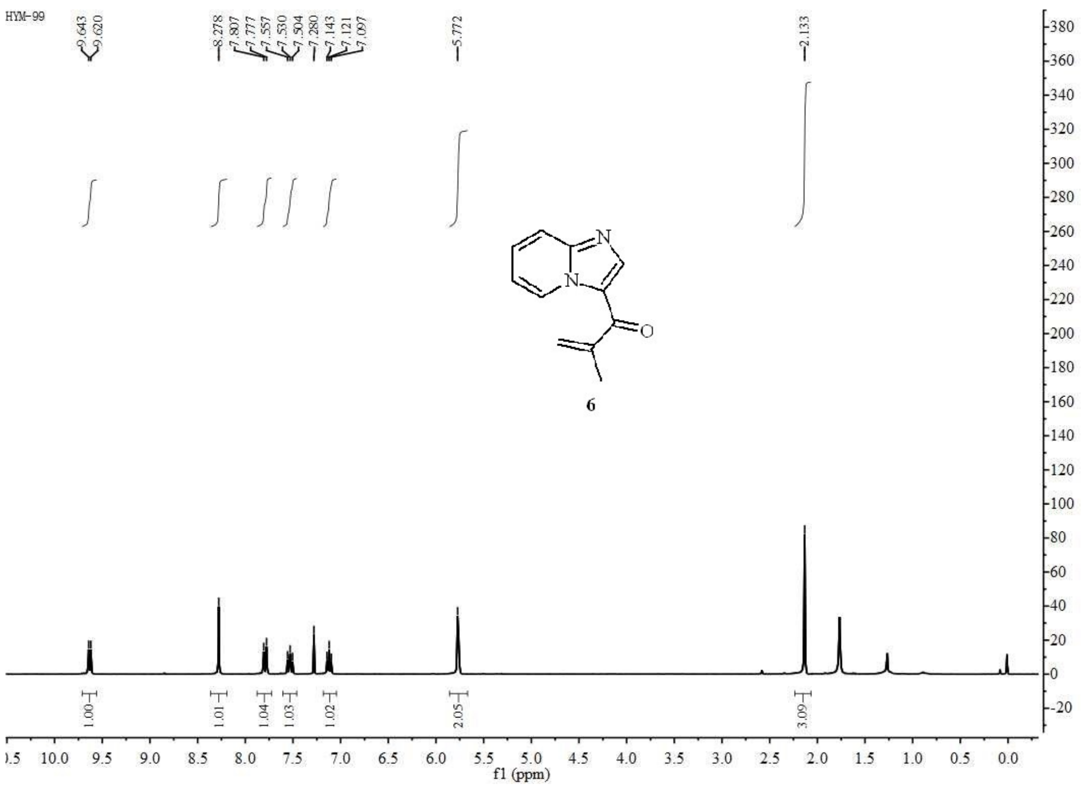
HYM-99
9.643
9.620
8.278
7.807
7.777
7.557
7.530
7.504
7.280
7.143
7.121
7.097
5.772
2.133
6
1.00
1.01
1.04
1.03
1.02
2.05
3.09
f1 (ppm)

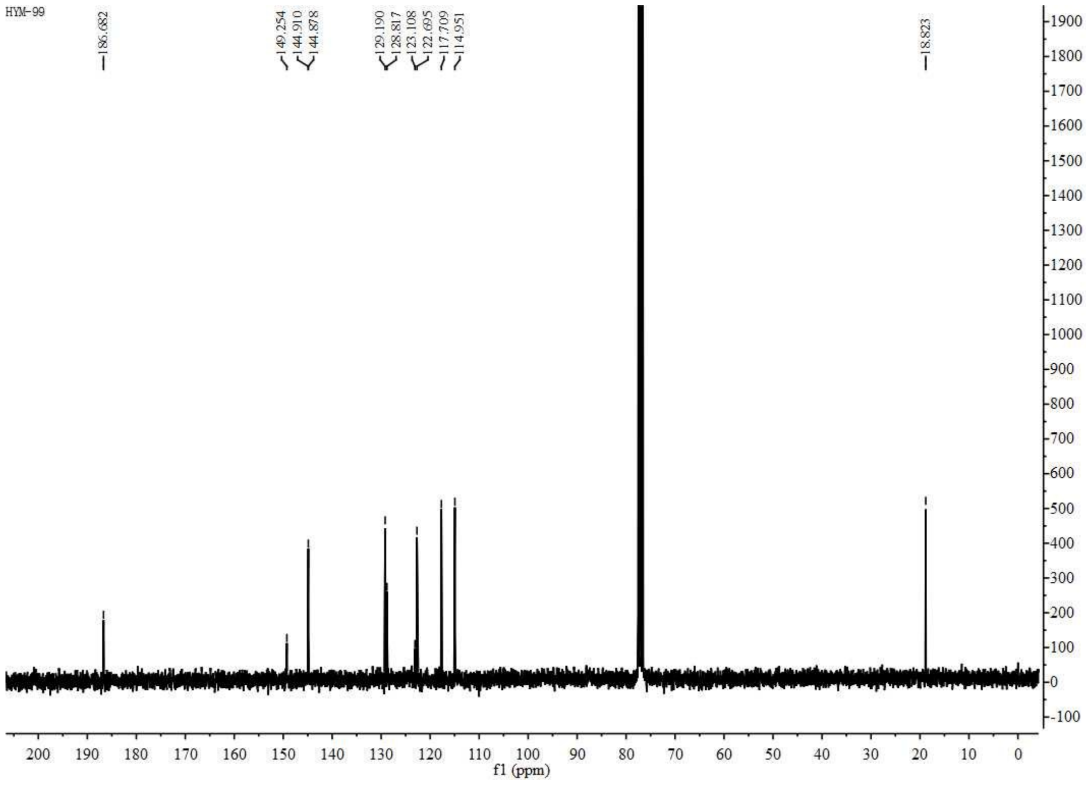
HYM-99
186.682
149.254
144.910
144.878
129.190
128.817
123.108
122.695
117.709
114.951
18.823
f1 (ppm)

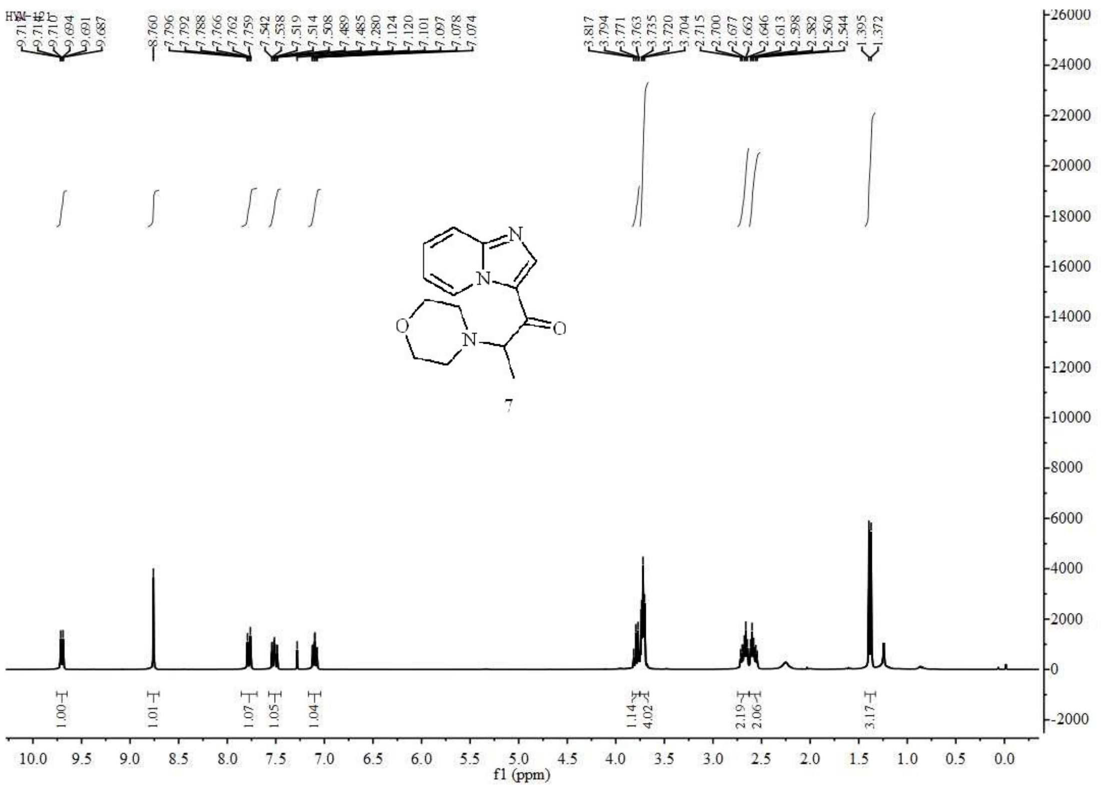
f1 (ppm)
7

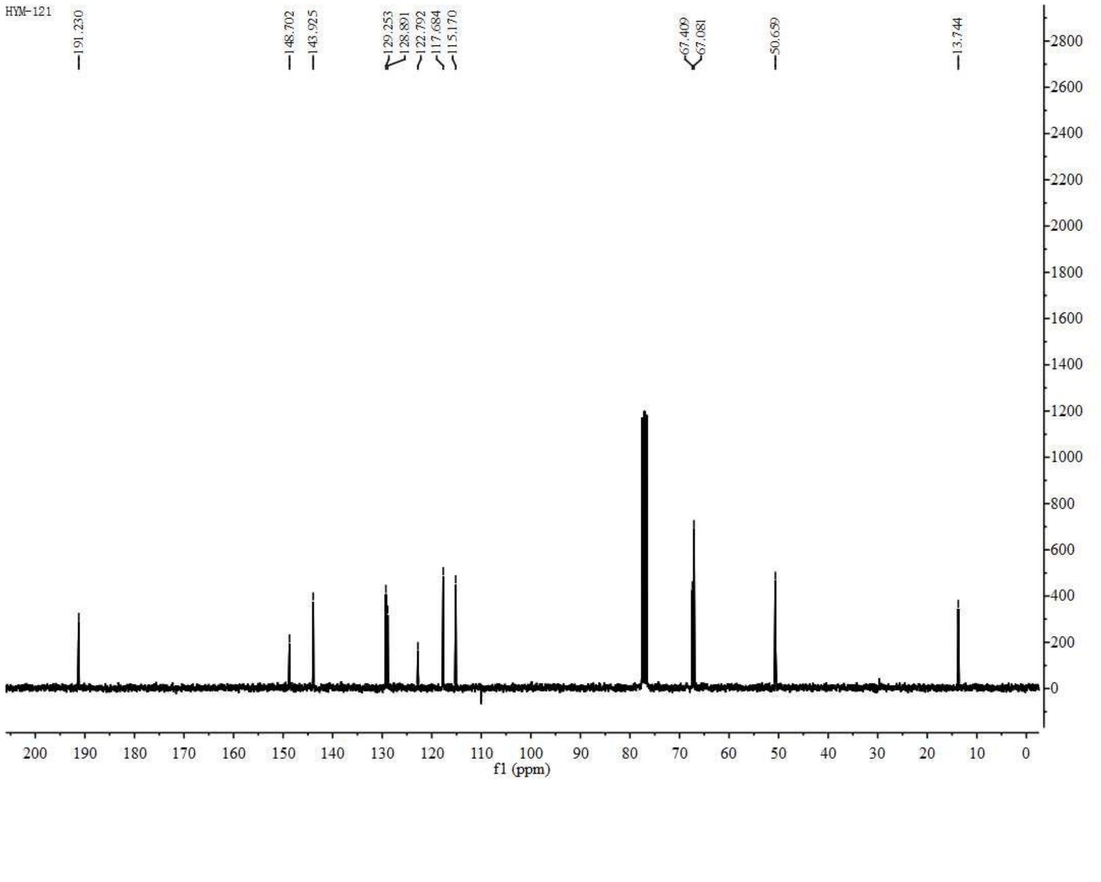
HYM-121
f1 (ppm)

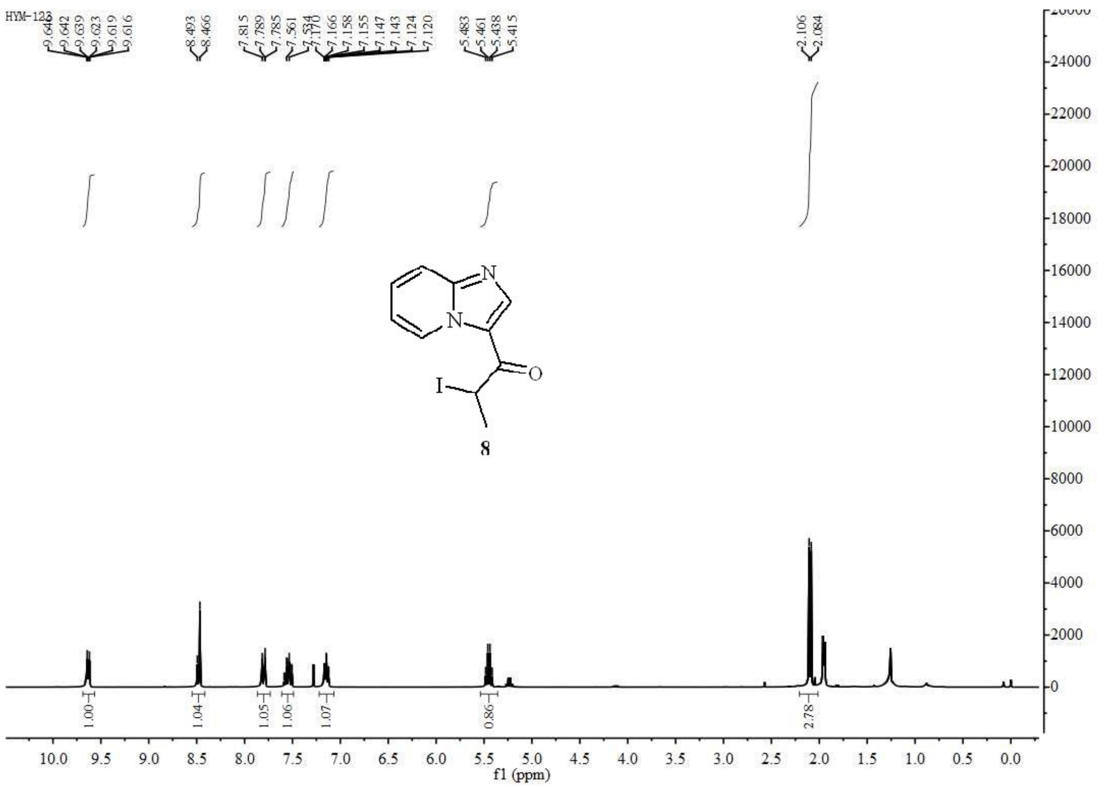
HYM-123
9.642 9.642 9.639 9.623 9.619 9.616
8.493 8.466
7.815 7.789 7.785 7.561 7.534 7.170 7.166 7.158 7.155 7.147 7.143 7.124 7.120
5.483 5.461 5.438 5.415
2.106 2.084
N
N
O
I
8
1.00
1.04
1.05
1.06
1.07
0.86
2.78
f1 (ppm)

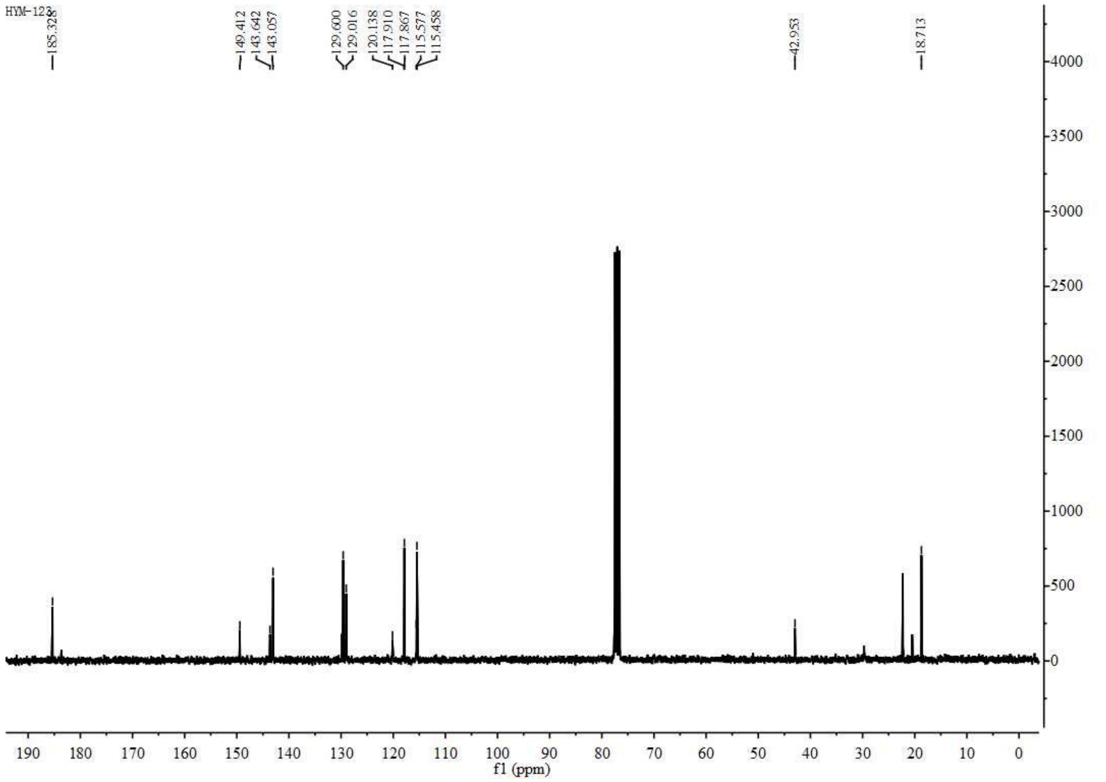
HYM-123
185.328
149.412 143.642 143.057
129.600 129.016 120.138 117.910 117.867 115.577 115.458
42.953
18.713
f1 (ppm)
